# Community Resource for Innovation in Polymer Technology (CRIPT): A Scalable Polymer Material Data Structure

**DOI:** 10.1021/acscentsci.3c00011

**Published:** 2023-02-20

**Authors:** Dylan J. Walsh, Weizhong Zou, Ludwig Schneider, Reid Mello, Michael E. Deagen, Joshua Mysona, Tzyy-Shyang Lin, Juan J. de Pablo, Klavs F. Jensen, Debra J. Audus, Bradley D. Olsen

**Affiliations:** † Department of Chemical Engineering, Massachusetts Institute of Technology, 77 Massachusetts Avenue, Cambridge, Massachusetts 02139, United States; ‡ Pritzker School of Molecular Engineering, University of Chicago, Chicago, Illinois 60637, United States; § Materials Science and Engineering Division, National Institute of Standards and Technology, Gaithersburg, Maryland 20899, United States

## Abstract

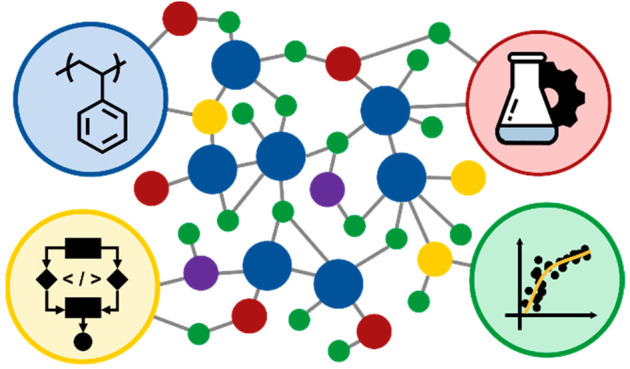

The Community Resource for Innovation in Polymer Technology (CRIPT) data model is designed to address the high complexity in defining a polymer structure and the intricacies involved with characterizing material properties.

## 1 Introduction

Polymers have transformed the ways we heal, feed, clothe, shelter, and transport humanity,^1−4^ and their continual improvement remains a core scientific endeavor.^5−9^ Despite the advent of electronic publishing and electronic lab notebooks, the way we record, store, and share scientific data follows largely the same format as it has for many decades.^10−14^ The scientific community has recognized the need for improved data infrastructure which has led to the FAIR (findable, accessible, interoperable, and reusable) guiding principles to spur knowledge discovery and innovation by extending the longevity and repurposing of digital research assets.^15,16^ The challenge of representing and indexing polymers has hindered the development of data infrastructure, leading to small and disparate data sets or uncatalogued data.^17−20^ As a result, most polymer data are scattered across millions of articles and journals in multiple noninteroperable formats.^21−29^ The inaccessibility of metadata and data leads to massive inefficiencies and missed opportunities to solve many of our current and future problems with a simple search.^30^ Overall, the challenges mentioned above highlight the need for information solutions that make valuable research data discoverable and accessible.

**Figure 1:**
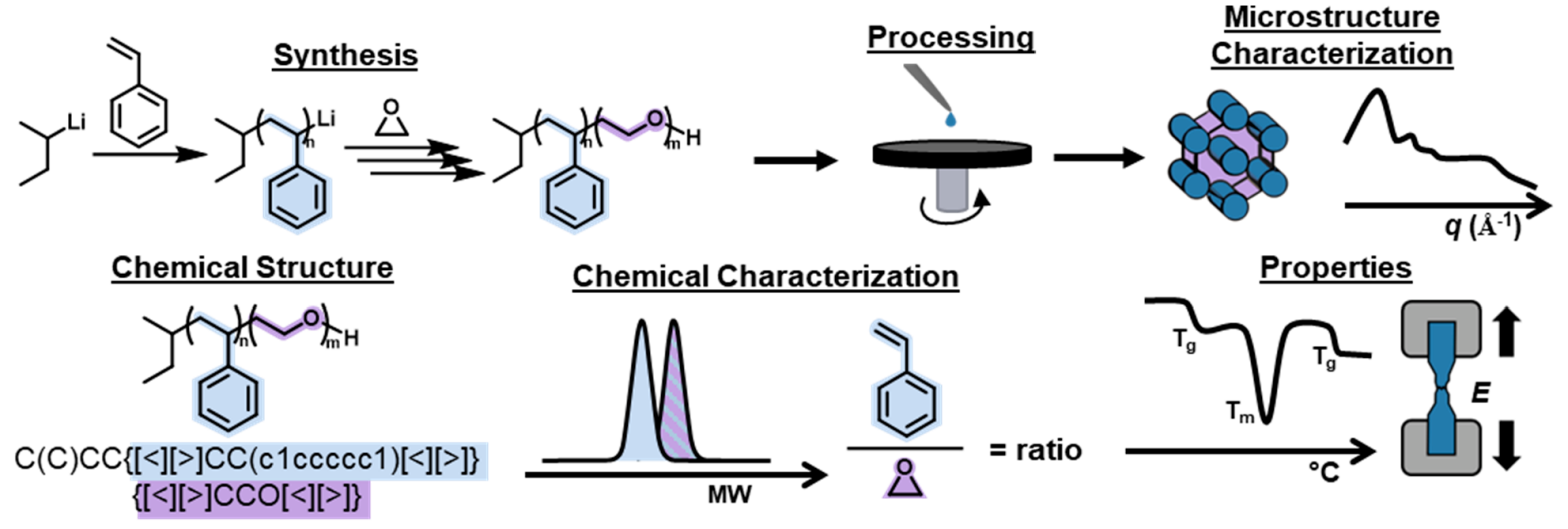
Depiction of the possible range of metadata and data for an example polymer: poly(styrene – block – ethylene oxide) copolymer. This includes synthesis of the polymer, processing (spin coating), microstructure, microstructure characterization (grazing incidence small-angle X-ray scattering), a computer friendly structural representation (BigSMILES), chemical characterization (molecular weight distributions and composition), and properties (differential scanning calorimetry data providing glass and melt transition temperatures, tensile test to measure Young’s modulus).

Among the main barriers to developing information solutions for polymers is the fact that they are large stochastic molecules with hierarchical structures spanning multiple length scales from chemical bonds to large molecular assemblies (Figure 1).^11,31,32^ To date, there is no single representation that can fully define a polymeric material structure, thus making them hard to index.^18^ The stochastic nature of these chemical structures stems from the statistical chemical reactions that are used to produce them. This stochasticity brings about distributions in molecular mass, composition, and topology. A combination of structural descriptors (like a chemical drawing) and distribution information is therefore required to fully define the molecular structure. At larger length scales, microstructures develop from phenomena such as phase segregation and crystallization. These microstructures can be spatially ordered, disordered, or have local patches of order and disorder. The combination of multiple chemical descriptors and length scales of structure makes it extremely difficult to define these materials.

In practice, defining a polymeric structure and characterizing material properties are even more challenging as polymers have an extremely wide range of properties that often are not easily measured due to physical limitations (e.g., due to poor solubility).^31,33^ This leads to variable data availability, often providing only relative information or relying on theoretical models which require expert knowledge to put into context. In many cases, experimentally obtaining the desired information is impossible or intractable, and multiple partial correlative data points must be compiled to provide a surrogate value. Additionally, the processing history under which the material was made can strongly influence microstructure formation and properties. Thus, data sets that do not completely capture the relevant data and metadata will be ineffective at providing reliable information which severely hinders scientific efforts.

With the importance of polymers, there have been several initiatives to capture polymer materials data.^18,22−26,28,29,34,35^ Among the key technical innovations needed for success is a data model or blueprint for data organization. Among the simplest and most prevalent schemas for polymers has been the single table schema, where data are structured in a series of rows and columns.^21,28,34^ This type of schema is often implemented with polymer names as the key structural descriptor followed by a series of properties. In polymer science, name-based identification has limited capability in specifying a molecular structure, making attributing material properties to a structure ambiguous. These types of data sets tend to be small, focused on a limited set of common commercial polymers and properties. The next evolution in database schemas was the migration to single-document schemas.^18,36−38^ These databases store data in ‘documents’ sometimes called objects (i.e., scripted data interchanging formats, typically JavaScript Object Notation (JSON)) which encapsulate data and metadata relative to something of interest. For example, PolyDAT^18^ focuses its document on a single polymer of interest. These single document style schemas represent a significant improvement from table-based schemas as they introduce flexibility in what data can be stored. However, a major drawback is that the documents need to be organized around an object of interest (e.g., material or reaction of interest). Multidocument, graph, and relational styled databases have emerged as the next evolution in data schemas where data can be linked across documents, thus reducing the duplication of data and significantly improving the provenance of data.^29,39,40^ These data schemas allow for increased flexibility and robustness and contain many features that are essential for storing complex data at a large scale. Another key innovation has been the development of BigSMILES, which extends simplified molecular-input line-entry system (SMILES), a compact line chemical line notation for polymers.^41^ BigSMILES provides a human and computer interpretable structural representation that can be used as a key identifier for polymer data.

To address the need for a scalable polymer informatics solution, this work details the Community Resource for Innovation in Polymer Technology (CRIPT) data model. The goal of the CRIPT is to develop a community-driven data ecosystem for polymer science. At the core of CRIPT is a general graph data model that places an emphasis on capturing the metadata and data needed to accurately represent the complexities of polymer material. More specifically, CRIPT’s data structure is designed to capture everything from small-molecule and polymer synthesis, material processing, material and reaction properties, material characterization, raw experimental data, and both atomistic and coarse-grained simulations of systems with well-defined chemistries. The data model focuses on providing a highly granular and descriptive design with a strong aversion to ambiguity while seeking to be as comprehensive as possible. CRIPT is driven by FAIR^15,16^ and open-source principles^42^ to support its community driven mission. The following sections will initially cover the design philosophy and technical aspects that drove the construction of the CRIPT data model. This will be followed by a high-level overview of the main nodes that make up the CRIPT data model and how they are linked to form the graph structure. A few examples will be provided to demonstrate how the data model operates. Finally, aspects of the implementation of the data model are discussed. The Supporting Information contains a detailed discussion for each node and subobject along with additional examples.

## 2 Results And Discussion

### 2.1 Design Features/Philosophy

CRIPT’s data structure is a graph consisting of sets of vertices/nodes, that contain the stored data/metadata, and edges, which store the relationships between data. Every node has a series of attributes and subobjects. Subobjects provide a hierarchy for organization and grouping data within a node (see Supporting Information for details), and attributes are the individual pieces of information that are to be stored. The links between nodes (edges) are achieved with a globally unique and persistent identifier. The presence of the unique identifier of one node in another node signifies an edge between those two nodes in the graph.^29,39,40^

The flexibility of CRIPT stems from the arrangement of nodes in a wide range of configurations, and nodes themselves can be reconfigured. The high level of granularity of CRIPT can be seen in the storage of any type of process, property, and data with explicit specification of context. For example, chemical properties can be explicitly attributed to a component in a mixture, or even more granularly, the property can be associated with a fragment of a chemical structure with atom indexing within BigSMILES. CRIPT reduces ambiguity by providing and directing data entry through an ever-growing and curated controlled vocabulary. Data validation is an additional design layer that has been added to minimize ambiguity by ensuring uniformity in the entered data. The combination of these design features sets the stage for a robust and general material data model.

The features that make the CRIPT’s data model uniquely suited for polymers are the inclusion of process history, support for BigSMILES, and the integration of both experimental and computational data. Capturing process history is a critical detail as physical properties such as elastic modulus, toughness, transparency, etc. depend strongly on processing history. Thus the ‘same polymer’ can have a wide range of values for a property given its processing conditions. Moreover, the graph architecture supports the need to link characterization data across multiple synthesis and processing steps to enable the definition of complex polymer architectures. CRIPT directly supports BigSMILES as the main structural descriptor for polymers since it is human readable, machine-friendly, and has full support for the wide diversity of polymer structures.^41^ BigSMILES in the context of CRIPT can be viewed as defining an ensemble of possible structures for a stochastic polymer where the probability of observing each molecular state is specified with the additional properties captured by the data model (molecular mass, dispersity, etc.). The inclusion of BigSMILES provides an opportunity for polymer structural search as well. CRIPT’s data model takes the stance that both data from experiments and simulation should be supported at an equal level, improving cohesion of the research community and the breadth of accessible data.

The design of a data model for an entire community brings about several technical challenges regarding database robustness, performance, maintainability, and cost. CRIPT’s graph structure provides robustness by not storing the data relative to a reaction, material, or organizational approach. This enables a single user or multiple different users to enter data which will yield the same representation in the data model. One of the key performance considerations is maintaining fast searching as the size of the database grows, while the data model facilitates rapid graph traversal, indexing by node type, and other advanced database search solutions. Additionally, the reduction of duplicated data through referencing significantly lowers the amount of data that needs to be stored and searched, and persistent identifiers enable reusability. As with any data structure, the design will continue to evolve, and the modularity of the graph structure simplifies maintainability and extensibility as changes are isolated to each individual object, minimizing the cost of reworking/adding improvements (the growth of technical debt).

CRIPT desires to be comprehensive, but it is impractical to store the large and growing amount of data within CRIPT directly. To navigate this issue, CRIPT’s data model embraces federated data storage. Federated data storage is a more attractive alternative to one monolithic server, as the aggregation of all data into a single location is slow, and the database resources are consumed by moving large files around, making these systems much more resource intensive and costly.^43^ Support for federated data is realized in CRIPT’s data model by focusing on storing key values (such as property data, material identifiers, and processing information) and metadata relevant for discoverability (typically a uniform resource locator (URL)) within the data model while directing nonkey information (such as raw data files) to be stored elsewhere. This enables users to store their data on their preferred data services (like Amazon Web Services^48^ or university servers). The federated database architecture helps to support the community and a FAIR-driven mission by allowing for decentralization of data.^15,16^

### 2.2 Data Model

CRIPT’s data model has two levels of structuring: nodes, which are the primary objects that make up the CRIPT graph structure (nodes will be written in *italics*), and subobjects which are used to construct substructures within the nodes (see Supporting Information for details). The *project*, *collection, experiment*, and *inventory* nodes serve as an organizational tool within the data model and are represented with a grayscale. The *material*, *process*, *computation, computational_process*, and *data* nodes make the core data-rich nodes which are represented by bold colors. Additionally, there is a *reference* node for citing external sources of data and *software* for citing computational tools (both colored white). A short description and examples of the nodes are shown in Table 1; and detailed explanations can be found in the Supporting Information.

**Table 1:**
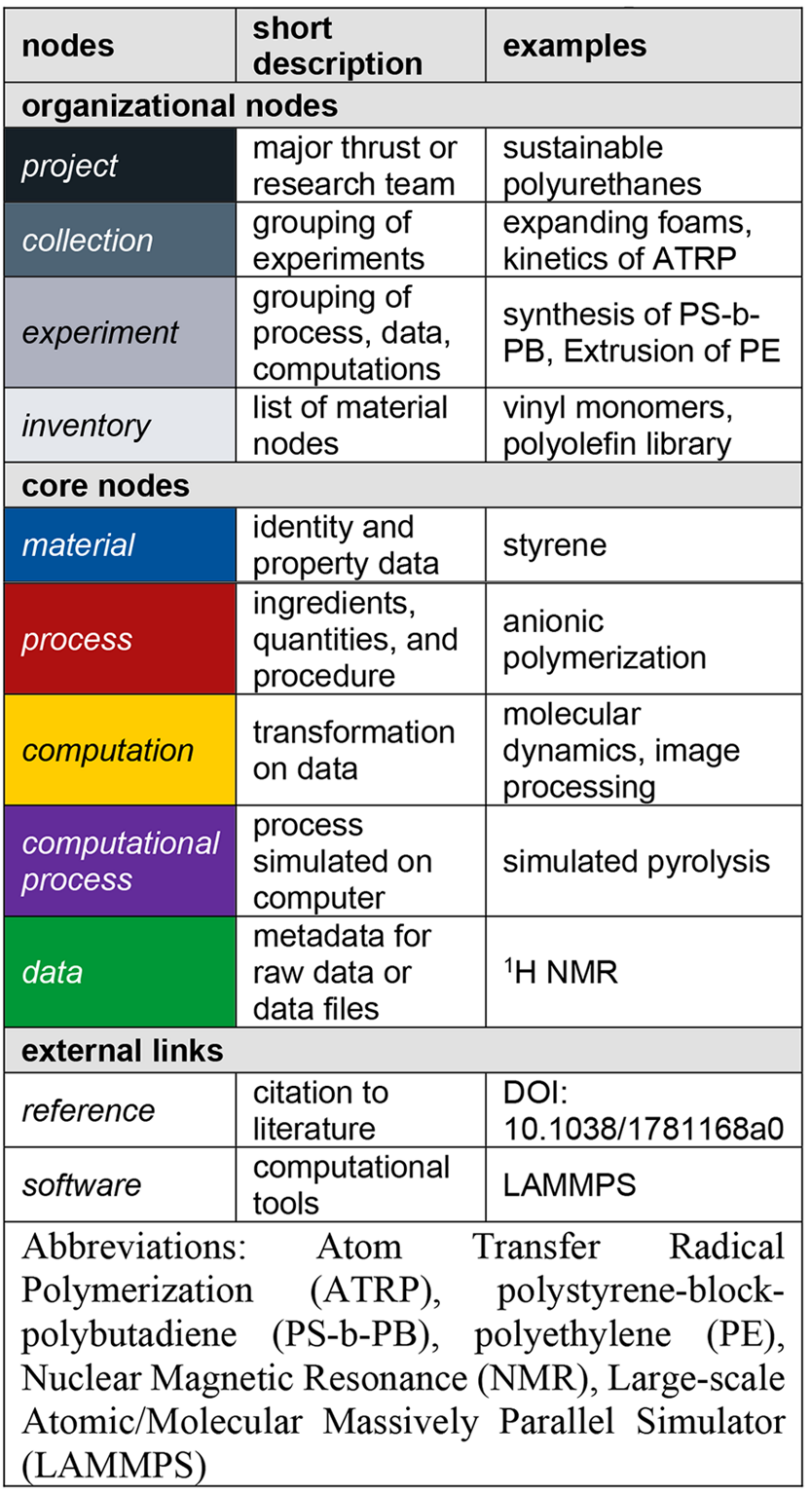
List of CRIPT Nodes with a Short Description and Examples^a^

a Abbreviations: Atom Transfer Radical Polymerization (ATRP), polystyrene-block-polybutadiene (PS-b-PB), polyethylene (PE), Nuclear Magnetic Resonance (NMR), Large-scale Atomic/Molecular Massively Parallel Simulator (LAMMPS).

**Figure 2:**
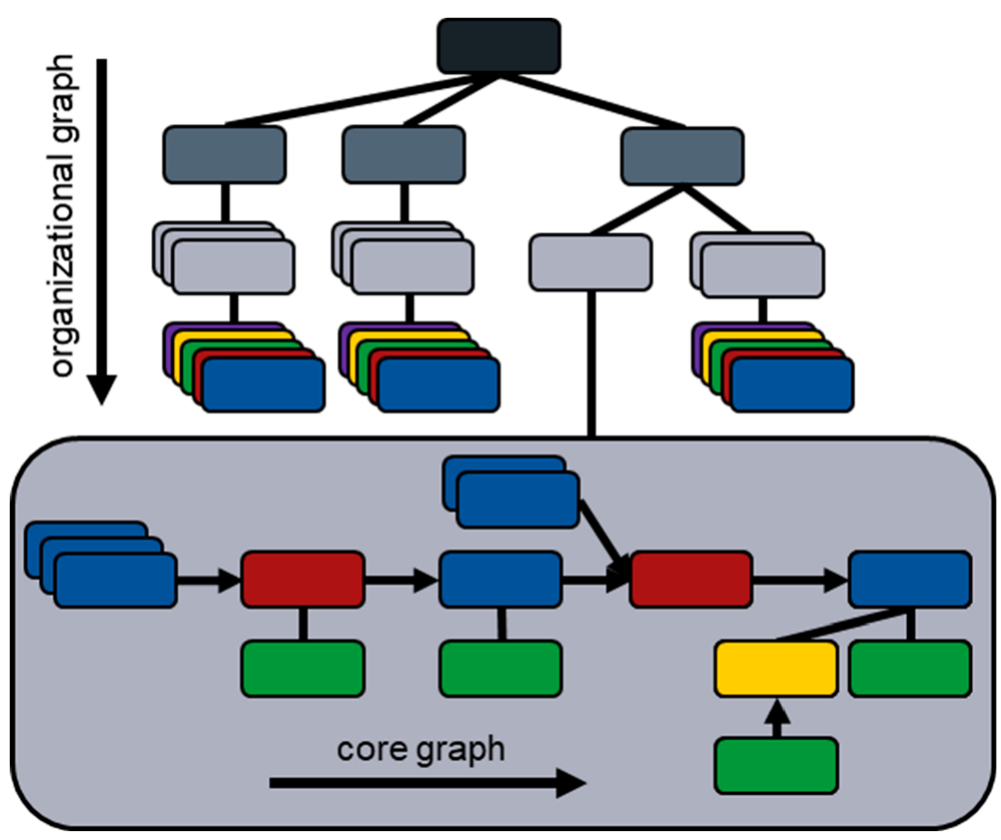
A depiction of the high level graph structure of the CRIPT data model. The organizational nodes (gray nodes) have a tree graph structure and provide a system for indexing and organizing the complex data graph. The core nodes (colored nodes) have a directional graph structure mimicking the temporal relationship between the actual objects as data was generated.

The organizational nodes have a tree like structure (Figure 2) with *project* being the root node that represents a major scientific thrust or research group. The *project* node links to one or more *collections* in which a *collection* is roughly equal to a publication or the content of a final project report. A *collection* links to one or more *experiments* and/or *inventories*. An *experiment* in this context refers to the association of data which can be either a physical experiment in the lab or a simulation. The *inventory* node is a way for users to create a list of materials. *Projects*, *collections*, *experiments*, and *inventories* provide organizational tools to help the users who are entering data into the database.

The organizational graph is independent of the core graph structure, with only unidirectional referencing coming from the organization nodes to the core nodes (Figure 2). Structuring the data in this way makes the core data graph invariant to how users organize data in *collection* and *experiment* nodes. Specifically, a *project* node is linked to *material* nodes, and an *experiment* node is linked to *process*, *data*, *computation*, and *computational_process* nodes. *Materials* are only associated with a single *project* node, and those *materials* can only be used within that *project* to avoid issues of data integrity. For example, if a user referenced a *material* node from another *project* and then the *material* was deleted, this would create a broken reference leading to the loss of data integrity. Thus, the reuse of *materials* from other *projects* requires copying *material* nodes into the current *project*. *Process*, *data*, *computation*, and *computational_process* nodes all link to a single *experiment*. Overall, this allows users to organize the *process*, *data*, *computation*, or *computational_process* as they see fit without changing how the data is stored in the database. These design choices seek to minimize the unexpected loss of data integrity without creating a large user or infrastructure burden.

**Figure 3:**
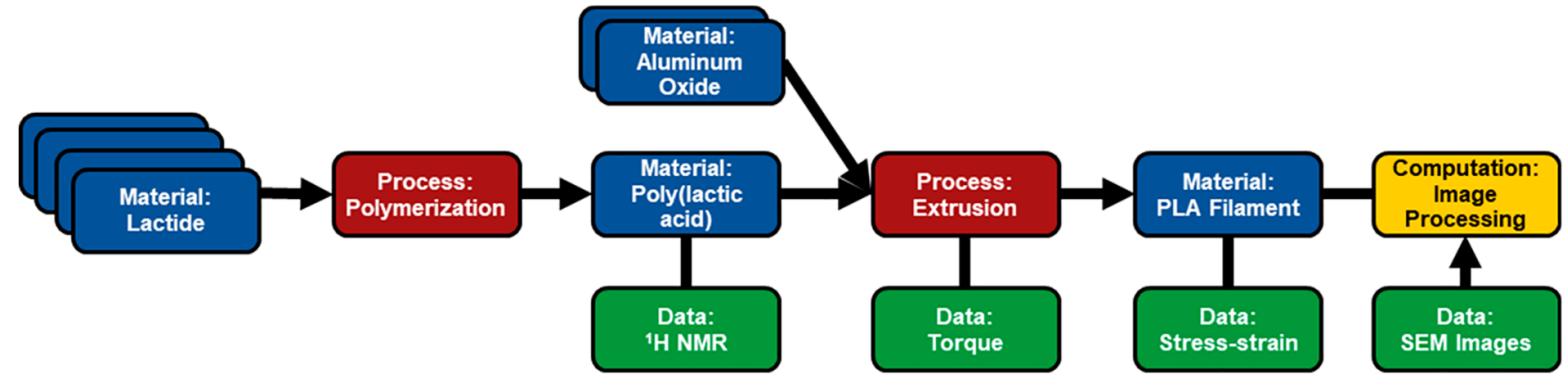
Example of a core graph for poly(lactic acid) (PLA) filament. This example highlights a chemical synthesis (first *process* node) to produce PLA, followed by an extrusion process to produce PLA filament (second *process* node). Raw data are recorded at multiple stages of the experiment to characterize material and chemical properties, and computations are used to convert the scanning electron microscopy (SEM) images into material properties.

The core graph structure is highly variable depending on the experiment; however, all wet-lab experimental graphs start with defining a *material* node or set of *material* nodes. A *material* is a collection of the identifiers and properties of a chemical, mixture, or substance. For example, in a typical chemical synthesis (Figure 3), the set of *materials* that are first defined are the ingredients for a *process* node. The *process* node contains details about quantities, procedure, process conditions, equipment, etc. for a material transformation. A *process* node may represent chemical transformation (e.g., chemical reaction), physical transformations (e.g., extrusion), or sample preparation for characterization. An alternating pattern of *material* and *process* nodes serves as the backbone of the experimental graph with *data* or *computation* nodes attached to the *materials* and *processes*. *Data* nodes provide links to sample preparation and to raw or processed experimental data like from nuclear magnetic resonance (NMR), size-exclusion chromatography (SEC), or differential scanning calorimetry (DSC). *Computation* stores information related to the creation or transformation of data. In the example, the *computation* node stores the steps used to calculate material properties derived from the raw data. Multistep chemical processes, including nonlinear diverging and converging processes, are also naturally captured in the core graph structure (see examples ‘Diblock Bottlebrush Synthesis’ and ‘Chemical Reaction with Aliquots’ in Supporting Information).

**Figure 4:**
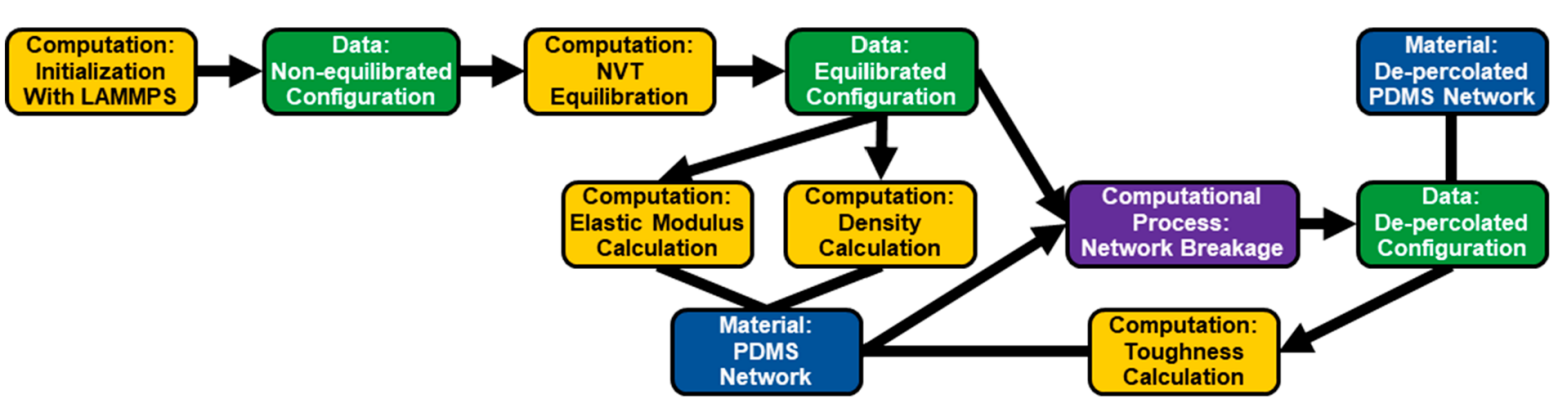
Example of a computational core graph for determining the toughness of a poly(dimethylsiloxane) (PDMS) network under uniaxial extension. The PDMS network is initialized (first *computation* node), equilibrated (second *computation* node), and properties are computed (middle *computation* nodes). The computed properties are stored in the ‘PDMS Network’ *material* node. The material is then taken through a uniaxial extension process (*computational process*) and toughness is computed.

For computational experiments, a typical graph starts with a *computation* (Figure 4) to capture the initialization of the computational system and information regarding the set of procedures related to building the molecular structure, initialization of the simulation box, etc. The initial *computation* node will produce *data* nodes that store the configuration of a virtual material. In the example, the initialization *computation* node produces the configuration file for the unequilibrated state of a polymer. *Data* nodes are then passed into further *computations* to transform the virtual material between configurations (e.g., from nonequilibrated state to an equilibrated state). This pattern of alternating *computations* and *data* nodes is a core motif of the simulation graphs. From a virtual material configuration (*data* node), properties can be calculated (*computation* node) and attributed to a polymer (*material* node). The production of the *material* node places simulated material properties in the same position as experimentally determined material properties. The *computational_process* node is used to capture simulated reactions or physical transformations on virtual materials. The *computational_process* node requires both a *material* node and the corresponding configuration file (data node) as the ingredients of the process will produce a new postprocessing configuration file (data node). This new virtual material configuration will indirectly lead to a new *material* node as properties are extracted.

### 2.3 Data Model Examples

**Figure 5:**
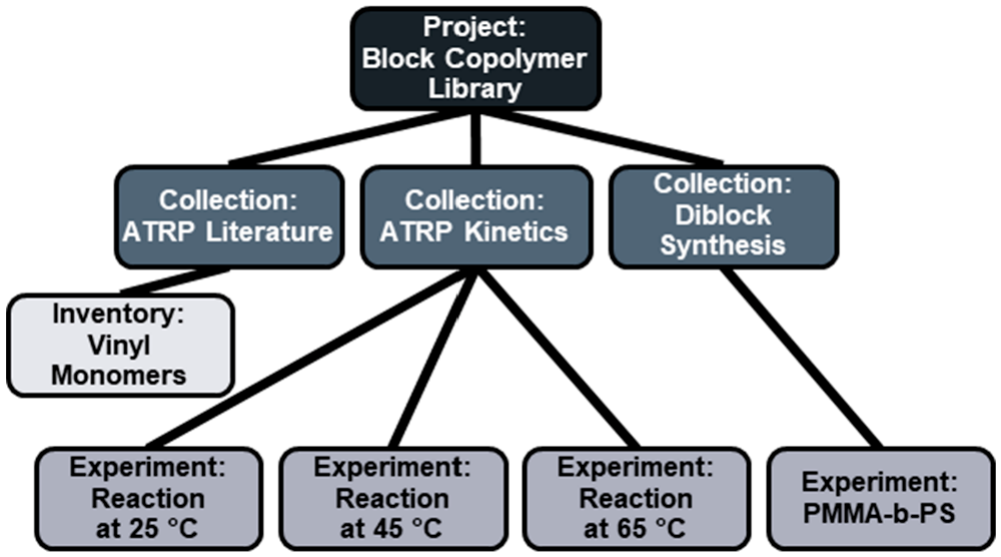
Example graph of the organizational nodes for the creation of a block copolymer library. The ‘block copolymer library’ *project* serves as the root of the organizational graph. Three *collections* are contained within the *project*: ‘ATRP literature research’, ‘ATRP kinetics experiments’, and ‘synthesis of the diblock library’. The ‘ATRP literature’ *collection* consists of an *inventory* of vinyl monomers. The ‘ATRP kinetics’ *collection* consists of 3 *experiments* that were performed at 3 different temperatures to determine optimal reaction conditions. The ‘Diblock Synthesis’ contains a single experiment which is the synthesis of the targeted poly(methyl methacrylate)-block-polystyrene (PMMA-b-PS) polymer.

To illustrate the data model, an example graph centered around the organizational nodes is provided (Figure 5). Every new research effort involves creating a *project* node, in this case ‘block copolymer library’. The first part of the project may involve collecting literature information about how to synthesize the targeted block copolymer. During this process, information about the monomers can be recorded in *material* nodes and collected into an *inventory*, ‘vinyl monomers’ for use in experiments. As the project progresses, kinetic experiments may be performed to determine the optimal reaction conditions to produce the targeted materials. This set of kinetic experiments is grouped into their own *collection*, ‘ATRP kinetics’. Following successful determination of the optimal reaction conditions, the targeted library block copolymers can be made and grouped into their own *collection*, ‘diblock synthesis’. This example highlights a simple graph for the organizational nodes as there are likely to be many more *collections* and *experiments*.

**Figure 6:**
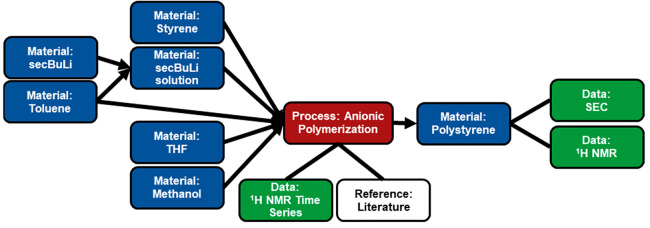
Graph for the chemical synthesis of polystyrene made by the secBuLi initiated anionic polymerization of styrene. The graph starts with ‘secBuLi’ and ‘Toluene’ combining to create a ‘secBuLi solution’ material node. The ‘secBuLi solution’, ‘Styrene’, ‘Toluene’, ‘THF’, and ‘Methanol’ are added to the ‘Anionic polymerization’ *process* which captures the chemical reaction. Polymerization kinetic data were collected for the *process* by ^1^H NMR. The polymerization procedure was inspired by a literature reference. The process yields a ‘Polystyrene’ material node which was characterized by SEC and ^1^H NMR.

For an example graph of a chemical synthesis, the anionic polymerization of styrene with sec-butyl lithium (secBuLi) in a mixture of tetrahydrofuran (THF) and toluene is illustrated in Figure 6.^44^ The chemical synthesis graph is grouped into a single *experiment* node. When defining a new *experiment* for a chemical process, it is recommended to start with the ingredient *material* nodes, followed by a *process* node, product *material* node, and finishing with the characterization *data* nodes. To define the first ingredient in this example, a *material* node for styrene is created by adding identifiers such as SMILES strings, and chemical names. *Material* properties, like density and molecular mass, etc. are also added to aid with calculating reagent amounts. Mixtures such as secBuLi in toluene can be represented by making the two pure material nodes (‘secBuLi’ and ‘Toluene’) followed by making a third material node which is a mixture of the two. With all the ingredients defined, the *process* node for the anionic polymerization is defined by specifying the quantity of each ingredient, experimental procedure, conditions (reaction time, temperature), and reaction properties (yield). In addition to experimental data, this example was inspired by a literature reference, which can be linked directly to the *process* node through a *reference* node. With the *process* defined, the product *material* node ‘Polystyrene’ can now be created by specifying the identity with BigSMILES and a chemical name. Property data such as number average molecular mass and dispersity can be added, and the raw data for both the ^1^H NMR and size exclusion chromatography (SEC) analysis can be attached through the creation of *data* nodes. In this case, the combination of a BigSMILES string and the molecular mass data from SEC provide a full definition of the polymer structure.

**Figure 7:**
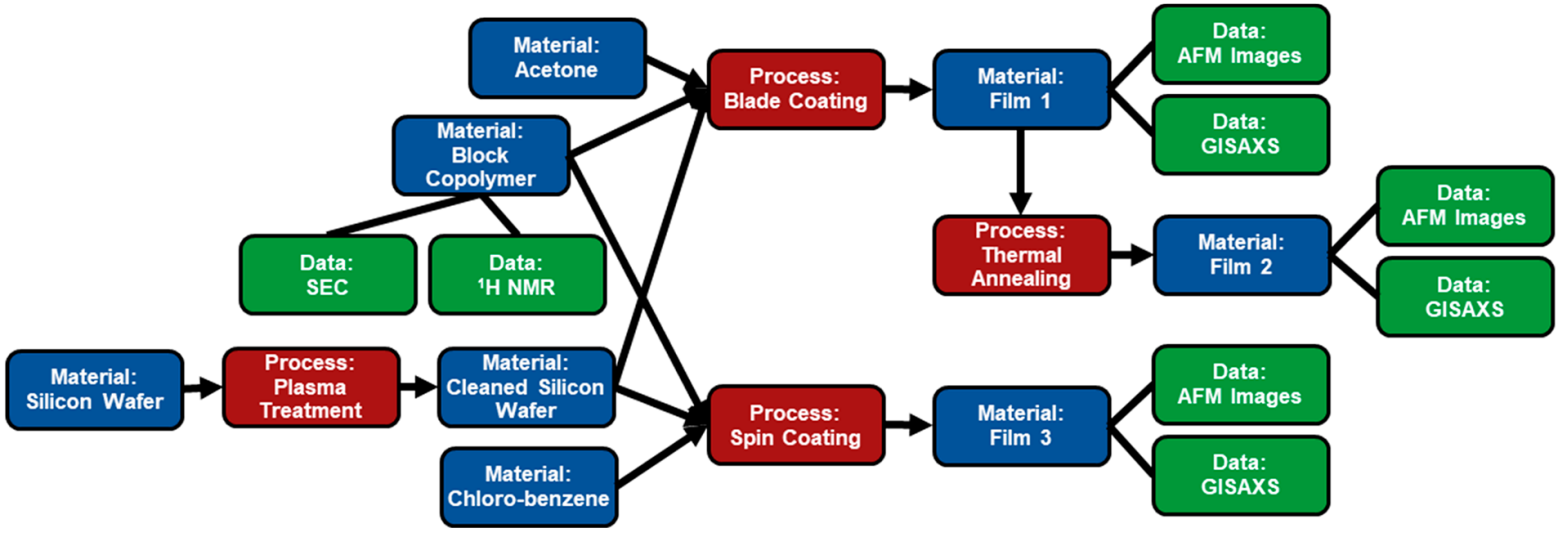
Graph for the characterization of block copolymer films prepared by blade and spin coating. Starting on the left side of the figure, silicon wafers are cleaned by plasma treatment. The block copolymer was obtained from a vendor and characterized prior to the formation of films. Film 1 was generated by dissolving the block copolymer in acetone and blade coating the solution onto a cleaned silicon wafer. Film 2 was prepared by thermal annealing film 1. Film 3 was generated by dissolving the block copolymer in chlorobenzene and spin coating the solution onto a cleaned silicon wafer. All three films were characterized by AFM and GISAXS.

A graph illustrating the investigation of the self-assembling behavior of a block copolymer in thin films (Figure 7) shows the applicability of the data model to polymer characterization. The block copolymer of interest was obtained from a vendor; thus, it was initially characterized with SEC and NMR prior to use. To prepare for the investigation, atomically smooth silicon wafers were cleaned with a plasma treatment and will serve as the substrate for the block copolymer assembly. The first processing approach was to dissolve the block copolymer in acetone and perform a blade coating process. This sample was characterized by AFM (atomic force microscopy) and GISAXS (grazing-incidence small-angle X-ray scattering) to determine the microstructure phase and domain spacing. This same sample was then thermally annealed and recharacterized with the same techniques. Following these studies, another film was produced from the original block copolymer sample by dissolving the sample in chlorobenzene and spin coating the solution onto the silicon wafer. The AFM and GISAXS were once again performed to characterize the microstructure.

**Figure 8:**
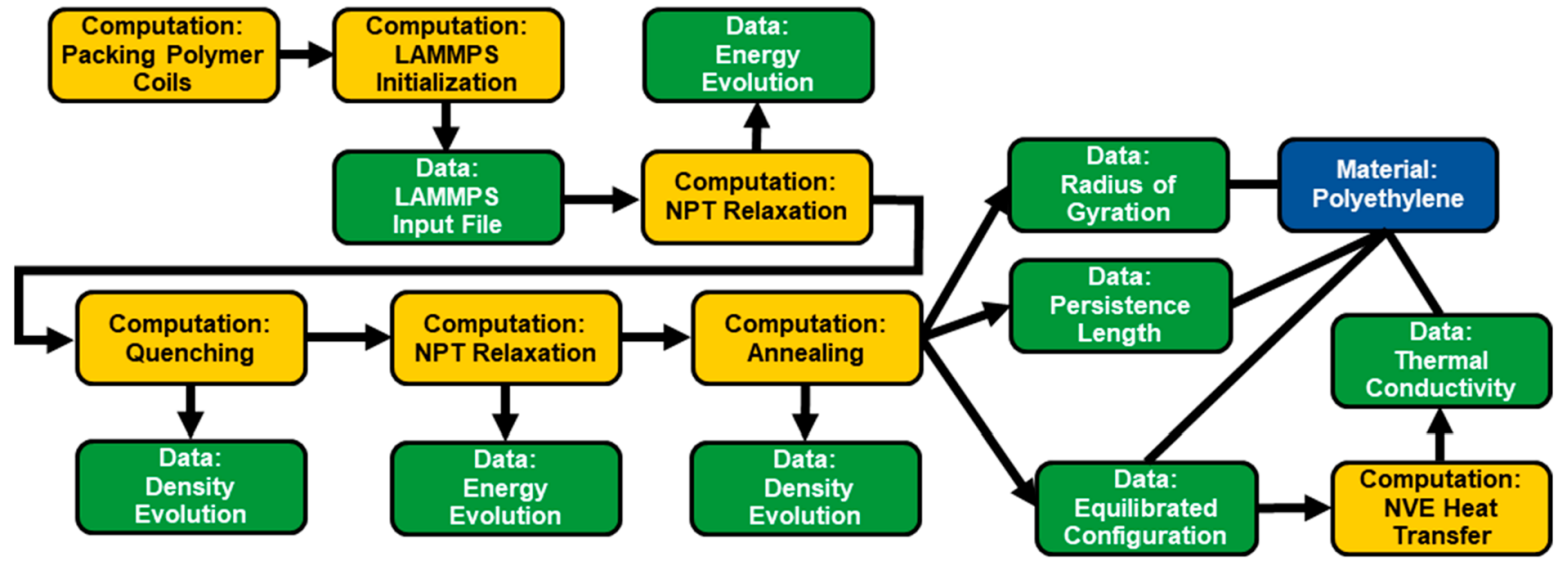
Graph for the thermal conductivity analysis of polyethylene using LAMMPS. The simulation graph starts by defining the packing of polyethylene coils in a simulation box within a *computation* node. After initialization, the equilibrated polymer configurations were obtained with a procedure to relax, quench, and anneal the simulated ensemble. The resulting equilibrated properties, including radius of gyration and persistence length, were computed. An additional heat transfer simulation was carried out to measure the thermal conductivity.

Computational characterization of bulk amorphous polyethylene via molecular dynamics simulations is illustrated in Figure 8.^45^ The simulations are conducted using the LAMMPS software with an input file generated by packing polymer chains in a simulation box. After an equilibration procedure with a series of steps to relax, quench, and anneal the system, the radius of gyration, persistence length, and thermal conductivity of the equilibrated polyethylene are measured. For the thermal conductivity measurement an additional heat transfer simulation was performed.

**Figure 9:**
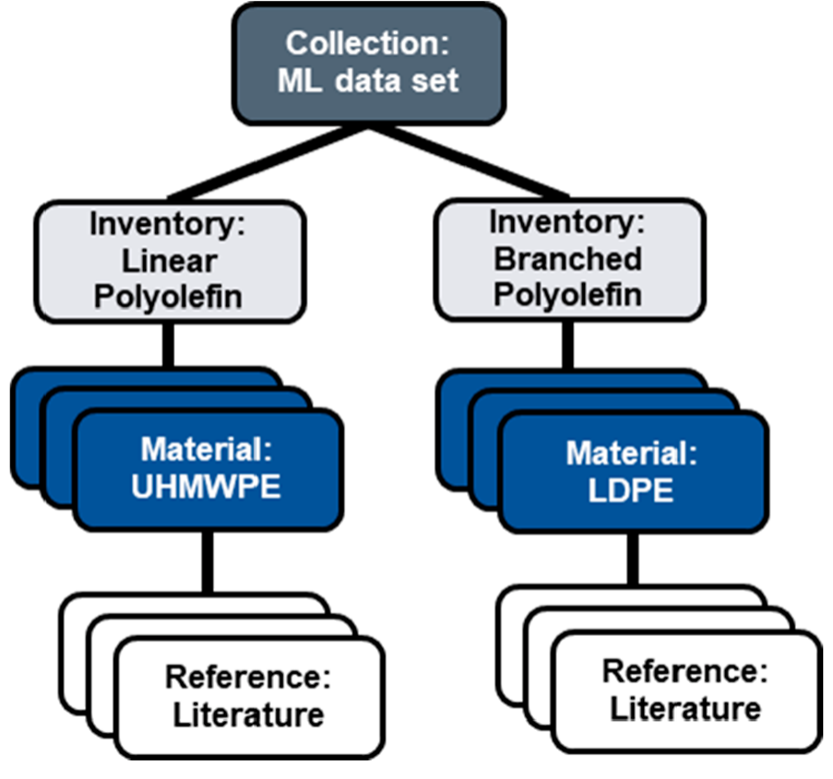
Graph for the creation of a machine learning data set for the prediction of linear and branched polyolefin material properties. Material properties are stored in the *material* node and can be cited to the literature reference with a *reference* node. *Inventory* nodes can be used to group *material* nodes into subdata sets which can be linked to a single *collection* to group the entire data set together. Abbreviations: ultrahigh molecular weight polyethylene (UHMWPE), low density polyethylene (LDPE)

The following example depicts a graph for the extraction of polyolefin material properties from the literature for machine learning (Figure 9). The extracted data can be directly stored in the properties section of the *material* nodes, and then the material nodes can be organized into subdata sets with the use of *inventories*. The entire data set can be organized into a single *collection*. Citations back to the literature source can be made on a data point basis with the use of *reference* nodes.

### 2.4 Implementation

To put the data model into practice, a Python^46^ software development kit (SDK) has been implemented and made available for download.^49^ The purpose of the Python SDK is to streamline the use of the data model, as manually writing data into the data model would be complex, time-consuming, and error-prone. To handle the referencing between nodes, globally unique and persistent identifiers are autogenerated for each node and used to provide bridges between nodes. In CRIPT the persistent identifiers are URLs as CRIPT is natively web-based (and representational state transfer (REST) compatible), although the use of URLs is not a requirement. Each node can be serialized for storage in any desired format (typically JSON) and transferred across various hardware, software, databases, and programming languages. The Python SDK also provides an opportunity to incorporate various data validation layers, such as type check, data ranges, unit dimensionality, and canonicalization to increase data uniformity. The Python SDK is written in such a way that additional validation methods can be smoothly added as the software evolves.

To extend the data model for implementation into a full software ecosystem, two additional nodes are included in the data model, *user* and *group*, whose purpose is to provide access control to data. A *user* node is created when an individual joins the CRIPT ecosystem and stores their user information. Among one of the key *user* attributes is ORCID (open researcher and contributor ID) ID which provides a unique and persistent digital identifier back to a specific person.^47^ This serves to ensure that all contributions to the database are appropriately attributed to the individual. Additionally, the ORCID ID can be used for login through the ORCID API (application programming interface). A *group* is an organization of multiple *users,* and a *user* can be part of multiple *groups*. The *group* node is where access control/ownership for all data lies, and the *group* node will point to all other nodes in the CRIPT data model. The decision to make *groups* the owner of data was motivated by users tending to change jobs, research groups, and organizations throughout their careers, and data are typically owned by the organization and not the individual. In the simplest case, *group* and *project* will have a one-to-one relationship; and the one-to-one relationship will only be broken when more granular access control is needed (see Supporting Information example ‘Across Control Within Projects’). The inclusion of these nodes serves to provide a key feature needed to link to user directories common in large organizations. For data that are desired to be shared with the whole community, the ‘public’ attribute can be set to true to enable the data’s inclusion in the public search.

## 3 Conclusion

This work defines a new graph data model that underpins the CRIPT digital ecosystem. The data model is designed to support the structuring of metadata and data for polymers from physical experiments, and both atomistic and coarse-grained simulations of systems with well-defined chemistries. The graph data structure provides flexibility as well as granularity in the data it represents. The connections in the graph provide an intuitive model for the typical material research workflow and allow for high-level information to be swiftly gleaned. Considerations were placed on designing the CRIPT data model to (1) scale for big data while maintaining efficient searching and (2) reduce duplicated data which significantly lowers the amount of data that needs to be stored. This graph-based approach to modeling material data provides the key advancements that the community needs to bring cheminformatics to polymers. Overall, having well-structured data will lead to new innovations and enable the rapid sharing of data and innovations across the scientific community.

## Supporting Information Available

A detailed discussion for each node, subobject, and additional examples (PDF)
Full data model representation for example found in Figure 6 (PDF)
