# Supplementary material for: Community Resource for Innovation in Polymer Technology (CRIPT): A Scalable Polymer Material Data Structure
Source: ACS Cent Sci. 2023 Feb 20;9(3):330–8. doi: 10.1021/acscentsci.3c00011 (PMC10037456; doi:10.1021/acscentsci.3c00011)
Supplement: Supplementary file 1 — oc3c00011_si_001.pdf [file oc3c00011_si_001.pdf]

Supporting Information for:

Community Resource for Innovation in Polymer Technology (CRIPT): A Scalable Polymer Material Data Structure

Dylan J. Walsh[a], Weizhong Zou[a], Ludwig Schneider[b], Reid Mello[a], Michael E. Deagen[a], Joshua Mysona[b], Tzyy-Shyang Lin[a], Juan J. de Pablo[b], Klavs F. Jensen[a], Debra J. Audus[c], Bradley D. Olsen*[a]

[a]Department of Chemical Engineering Massachusetts Institute of Technology, 77 Massachusetts Avenue, Cambridge, Massachusetts 02139, United States

[b]Pritzker School of Molecular Engineering, University of Chicago, Chicago, IL 60637, USA.

[c]Materials Science and Engineering Division, National Institute of Standards and Technology, Gaithersburg, Maryland 20899, United States

TABLE OF CONTENTS:

1. CRIPT Nodes **4**

1.1. Base Attributes *7*

1.2. Project Node *7*

1.3. Collection Node *8*

1.4. Experiment Node *9*

1.5. Inventory Node *9*

1.6. Material Node *10*

1.7. Process Node *11*

1.8. Data Node *12*

1.9. Computation Node *13*

1.10. Computational_process Node *14*

1.11. Reference Node *15*

1.12. Software Node *16*

2. Sub-Objects **17**

2.1. Identifier *17*

2.2. Property *18*

2.3. Condition *21*

2.4. Ingredient *22*

2.5. Quantity *22*

2.6. Equipment *23*

2.7. Computation_forcefield *23*

2.8. Software_configuration *24*

2.9. Algorithm *24*

2.10. Parameter *25*

2.11. Citation *26*

3. CRIPT Supporting Nodes **27**

3.1. User Node *27*

3.2. Group Node *27*

3.3. File *28*

4. Graph examples **30**

4.1. Simple Chemical Reaction *30*

4.2. Diblock Copolymer Synthesis *31*

4.3. Reaction Kinetics *31*

4.4. Diblock Bottlebrush Synthesis *32*

4.5. Chemical Reaction with Aliquots *32*
4.6. Multi-Step Chemical Reaction (Library Generation) *33*
4.7. Material from Literature *33*
4.8. Characterization of Material *34*
4.9. Involved Material Characterization *34*
4.10. Block Copolymer Phase Behavior Annealing Study *34*
4.11. Extrusion and Characterization *35*
4.12. Computation to Produce Molecular Trajectories *35*
4.13. Computation to Simulate Reaction *36*
4.14. Computation to Generate Atomistic Forcefield *37*
4.15. Computation to Estimate Kinetic Rates *38*
4.16. Collaboration Across Collections *39*
4.17. Collaboration Across Projects (With Common Users) *40*
4.18. Collaboration Across Projects (Without Common Users) *41*
4.19. Access Control Within Projects *42*

5. Sub-Object Examples **43**
5.1. Material Node *43*
5.2. Process Node *49*

6. Controlled Vocabulary, Keys and Keywords **50**

7. Reference **51**

1. CRIPT NODES

The following sections break down CRIPT's (Community Resource for Innovation in Polymer Technology) data model and provide detailed discussions on all the nodes, sub-objects, and attributes. Nodes are the highest level in the data organization hierarchy and are the modular units used to construct the graph structure. Graph in this context refers to the graph data structure which consists of a set of vertices/nodes and edges (connection between nodes) with data being stored in nodes and relationships stored with edges. Fundamentally, nodes are an abstract blueprint of how data should be structured for a specific instance. For example, a *material* node provides a specific blueprint on how data describing water (a specific material instance) should be structured (boiling point, molecular mass, etc.). A key feature of these nodes is the presence of a globally unique auto-generated and persistent identifier, which for CRIPT is a URL (Uniform Resource Locator) as CRIPT is natively web-based. This unique identifier is the key piece of information that enables the graph structure to be built, as the presence of the unique identifier of one node in another node signifies an edge between those two nodes in the graph.

Sub-objects are groupings of attributes that are used to build nodes; this structuring is known as composition in object-oriented programming. They serve two purposes: first, they provide another hierarchy for organizing and grouping data. For example, the property sub-object links key, value, units, etc. together. On their own, each of these attributes are not conceptual distinct objects, but together make a concrete object. The second purpose of sub-objects is to provide multiple instances. For example, a material is likely to have multiple properties. Since a property is a sub-object, the structure can be reused to uniformly format each property instance.

The lowest level of the data model is attributes which are the individual pieces of information that are to be stored. Examples of an attribute include property key, property value, and unit of the value (e.g. 'boiling temperature: 100 °C' is decomposed into three attributes: property key: boiling temperature, property value: 100, unit: °C).

In the following sections, each node or sub-object will be discussed, and a table of attributes will be provided. Attribute tables include links between CRIPT nodes, required attributes, and attributes that have a controlled vocabulary. Links from one node to another can be identified in the tables by looking at the 'type' column, and the values in blue signify that it links to another node or sub-object. The attribute tables have a column dedicated to required attributes that must be provided to have a valid node/sub-object. Attributes that have an official CRIPT controlled vocabulary are denoted in the 'vocab' column. The other headers in the table are 'attribute' which contains the attribute names, 'type' referring to data type (e.g. int, float, list, str), examples, and description. Attributes with a 'list' type enable multiple objects to be added to that attribute and the attribute name is made plural. In the text of the following sections, nodes and sub-objects will be placed in *italics* and attributes will be placed in 'single quotes'. This notation will help differentiate between the colloquial use of the word and the same word showing up as a node in one context and an attribute in another.

As mentioned above, several attributes have a controlled vocabulary. The controlled vocabulary is introduced for these attributes to avoid the same key being entered multiple different ways (for example, 'boiling point' might be entered as 'boiling point', 'boiling temperature', 'BP', 'Bp', 'bP', 'bp') which would erode data interoperability. For more details on all the controlled

vocabularies see Section ‘Controlled Vocabulary, Keys and Keywords’ below. To avoid limiting users from entering only keys that are supported, CRIPT does allow user-defined vocabulary to be entered if it begins with a ‘+’. It is anticipated that on a regular basis, the controlled vocabulary will be expanded to include the most common user-defined properties, and the degenerate representations will be unified. The ‘+’-prefix ensures that the official namespace does not have conflicts with user-defined vocabulary when new keys are added to the controlled vocabulary.

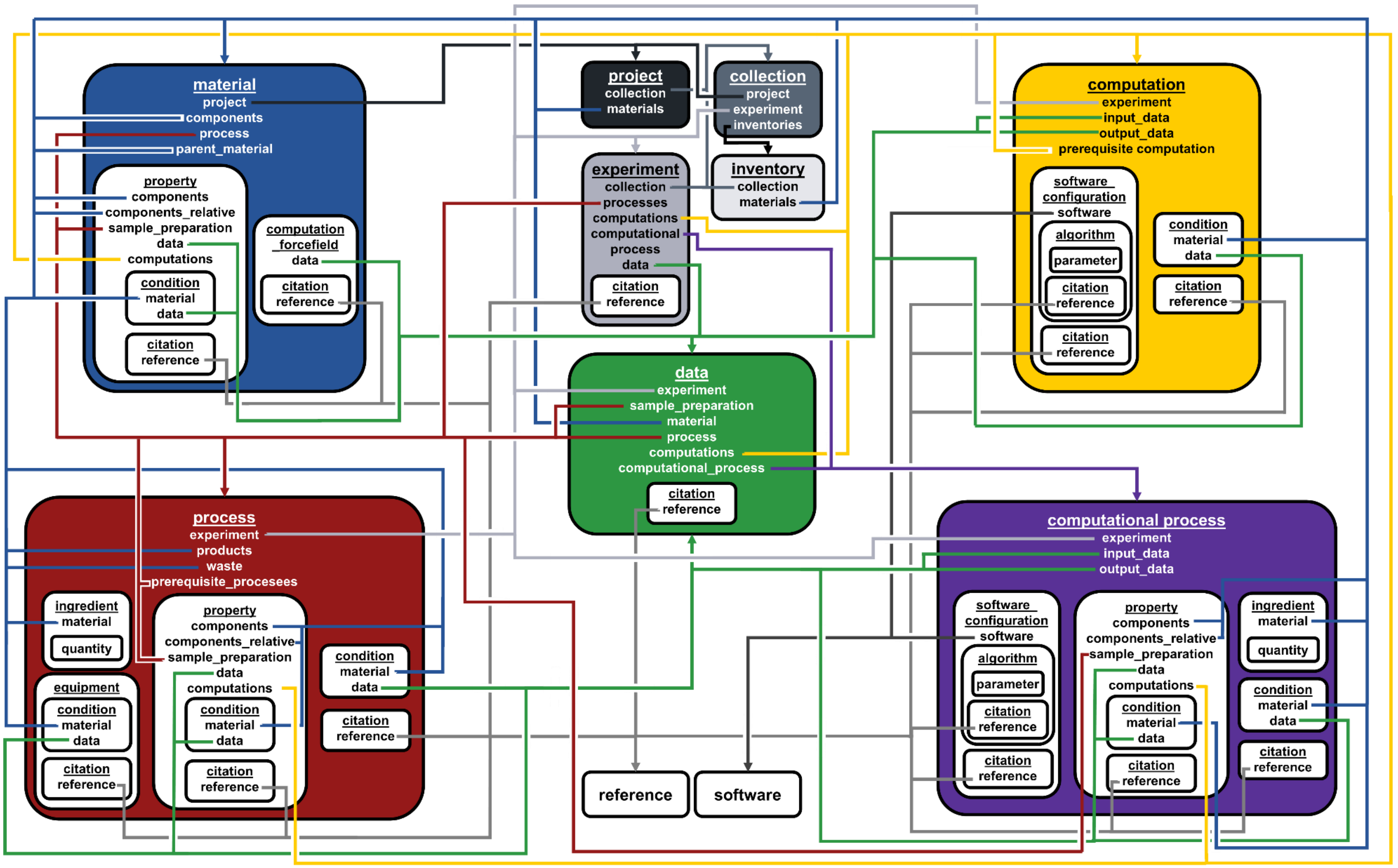

Figure S1: Overview of all possible connections between nodes and sub-objects in the CRIPT data model. Arrows start at the attribute that stores the persistent identifier (URL) for the arrow terminating node. Colored lines have no additional meaning and are only to help with visualization.

1.1. Base Attributes

Base attributes are common among all nodes. Nearly all base attributes are required, except for ‘notes’; however, ‘name’ is the only required attribute that the user will specify. The other attributes are all predefined or auto-filled metadata. The ‘group’ attribute provides the key link to the *group* node for access control. The ‘public’ attribute indicates whether the node is viewable by the public or only the members of the owning *group*. The ‘locked’ attribute defines whether a node is still editable and will be discussed further in the *collection* node. The ‘notes’ attribute is a miscellaneous information section which accommodates any information that is not explicitly supported by the data model. Ideally, the use of the ‘notes’ section will have a defined structure, like JavaScript Object Notation (JSON), which can make it findable and interpretable in the future or with custom codes. This is a key design feature that allows users to add custom data structures within CRIPT’s predefined data model, extending CRIPT for specific uses. The ‘node’ attribute refers to the type of node: *collection*, *material*, *process*, etc. The ‘model version’ serves to keep track of the data model version the document was encoded in. Recording this information in the node allows preforming updates to the data model.

Table S1: Base attributes.

attribute	type	example	description	required	vocab
url	str		unique ID of the node	True	
group	Group		group that owns the node	True	
public	bool	True	boolean indicating whether the node is publicly viewable	True	
locked	bool	True	boolean indicates whether the node is still editable	True	
name	str	“ATRP kinetic experiments”	descriptive label	True	
notes	str		miscellaneous information, or custom data structure		
node	str	user	type of CRIPT node	True	
model_version	str	0.0.1	version of data model	True	
updated_by	User		user that last updated the node	True	
created_by	User		user that originally created the node	True	
updated_at	datetime*	2022-02-03T06:14:22.610253Z	last date the node was modified (UTC time)	True	
created_at	datetime*	2022-01-04T10:13:52.325531Z	date it was created (UTC time)	True	
Abbreviations: Atom Transfer Radical Polymerization (ATRP), Universal Time Coordinated (UTC)					

* Datetime is formatted as International Organization for Standardization (ISO): yyyy-mm-ddThh:mm:ss.SSSZ string value. This applies for all future instances of datetime.

1.2. Project Node

The *project* node is a grouping of *collections* that embody the works of a research group, a research thrust, or work related to a specific area. Examples of a *project* are ‘sustainable polyester project’ or ‘self-assembly behavior of bottlebrush polymers’. The only required attribute is the ‘name’ (in the base attributes), and the ‘name’ needs to be unique within the database. A *project* node links

to one or more *collections* and all the *material* nodes used in the project. *Material* nodes are linked to the *project* to address issues with uniqueness and the reuse of *material* nodes. *Material* nodes hypothetically could be defined globally in the database and not tied to a *project*. However, if a user referenced a *material* node from another *project* and then it was deleted, then there would be a broken *material* node reference leading to the loss of data integrity. Thus, to provide protection without having every use of a *material* node needing to be unique, nodes are defined within a *project*. This means if a *material* from another *project* or from the public repository wants to be used/referenced in another *project*, it will have to be copied into the *project* (a link to the original node is stored for provenance).

Table S2: Attribute table for the *project* node.

attribute	type	example	description	required	vocab
'base' attributes (see details above)					
collections	list[Collection]		collections that relate to the project		
materials	list[Materials]		materials owned by the project		

1.3. Collection Node

The *collection* node is a grouping of experiments or simulations. It serves as an organizational tool similar to a folder on a computer desktop, a lab notebook, or a grouping of experiments that make up a publication. The only required attribute is the 'name' (in the base attributes), and the 'name' needs to be unique within the parent *project*. A *collection* node links to one or more *experiments* and can contain one or more *inventory* nodes. One key feature of the *collection* node is the ability for a *collection* to be published with a DOI (Digital Object Identifier), 'cript_doi', allowing data to be published along with manuscripts or otherwise disseminated in archival form. This DOI serves as a permanent link that can be added to a manuscript to enable readers of the manuscript to rapidly gain access to the data that was produced in the manuscript. Once the manuscript is published, a *reference* node can be created to link the collection back to the manuscript. Upon generation of the 'cript_doi' nodes will no longer be editable by setting 'locked' attribute in base attributes to 'True'.

Table S3: Attribute table for the *collection* node.

attribute	type	example	description	required	vocab
'base' attributes (see details above)					
project	Project		project the collection is associated with		
experiments	list[Experiment]		experiments that relate to the collection		
inventories	list[Inventory]		inventory owned by the collection		
cript_doi	str	10.1038/1781168a0	DOI: digital object identifier for a published collection; CRIPT generated DOI		
citations	list[Citation]		reference to a book, paper, or scholarly work		

1.4. Experiment Node

The *experiment* node is the grouping of nodes for an experiment which includes *processes*, *computations*, *computational_processes*, and *data* nodes. In the simplest case, where an experiment just involves characterization of a single material, the *experiment* node would only link to *data* nodes. In a more complex case, multi-step processes can be grouped within a single *experiment* node (e.g., multiblock copolymer synthesis). Users are free to structure *experiment* nodes to best suit their research, including choosing the degree of granularization of experiments. Whether a user decides to break an experiment into multiple *experiment* nodes or group all the data into single *experiment* node does not change how the other nodes will be represented in the data model. The required attribute for the *experiment* node is only the 'name' (in the base attributes) which needs to be unique within the parent *collection*.

Table S4: Attribute table for the *experiment* node.

attribute	type	example	description	required	vocab
'base' attributes (see details above)					
collection	Collection		collection associated with the experiment	True	
processes	list[Process]		process nodes associated with this experiment		
computations	list[Computation]		computation method nodes associated with this experiment		
computational_ processes	list[Computational Process]		computation process nodes associated with this experiment		
data	list[Data]		data nodes associated with this experiment		
funding	list[str]	['OIA-2134795']	funding source for experiment		
citations	list[Citation]		reference to a book, paper, or scholarly work		

1.5. Inventory Node

The *inventory* node is a list of *material* nodes. It serves as an organizational tool for rapidly finding *material* nodes that pertain to a *collection*. An example of an *inventory* can be a grouping of materials that were extracted from literature and curated into a group for machine learning, or it can be a subset of chemicals that are used for a certain type of synthesis, such as 'ATRP monomers' or 'RAFT chain transfer agents' (reversible addition fragmentation chain transfer polymerization (RAFT)). The required attribute is the 'name' (in the base attributes) which must be unique within a *collection*.

Table S5: Attribute table for the *inventory* node.

attribute	type	example	description	required	vocab
'base' attributes (see details above)					
collection	Collection		collection associated with the inventory	True	
materials	list[Material]		materials that you like to group together		

1.6. Material Node

The *material* node is a collection of the identifiers and properties of a chemical, mixture, or substance. Examples of materials include water, brine (water + NaCl), polystyrene, polyethylene glycol hydrogels, vulcanized polyisoprene, mcherry (protein), and mica. The required attribute is the ‘identifiers’. The ‘identifiers’ are unique labels or descriptive information of a material such as preferred name, abbreviations, SMILES[1] (simplified molecular-input line-entry system), BigSMILES[2], chemical formula, etc. and will be discussed further in the *identifier* section. Mixtures can be represented by linking to pure chemicals (*material* nodes) with the ‘components’ attribute, and the ratio of components can be specified in ‘properties’. *Properties* are qualities, traits, or characteristics of a material, such as molecular mass, composition ratios, boiling temperature, solubility, elastic modulus, or color. Note that all *properties* must be simultaneously valid, meaning that multiple measurements of composition, or molecular mass are allowed, but should provide information about a single material in a single state. *Properties* are also used to associate data, structural information (such as NMR (nuclear magnetic resonance), or IR (infrared) spectrum), or data that has yet to be analyzed to the *material* node. The ‘process’ attribute provides a link to the *process* node that produced the material. Since *materials* live within a single *project*, it is expected that they may need to be copied from one *project* to another; the ‘parent_material’ attribute provides a link to the original *material* node. The ‘keywords’ attribute allows users to specify words that classify the material, which seeks to make the *material* node more findable. CRIPT has a list of supported keywords for monomer classification by chemical type, as well as polymer classification by chemical type and architecture (see ‘Controlled Vocabulary, Keys and Keywords’ for lists). If the *material* was generated through a computation, the ‘computation_forcefield’ is required and provides a place to specify building blocks, mappings, solvation, and force field attributes. If the *material* was generated by extracting data from a literature source, each *property* can define the source of the information.

Table S6: Attribute table for the *material* node.

attribute	type	example	description	required	vocab
‘base’ attributes (see details above)					
project	Project		project the collection is associated with		
identifiers	list[Identifier]		material identifiers	True	
components	list[Material]		list of components that make up the mixture		
properties	list[Property]		material properties		
process	Process		process node that made this material		
parent_material	Material		material node that this node was copied from		
computation_ forcefield	Computation Forcefield		computation forcefield	Cond*	
keywords	list[str]	[thermoplastic, homopolymer, linear, polyolefins]	words that classify the material		True

* Conditional: Required for computational materials.

1.7. Process Node

The *process* node contains a list of ingredients, quantities, and procedure information for an experimental material transformation (chemical and physical). Examples of a *process* include chemical reactions, separations, and extrusions. The required attributes are the 'name' (in the base attributes) and 'type'. The *process* node was designed to be used in one of two ways: the first approach allows users to enter an entire process in a single *process* node, and the second approach allows for a series of *process* nodes to be chained together with each node constituting a single step in a larger process. This use of a single *process* node is akin to a procedure paragraph typically found in a methods section of a journal article. For the second approach, where each *process* node is a step in the process, the 'description' attribute is an explanation of that single step, and the 'type' for that step can be more accurately defined from the available keywords (e.g., mixing, extraction, precipitation, drying). Chaining *process* nodes is done through the 'prerequisite_processes' attribute which supports multiple prerequisite processes in the case of converging processes. Note that 'prerequisite_processes' should only capture immediate prerequisites. For diverging processes, an intermediate *material* node will be required as only linear chains of *process* nodes are supported.

The 'ingredients' attribute links to *material* nodes and specifies the associated quantities (mass, volume, pressure, and moles) for each material used in the process. Relative quantities like equivalence, molarity, mass fraction, etc. are not stored as they can be computed from the absolute quantities. The choice to store absolute quantities was to ensure scale of the reaction was recorded and relative quantities can be calculated from absolute quantities if sufficient details are provided. If the *process* leads to the creation of new *materials*, they can be linked in the 'product' attribute. *Materials* that are discarded in the *process* can be specified with the 'waste' attribute. 'Conditions' are environmental variables or set process variables of the *process*. Examples of 'conditions' are duration, temperature, pressure, and stirring rate. 'Equipment' are physical instruments, tools, glassware, etc. used in a process. The *equipment* sub-object also has a 'condition' attribute. With two locations to specify conditions, the 'conditions' attribute in the *process* node is to be used for global conditions, while the 'conditions' attribute in the *equipment* is to specify local conditions, or conditions controlled by that equipment. The 'properties' sub-objects are emergent traits that characterize the process and the process results, which includes things like reaction yield, rate constants, or activation energies. The 'keywords' attribute allows users to specify words that classify the process to make it more findable. Currently supported keywords include chemical and physical transformations (see 'Controlled Vocabulary, Keys and Keywords' for lists).

Table S7: Attribute table for the *process* node.

attribute	type	example	description	required	vocab
'base' attributes (see details above)					
experiment	Experiment		experiment the process belongs to		
type	str	mix	type of process	True	True
ingredients	list[Ingredient]		ingredients		
description	str	To oven-dried 20 mL glass vial, 5 mL of styrene and 10 ml of toluene was added.	explanation of the process		
equipment	list[Equipment]		equipment used in the process		
products	list[Material]		desired material produced from the process		
waste	list[Material]		material sent to waste		
prerequisite_ processes	list[Process]		processes that must be completed prior to the start of this process		
conditions	list[Condition]		global process conditions		
properties	list[Property]		process properties		
keywords	list[str]		words that classify the process		True
citations	list[Citation]		reference to a book, paper, or scholarly work		

1.8. Data Node

The *data* node contains the meta-data to describe data that is beyond a single value, *i.e.* n-dimensional data. Common examples of data nodes for experimental research are NMR spectra, stress-strain curves, equipment sensor data, and timeseries. Examples for computational research would include system trajectories, online logged observables, or complete snapshots. The required attributes are the 'name' (in the base attributes), 'type', and 'files'. The 'type' attribute specifies what the data is (^{1}H NMR, SEC (size exclusion chromatograph), stress-strain curve, etc.). The 'file' attribute links to file objects which contain meta-data specific to the file, like file type (csv, txt, jpg), as well as the link to the raw file. Multiple files can be attached to a single *data* node if the data is spread over several files. Data is not directly stored in the *data* node because this would significantly slow down searching speeds and dramatically increase the memory burden of the database. The solution is to store the data in lower cost storage databases, and only store the link to the data in the CRIPT database. 'sample_preperation' is included to capture the processing done to prepare the sample for analysis, the equipment used to collect the data, calibration files, etc.

Table S8: Attribute table for the *data* node.

attribute	type	example	description	required	vocab
'base' attributes (see details above)					
experiment	Experiment		experiment the data belongs to		
type	str	nmr_h1	data type keyword	True	True
files	list[File]		list of file nodes	True	
sample_preperation	Process		sample preparation		
computations	list[Computation]		data was produced from this computation method		
computational_ process	Computational_ process		data was produced from this computation process		
materials	list[Material]		materials with attributes associated with the data node		
processes	list[Process]		processes with attributes associated with the data node		
citations	list[Citation]		reference to a book, paper, or scholarly work		

1.9. Computation Node

The *computation* node describes the transformation of data or the creation of a computational data set. Common *computations* for simulations are energy minimization, annealing, quenching, or NPT/NVT (isothermal-isobaric/canonical ensemble) simulations. Common *computations* for experimental data include fitting a reaction model to kinetic data to determine rate constants, extracting a plateau modulus from a time-temperature-superposition, or calculating radius of gyration with the Debye function from small angle scattering data. The required attributes are 'name' and 'type'; which 'type' specifies the category of computation performed. The 'input_data' attribute specifies the *data* that is being transformed by the *computation*, which is optional for initialization of a simulation. The 'software_configuration' attribute defines the software, version, and algorithm used in the computation. Note that multiple software may be defined for a single *computation*. For *computations* of a typical simulation, *conditions* are the set of variables that define the thermostatic state of a system, such as temperature, energy, number of atoms/molecules, and pressure. *Computations* can also be chained together without an intermediate data via the 'prerequisite_computations' attribute. Enhanced sampling techniques that combine multiple simulation runs into a single, overarching result, like a free-energy profile of umbrella sampling, can be described with each simulation run as a parallel arranged set of individual *computations*.

Table S9: Attribute table for the *computation* node.

attribute	type	example	description	required	vocab
'base' attributes (see details above)					
experiment	Experiment		experiment the computation belongs to		
type	str	general molecular dynamics simulation	category of computation	True	True
input_data	list[Data]		input data nodes		
output_data	list[Data]		output data nodes		
software_ configurations	list[Software Configuration]		software and algorithms used		
condition	list[Condition]		setup information		
prerequisite_ _computation	Computation		prior computation method in chain		
citations	list[Citation]		reference to a book, paper, or scholarly work		
notes	str		additional description of the step		

1.10. Computational_process Node

A *computational_process* is a simulation that processes or changes a virtual *material*. Examples include simulations of chemical reactions, chain scission, cross-linking, strong shear, *etc.* A computational process may also encapsulate any computation that dramatically changes the materials properties, molecular topology, and physical aspects like molecular orientation, etc. The *computation_forcefield* of a simulation is associated with a *material*. As a consequence, if the *forcefield* changes or gets refined via a computational procedure (density functional theory, iterative Boltzmann inversion for coarse-graining etc.) this *forcefield* changing step must be described as a *computational_process* and a new *material* node with a different *computation_forcefield* needs to be created.

The *computational_process* node requires both 'ingredients' (i.e., at least one *material* to be processed) and 'input' *data* (i.e., the configuration of the material under processing) as input. Besides input *data* and *material* nodes, other required attributes are 'name' and 'type'; which 'type' specifies the category of computation performed. Similar to the *computation* node, the 'software_configuration' and the 'condition' attribute defines a set of software (with version and algorithm) and set thermostatic variables of a system, respectively. In contrast to *computation*, *computational_process* nodes cannot be chained together given they require both *material* and *data* as input but only have *data* as output.

Table S10: Attribute table for the *computational_process* node.

attribute	type	example	description	required	vocab
'base' attributes (see details above)					
experiment	Experiment		experiment the computational_process belongs to		
type	str	general molecular dynamics simulation	category of computation	True	True
input_data	list[Data]		input data nodes	True	
output_data	list[Data]		output data nodes		
ingredients	list[Ingredient]		ingredients	True	
software_ configurations	list[Software Configuration]		software and algorithms used		
condition	list[Condition]		setup information		
properties	list[Property]		computation process properties		
citations	list[Citation]		reference to a book, paper, or scholarly work		
notes	str		additional description of the step		

1.11. Reference Node

The *reference* node contains the metadata for a literature publication, book, or anything external to CRIPT. The *reference* node does not contain the base attributes and is meant to always be public and static to allow globally link data to the *reference*. The *reference* node is always used inside the *citation* sub-object to enable users to specify the context of the reference (see *citation* sub-object for more details). The required attributes are the 'title' and 'type'. Unique identifiers like DOI (digital object identifier) are highly recommended to be recorded.

Table S11: Attribute table for the *reference* node.

attribute	type	example	description	required	vocab
url	str		CRIPT's unique ID of the node	True	
type	str	journal_article	type of literature	True	True
title	str	'Living' Polymers	title of publication	True	
authors	list[str]	Michael Szwarc	list of authors		
journal	str	Nature	journal of the publication		
publisher	str	Springer	publisher of publication		
year	int	1956	year of publication		
volume	int	178	volume of publication		
issue	int	0	issue of publication		
pages	list[int]	[1168, 1169]	page range of publication		
doi	str	10.1038/1781168a0	DOI: digital object identifier	Cond.*	
issn	str	1476-4687	ISSN: international standard serial number	Cond.*	
arxiv_id	str	1501	arXiv identifier		
pmid	int	########	PMID: PubMed ID		
website	str	https://www.nature.com/articles/1781168a0	website where the publication can be accessed		

* Conditional: Required depending on the 'type'. Example: journal articles require DOI.

1.12. Software Node

The software node contains metadata for a computation tool, code, programing language, or software package. Similar to the *reference* node, the *software* node does not contain the base attributes and is meant to always be public and static. The required attributes are ‘name’ and ‘version’, while ‘source’ is optional. For in-house, unpublished code ‘version’ can be filled with a version control handle like a git-hash or a text like “unpublished”.

Table S12: Attribute table for the *software* node.

attribute	type	example	description	required	vocab
url	str		CRIPT’s unique ID of the node	True	
name	str	LAMMPS	type of literature	True	
version	str	23Jun22	software version	True	
source	str	lammps.org	source of software		
Abbreviations: Large-scale Atomic/Molecular Massively Parallel Simulator (LAMMPS)					

2. SUB-OBJECTS

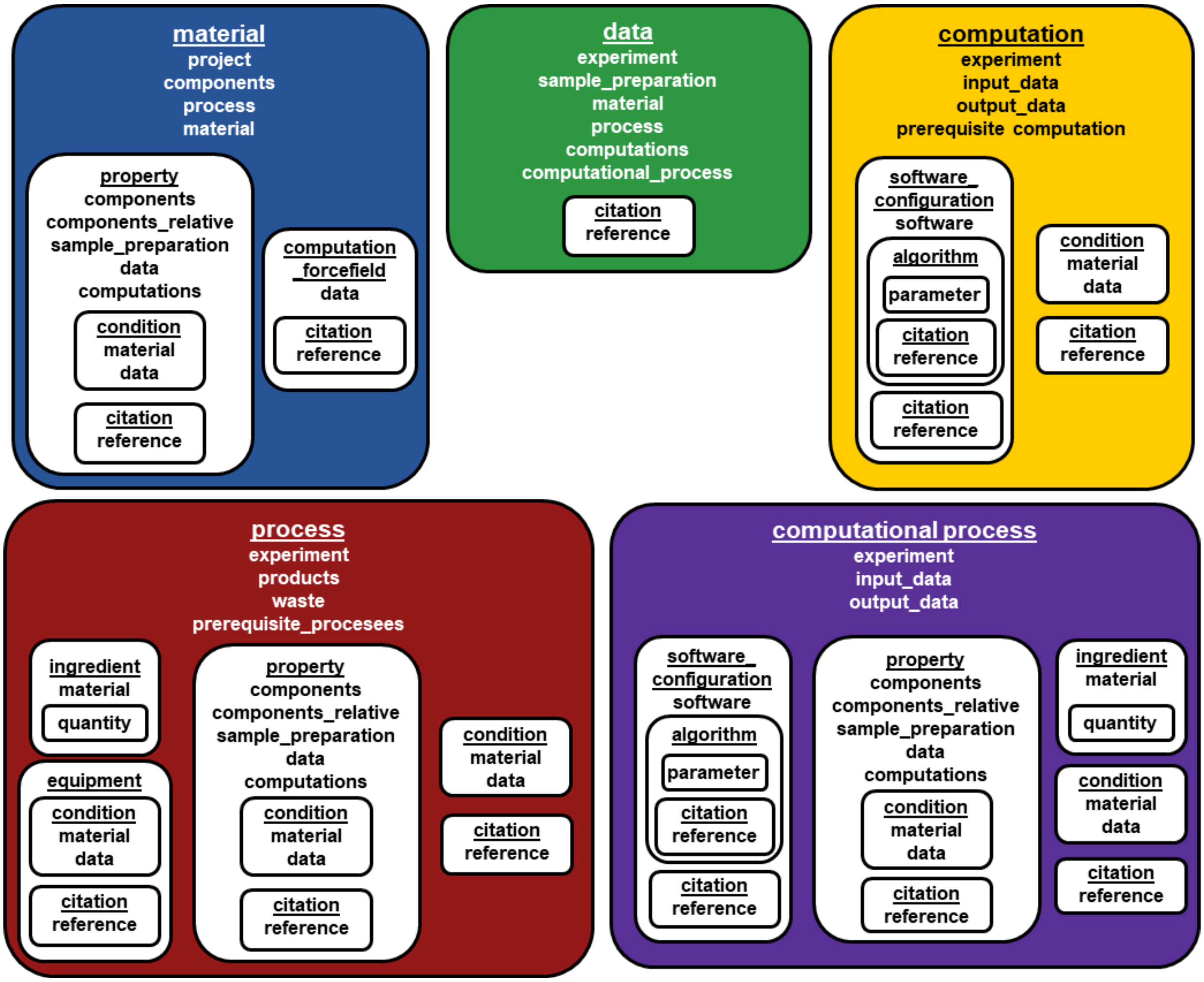

Figure S2: Depiction of each core node and the sub-objects that are contained within.

2.1. Identifier

Identifiers are a sub-object for the *material* node and are unique labels or descriptive information for a material. Examples of *identifiers* include preferred name, alternative names, SMILES[1], BigSMILES[2], chemical formula, chemical formula of repeat unit, PubChem cid[3] (compound ID number (cid)), and InChI[4] (International Chemical Identifier). Users are encouraged to provide unambiguous identifiers whenever possible to ensure that data is appropriately attributed to specific materials. *Identifiers* are a key-value pair, with 'key' specifying the type of identifier and 'value' being the content.

Table S13: Attributes table for the *identifier* sub-object.

attribute	type	example	description	required	vocab
key	str	names	key for identifier	True	True
value	Any	['styrene', 'ethenylbenzene', 'vinylbenzene']	value	True	

2.2. Property

Properties are qualities or traits of a material or process. They are part of the *material* node (e.g., melting temperature, molecular mass), *process* node (e.g., conversion, rate constants) and *computational_process* node (e.g., total energy, bond length, hydration number). The required attributes are the 'key' and 'type'. The 'key' attribute specifies what the property is (e.g., melting temperature). The 'type' attribute specifies what the meaning of the value is, such as 'value' which represents a single point measurement, or 'min', 'max', 'mean' can be specified for multipoint measurements (more discussion about this below). The 'value' and 'unit' attributes are conditionally required, meaning that they are required depending on the 'key' attribute. 'Uncertainty' must use the same units as the 'value' attribute, if applicable. The 'conditions' attribute allows the user to specify any environmental variables or system descriptors that are relevant to the property. The 'method' attribute specifies the analytical instrument or computational origins of the *property*. 'Sample_preperation' can be used to provide a more detailed definition of the method, instrument (in the 'equipment' attribute in the process), and process for measuring the *property*. The *property* sub-object provides links to the *data* node or *computational* node that produced the value to provide provenance of the *property*. If multiple datasets are needed to obtain a *property*, a *computation* node can be used which defines how the multiple datasets combine to yield the 'value'. The 'citation' attribute provides a link to the source of the property if it came from a literature reference. The 'notes' attribute is present to capture other data not explicitly captured by the data model. One common use of 'notes' for *properties* is to place the definition for any custom *property* 'keys'.

To specify more precisely the chemical or molecular structure to which the property refers to, the 'components', 'components_relative', and 'structure' attributes can be used. The 'components' attribute allows the user to specify the *material* nodes of a mixture to which a property refers to. The common use of 'components' is for specifying the composition or ratio between components in a mixture. In the case of a ratio, one or more materials can be specified in 'components' for the left side of the ratio, one or more materials can be specified in 'components_relative' for the right side of the ratio, and the ratio is specified in value. The 'structure' attribute provides an orthogonal method to specify what chemical structural feature the properties are attributed to by specifying atom mappings in the SMILES and BigSMILES string. Atom mapping is done within the SMILES and BigSMILES strings with brackets and an integer index separated by a colon, [element: index]. For absolute quantities index 1 should be used. For ratio or relative based properties, index: 1 corresponds to the molecular fragment that the property is 'about', and index 2 is the molecular fragment that the property is 'relative to'.

Mathematical functions can be defined as values using latex notation. Definitions should follow the following format: '$$equation$$'. Example of a rate law:

latex	`$$\dfrac{d[styrene]}{dt} = 0.4 [THF]^{0.8} [styrene]^1 [SecBuLi]^{1.1}$$`
result	$\dfrac{d[styrene]}{dt} = 0.4\ [THF]^{0.8}\ [styrene]^{1}\ [SecBuLi]^{1.1}$

Table S14: Attribute table for the *property* sub-object.

attribute	type	example	description	required	vocab
key	str	modulus_shear	type of property	True	True
type	str	min	type of value stored	True	True
value	Any	1.23	value or quantity	Cond*	
unit	str	gram	unit for value	Cond*	
uncertainty	Any	0.1	uncertainty of value		
uncertainty_ type	str	standard_deviation	type of uncertainty		True
components	list[Material]		material that the property relates to**		
components_ relative	list[Material]		material that the property relative to**		
structure	str	{[][$][C:1][C:1][$], [$][C:2][C:2]([C:2]) [$][]}	specific chemical structure associate with the property with atom mappings**		
method	str	sec	approach or source of property data		True
sample_prep aration	Process		sample preparation		
conditions	list[Condition]		conditions under which the property was measured		
data	Data		data node		
computations	list[Computation]		computation method that produced property		
citations	list[Citation]		reference to a book, paper, or scholarly work		
notes	str		miscellaneous information, or custom data structure (e.g.; JSON)		

* Conditional: most properties will require a 'value' and 'unit'; however, there are a few properties like 'associated' and 'structure' which are used only to link to data nodes and do not have a 'value' or 'unit'.

** Components and atom mapping examples:

To specify the ratio of the two monomers in a random co-polymer with atom mapping the [atom:1] will be applied to the first monomer, and [atom:2] will be applied to the second monomer.

```
{
  "key": "conc_molar_ratio",
  "type": "value",
```

```
  "value": 0.20,
  "unit": "",
  "structure": "{[][$][C:1][C:1][$],[$][C:2][C:2]([C:2])[$][]}"
}
```

This should be interpreted as a ratio of 0.20 [$]CC[$] : 1 [$]CC(C)[$].

To specify a mixture of a small molecule and polymer with atom mapping the [atom:1] will be applied to the small molecule, and [atom:2] will be applied to the polymer.

```
{
  "key": "conc_molar_ratio",
  "type": "value",
  "value": 0.20,
  "unit": "",
  "structure": "[O:1][C:1](=[O:1])[C:1]([C:1])[O:1].{[][>][C:2](=[O:2])[C:2]([C:2])[O:2][<][]}",
}
```

This should be interpreted as a ratio of 0.20 OC(=O)C(C)O : 1 {[][>]C(=O)C(C)O[<][]}.

To specify the ratio of a small molecule and polymer in a mixture, the ‘components’ and ‘components_relative’ attributes can be used. First specify the small molecule (*material* node) in ‘components’ and then specify the polymer in ‘conditions’. This is specifying the molar ratio (0.2 lactic acid) : (1 poly(lactic acid)).

```
{
  "key": "conc_molar_ratio",
  "type": "value",
  "value": 0.20,
  "unit": "",
  "components": ["URL for lactic acid material node"],
  "components_relative”: [“URL for poly(lactic acid) material node”]
}
```

Mixture of a block co-polymer and homo-polymer from the first block, specifying the ratio of the two monomers.

```
{
  "key": "conc_molar_ratio",
  "type": "value",
  "value": 0.20,
  "unit": "",
  "structure": "{[][$][C:1][C:1][$][]}.{[][$][C:1][C:1][$][>]}{[<][$][C:2][C:2]([C:2])[$][]}",
}
```

This should be interpreted as a ratio of 0.20 [$]CC[$] : 1 [$]CC(C)[$].

2.3. Condition

Conditions are environmental variables, equipment settings, states of the system, or influencing factors. Examples of *conditions* for physical experiments include temperature, pressure, stirring rate, and for computations *conditions* include box dimensions, integration time step, ensemble, thermostat parameters, *etc. Conditions* are found in the *process, computation* and *computational_process* nodes and in the *property* and *equipment* sub-objects. The required attributes for *conditions* are the ‘key’ which specifies what type of condition it is, ‘type’ which is the information you want to specify (such as: ‘value’, ‘min’, ‘max’, ‘mean’), and ‘value’. The ‘material’ attribute is used when the value needs to link to a *material* node, for example when specifying the atmosphere being nitrogen. The *condition* sub-object provides links to the *data* node to allow users to attach the associated raw data. For *computation* and *computational_process* nodes the condition should specify the complete statistical ensemble, e.g., for a simulation in the grand-canonical ensemble (μVT) the chemical potential μ, simulation volume V, and temperature T should be specified as a *condition*. The realization details of the ensemble (e.g., thermostat) for the simulation can be specified via the *algorithm* attributes in *computation* nodes.

The ‘measurement_id’ and ‘set_id’ attributes provide the ability to link together *conditions* that are related. The ‘measurement_id is used to link *conditions* that are descriptive of the same state. For example, the measurement of temperature and pressure of a system at a single time point can be linked with the same ‘measurement_id’. The ‘set_id’ links together *conditions* across a set or series, with all the points belonging to a specific series all having the same index. A common example is a timeseries where each point in the timeseries would have the same index. The writing of individual time points directly into the database with this mechanism is recommended for under ten datapoints, after which the series should be moved into the *data* node and min, max and mean values can be reported and grouped with the same ‘set_id’. This minimizes the size of the data stored directly in the database, while keeping key features of the series available for searching and quick referencing.

Table S15: Attribute table for the *condition* object.

attribute	type	example	description	required	vocab
key	str	temp	type of condition	True	True
type	str	min	type of value stored, 'value' is just the number, 'min', 'max', 'avg', etc. for series	True	True
descriptor	str	upper temperature probe	freeform description for condition		
value	Any	1.23	value or quantity	True	
unit	str	gram	unit for value		
uncertainty	Any	0.1	uncertainty of value		
uncertainty_type	str	std	type of uncertainty		True
material	list[Material]		material that the condition relates to		
set_id	int	0	ID of set (used to link measurements in as series)		
measurement_id	int	0	ID for a single measurement (used to link multiple condition at a single instance)		
data	Data		detailed data associated with the condition		

2.4. Ingredient

Ingredients are links to *material* nodes with the associated quantities. *Ingredients* are found both in the *process* and *computational_process* node. The required attributes are the 'material' reference and at least one of the 'quantities' (mass, volume, pressure, mole) must be defined.

Table S16: Attribute table for the *ingredient* object.

attribute	type	example	description	required	vocab
material	Material		material	True	
quantities	list[Quantity]		quantities	True	
keyword	str	catalyst	keyword for ingredient		True

2.5. Quantity

Quantities are the amount of material involved in a process. They are used in the *ingredients* sub-object. The required attributes are 'key', 'value' and 'unit'.

Table S17: Attribute table for the *quantity* object.

attribute	type	example	description	required	vocab
key	str	mass	type of quantity	True	True
value	Any	1.23	amount of material	True	
unit	str	gram	unit for quantity	True	
uncertainty	Any	0.1	uncertainty of value		
uncertainty_type	str	std	type of uncertainty		True

2.6. Equipment

Equipment is physical instruments, tools, glassware, etc. used in a process. Settings, or environmental variables controlled by the equipment can be specified with the 'conditions' attribute. Equipment specification, configuration, and calibration files can be attached to the equipment sub-object with the use of a *File*.

Table S18: Attribute table for the *equipment* object.

attribute	type	example	description	required	vocab
key	str	hot plate	material	True	True
description	str	Hot plate with silicon oil bath with stir bar	additional details about the equipment		
conditions	list[Condition]		conditions under which the property was measured		
files	list[File]		list of file nodes to link to calibration or equipment specification documents		
citations	list[Citation]		reference to a book, paper, or scholarly work		

2.7. Computation_forcefield

Computational materials require additional information to define the material's identity. Specifically, this includes the solvation (implicit or explicit), the forcefield (to describe the interactions or dynamics) and the building blocks that are used to relate the chemistry to the forcefield. For example, polystyrene simulated using different force fields, such as CHARMM (chemistry at Harvard macromolecular mechanics), GROMOS (Groningen molecular simulation), and MARTINI (a general-purpose coarse-grained force field by University of Groningen) or Dry-MARTINI will yield different properties.

If the simulations are performed with a standard forcefield, like OPLS-AA (optimized potentials for liquid simulations- all atom model), CHARMM, or GROMOS, this can be specified with the 'key'. Users are highly encouraged to specify the source of the standard forcefield in 'source'. For example, when using the GROMOS forcefield shipped with a GROMACS (Groningen machine for chemical simulations) installation, specify the GROMACS installation and version number to improve reproducibility. For modified or non-standard forcefields, it is highly recommended to include a detailed description in 'description' and upload the exact input files that describe the forcefield and its parameters with the *data*. For example, obtaining OPLS-AA parameters via the LigParGen tool produces an forcefield input file, which should be attached as a *data node*.

The 'key_building_block' attribute specifies the level of description the simulation uses, for example all-atom simulations or united atom descriptions. Explicitly setting this attribute allows for fast searching of similar entries in the databank. The 'coarse_grained_mapping' attribute can be used to detail how chemical structures are mapped onto the simulation object like beads i.e., MARTINI mapping. This becomes especially important if the simulation describes the *material* with a coarse-grained model where multiple atoms are combined into interaction beads. The 'implicit_solvent' description is explicitly listed for searchability. If an explicit solvent is used, it should be included as a *material* with corresponding *computation_forcefield*. Otherwise, the use

of implicit solvents needs to be specified in the 'implicit_solvent' attribute. Previously published forcefields should be referenced via the 'citation' attribute.

Table S19: Attribute table for the *computation_forcefield* sub-object.

attribute	type	example	description	required	vocab
key	str	CHARMM27	type of forcefield	True	True
building _block	str	atom	type of building block	True	True
coarse_grained _mapping	str	SC3 beads in MARTINI forcefield	atom to beads mapping		
implicit_solvent	str	water	Name of implicit solvent		
source	str	package in GROMACS	source of forcefield		
description	str	OPLS forcefield with partial charges calculated via the LBCC algorithm	description of the forcefield and any modifications that have been added		
data	Data		details of mapping schema and forcefield parameters		
citation	list[Citation]		reference to a book, paper, or scholarly work		
Abbreviations: special apolar bead to model rings (SC3), local bond - charge correction (LBCC)					

2.8. Software_configuration

The *software_configuration* sub-object includes software and the set of algorithms to execute *computation* or *computational_process*. The required attribute is 'software'. The 'algorithm' attribute can be used to specify specifically what methods and parameters are used to set up the software.

Table S20: Attribute table for the *software_configuration* sub-object.

keys	type	example	description	required	vocab
software	Software		software used	True	
algorithms	list[Algorithm]		algorithms used		
notes	str		miscellaneous information, or custom data structure (e.g.; JSON)		
citation	list[Citation]		reference to a book, paper, or scholarly work		
Abbreviations: JavaScript Object Notation (JSON)					

2.9. Algorithm

Algorithm consists of parameter used in the *computation* and *computational_process*. This includes, but is not limited to:

1) algorithms of cell_initate 'key' to pack coils of polymer and other ingredient to simulation box with 'types' like amorphous cell module in Material Studio, RIS (rotational isomeric state model), SARW (self-avoiding random walk) on lattice;
2) algorithms of 'thermostat' or 'barostat' key to maintain constant temperature or pressure with 'type' like Nose-Hoover, Berendsen, Lagevin, Anderson;
3) algorithms of 'neighbor_list' key to define/update localized domain of building blocks with 'type' like Verlet or linked list;

4) algorithms of ‘integration’ key to integrate the motion of building blocks and their non-bonded interactions with ‘type’ like Leap-frog scheme of Verlet, particle mesh Ewald sum;
5) algorithms of ‘bond_constraint’ key to constrain the fast-vibrating bonds, usually for bonds with hydrogen atoms with ‘type’ like SHAKE (lagrange multiplier-based algorithm for shaking bond constraints between rigid molecules), RATTLE (algorithm extended from SHAKE via velocity integration).

To specify the ‘type’ of thermos_barostat, the ‘key’ (e.g., Nose-Hoover) and the associated *parameter* (i.e., damping_time) can be listed. For user-defined ‘key’ and ‘type’, links to the corresponding *data* node are also available. A full list of supported attributes for *setup* with the associated descriptions are given below.

Table S21: Attribute table for the *algorithm* sub-object.

keys	type	example	description	required	vocab
key	str	ensemble, thermo-barostat	system configuration, algorithms used in a computation	True	True
type	str	NPT for ensemble, Nose-Hoover for thermostat	specific type of configuration, algorithm	True	
parameter	list[Parameter]		setup associated parameters		
citation	list[Citation]		reference to a book, paper, or scholarly work		
Abbreviations: isothermal-isobaric (NPT)					

2.10. Parameter

A *parameter* is an input value to an algorithm. For typical computations, the difference between *parameter* and *condition* lies in whether it changes the thermodynamic state of the simulated system: Variables that are part of defining a thermodynamic state should be defined as a *condition* in a parent node. Therefore, ‘number’ and ‘volume’ need to be listed as *conditions* while ‘boundaries’ and ‘origin’ are *parameters* of ensemble size. Note that ‘number’ represents the count of ‘building_block’ (attribute in *computation_forcefield*) in the ensemble. ‘Origin’ and ‘boundaries’ are defined with ‘keys’ of Cartesian, cylindrical, spherical, etc.: The ‘value’ of origin is a three-number list [o1,o2,o3]. While for boundaries, its ‘value’ is a nine-number list [b11,b12,b13, b21,b22,b23, b31,b32,b33] containing three vectors, b11,b12,b13 and b21,b22,b23 as well as b31,b32,b33, each define the plane of a boundary.

Table S22: Attributes table for the *parameter* sub-object.

attribute	type	example	description	required	vocab
key	str		key for identifier	True	True
value	Any		value	True	
unit	str		unit for parameter		

2.11. Citation

The *citation* sub-object provides a link to papers, books, or other scholarly work and allows users to specify in what way the work relates to that data. More specifically, users can specify that the data was directly extracted from, inspired by, derived from, etc. the *reference*. Citations can be found in the *experiments*, *process*, *data*, *computation*, *computational_process* nodes and in the *property*, *computation_forcefield*, *equipment*, *software_configuration*, and *algorithm* sub-objects.

Table S23: Attributes table for the *citation* sub-object.

attribute	type	example	description	required	vocab
type	str	derived_from	key for identifier	True	True
reference	Reference		reference to a book, paper, or scholarly work	True	

3. CRIPT SUPPORTING NODES

For CRIPT's data structure to function properly in a software application the addition of a few additional nodes is needed to provide access control and access to external data storage. The inclusion of the *user* and *group* nodes provides access control, and *file* provides a link to external data storage.

3.1. User Node

The *user* node represents any researcher or individual who interacts with the CRIPT platform. It serves two main purposes: it plays a core role in permissions (access control), and it provides a traceable link to the individual who has contributed or edited data within the database. The required attributes are the 'username', 'email' and 'orcid'. The ORCID ID (open researcher and contributor ID) provides a unique and persistent digital identifier which can be used for login through the ORCID API (application programming interface).[5] The *user* node links to one or more *groups* that *user* is a member of.

Table S24: Attribute table for the *user* node.

attribute	type	example	description	required	vocab
url	str		unique ID of the node	True	
username	str	john_doe	User's name	True	
email	str	user@cript.com	email of the user	True	
orcid	str	0000-0000-0000-0000	ORCID ID of the user	True	
groups	list[Group]		groups you belong to		
updated_at	datetime*	2022-06-04T08:24:12.311266Z	last date the node was modified (UTC time)	True	
created_at	datetime*	2022-02-03T06:14:22.610253Z	date it was created (UTC time)	True	

* Datetime is formatted as International Organization for Standardization (ISO): yyyy-mm-ddThh:mm:ss.SSSZ string value. This applies for all future instances of datetime.

3.2. Group Node

The *group* node represents a grouping of *users* collaborating on a common project. It serves as the main permission control node and has ownership of data. *Groups* are the owners of data as most research groups have changing membership, and the data is typically owned by the organization and not the individuals. The required attributes are the 'name', 'admins', and 'users'. The 'name' attribute is required to be unique in the database to avoid confusions of multiple groups having the same name. By default, the *user* creating the *group* will become the 'admin' of the *group* and will also be added to the 'users' list. The 'admins' role is used by the permission control software to specify which *users* can add or delete other *users* to the group. Multiple 'admins' are allowed. All members of a *group* node will be able to view, edit, add, and delete data within the *group*. Given that permissions happen through the *group* node, all other nodes (except *user*, *reference*, and *software*) will link to the *group* node to establish the ownership relation.

Table S25: Attribute table for the *group* node.

attribute	type	example	description	required	vocab
url	str		unique ID of the node	True	
name	str	CRIPT development team	descriptive label	True	
notes	str		miscellaneous information, or custom data structure		
admins	list[User]		group administrators	True	
users	list[User]		group members	True	
updated_by	User		user that last updated the node	True	
created_by	User		user that originally created the node	True	
updated_at	datetime	2022-02-04T08:24:12.311266Z	last date the node was modified (UTC time)	True	
created_at	datetime	2022-02-03T06:11:21.610253Z	date it was created (UTC time)	True	

3.3. File

The *File* object contains the meta-data and link to a single raw data file, as raw data files are not directly stored in the database. The required attributes are the 'source', 'type', and 'name'. The 'source' is a link to the data file and will typically be an URL (uniform resource locator), but it could also be a file location if the data is being stored on a local server. The 'type' attribute specifies whether the file is data, a calibration file, configuration file, etc. The 'data_dictionary' attribute allows users to specify more specific meta-data about what is contained in the file, such as column and row headers with units. Ideally, the use of the 'data_dictionary' section will have a defined structure, like JSON, which can make it findable and interpretable with custom codes.

Table S26: Attributes table for the *File* object.

attribute	type	example	description	required	vocab
'base' attributes (see details above)					
source	str	url	link to file	True	
type	str	data	type of file	True	True
extension	str	.csv	extension		
data_dictionary	str		set of information describing the contents, format, and structure of a file		

To provide an explicit example of a 'data_dictionary' template, a trajectory export for computational data is provided. The trajectory export provides details for parsing and analyzing these large raw data files, such as evolving configurational and trajectory-related information. Attributes supported in this template include 'num_frame' (i.e., total number of frames dumped to the file node) 'dt_frame' (i.e., simulation time interval between two frames), 'dt_sim' (i.e., simulation time step), 'boundaries' and 'origin' of simulation box (duplicate these *conditions* here with the raw data as the storage of information might be centralized).

Table S27: Data dictionary template for computational trajectories.

keys	type	description	example	required	vocab
num_frame	int	total number of frames dumped to the file node	100		
dt_frame	float	simulation time interval between two frames	0.01		
dt_frame_unit	str	unit of time interval between two frames	ps		
dt_sim	float	simulation time step	0.1		
dt_sim_unit	str	unit of simulation time step			
boundaries	list[float]	nine element box matrix [e11,e12,e13, e21,e22,e23, e31,e32,e33]	[5,0,0, 0,5,0, 0,0,20]		
origin	list[float]	origin of box [o1,o2,o3]	[0,0,0]		

4. GRAPH EXAMPLES

Solid lines indicate reference between nodes.
Dashed lines indicate a reference originating from a sub-object within one of the nodes.
An arrowhead indicates a temporal relationship.
Colored lines have no additional meaning and are only to help with visualization.

4.1. Simple Chemical Reaction

The following is a generic CRIPT graph for a chemical reaction showing three ways to highlight distinct aspects. The top graph highlights the interconnection between the organizational nodes with the rest of the graph. The middle graph shows just the main graph. The bottom graph shows a graph where the process node is broken into multiple steps.

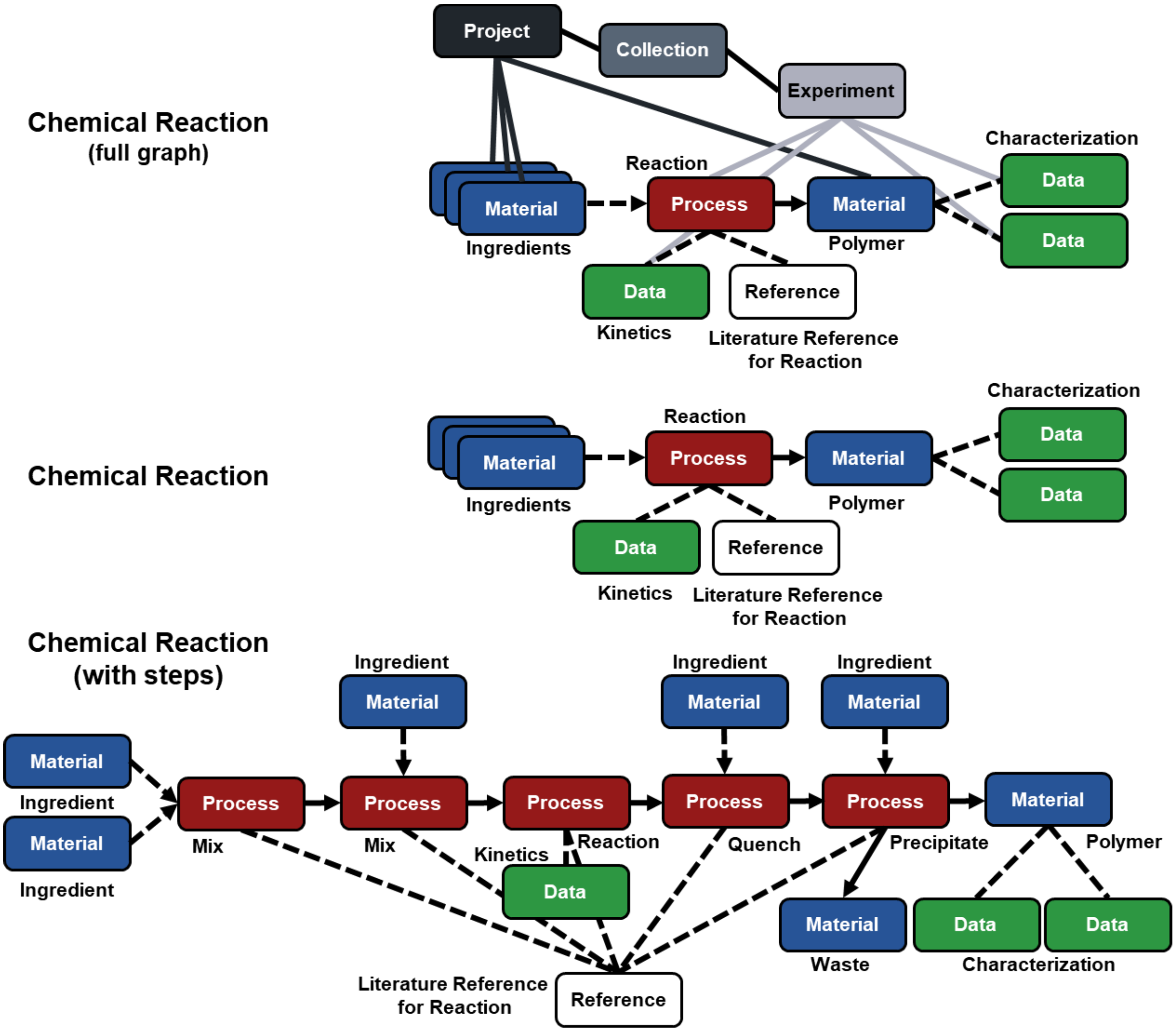

4.2. Diblock Copolymer Synthesis

The following depicts the graph for a diblock copolymer synthesis through sequential addition of monomers. Sequential addition refers to the synthesis for the first block (block A) followed by the growth of the second block (block B) directly off the first block. It is possible to condense the entire synthesis into a single process node; however, if characterization data is taken on the intermediate block A, then it is preferred to separate the process into two steps (as shown).

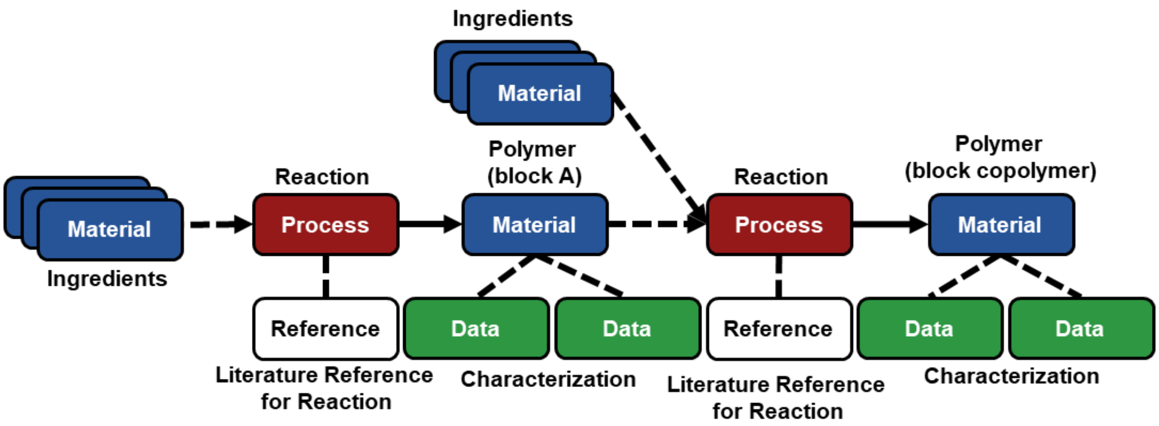

4.3. Reaction Kinetics

The following depicts the graph for a reaction kinetic analysis to determine the activation energy. This involves performing the reaction multiple times with different reaction temperatures (the *conditions* in the *process* node will be different). This example highlights how material nodes can be reused.

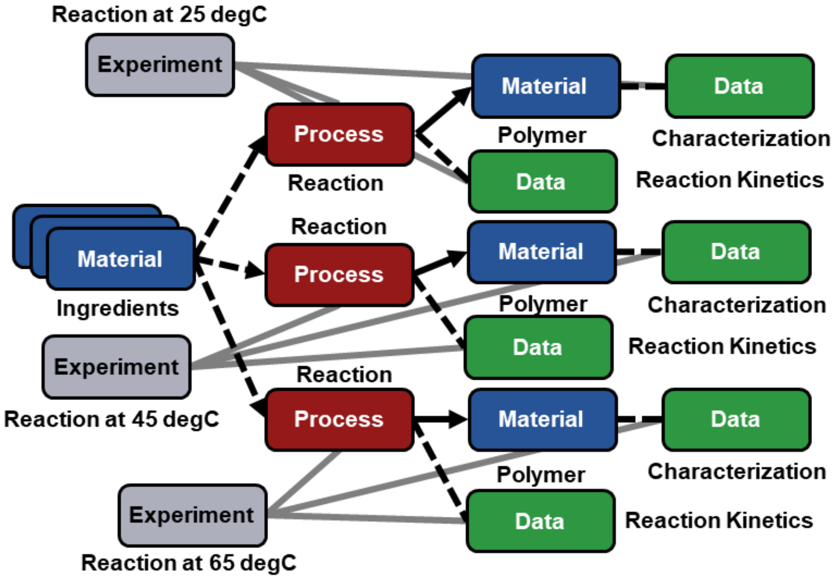

4.4. Diblock Bottlebrush Synthesis

The following depicts the graph for the synthesis of a diblock bottlebrush created by a graft-through sequential addition of macromonomers. This highlights a converging synthetic route where separate syntheses merge. Note that the convergence happens through the *process* node.

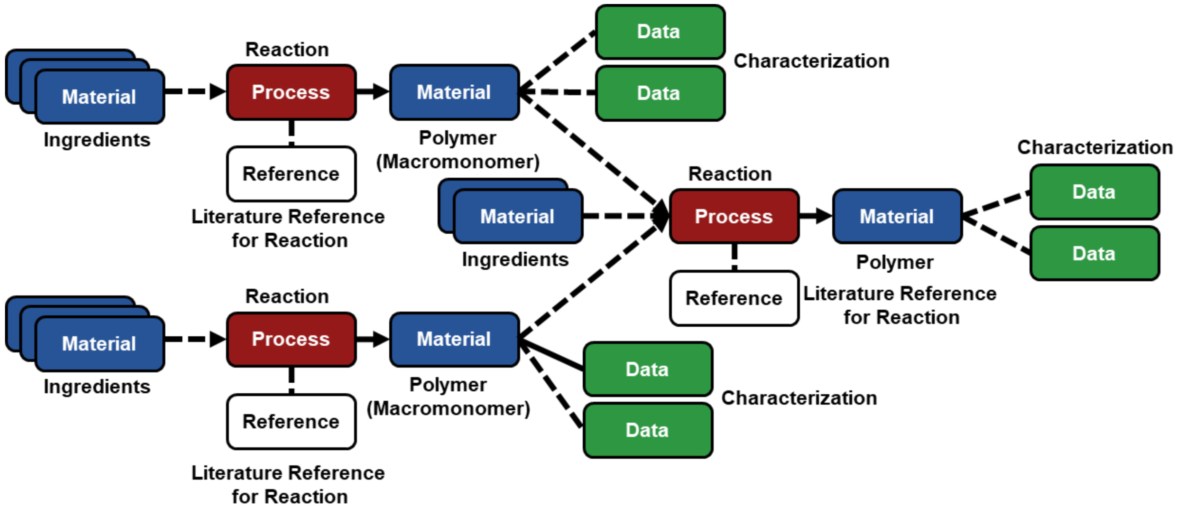

4.5. Chemical Reaction with Aliquots

The following depicts a chemical reaction where 2 aliquots were taken from a reaction, worked up, and characterized. This is an example of a diverging graph in which the *process* node (representing the chemical reaction) had to be split into 3 separate *process* nodes (labeled 'reaction') separated by *material* nodes (red bordered nodes). These intermediate nodes can be sparse in data and with only a single identifier needing to be specified. The splitting of the *process* node and the intermediate *material* nodes is needed to enable the user to specify the quantities of aliquot taken, the quantity of the reaction mixture that continues reacting, and at what time the aliquot was taken.

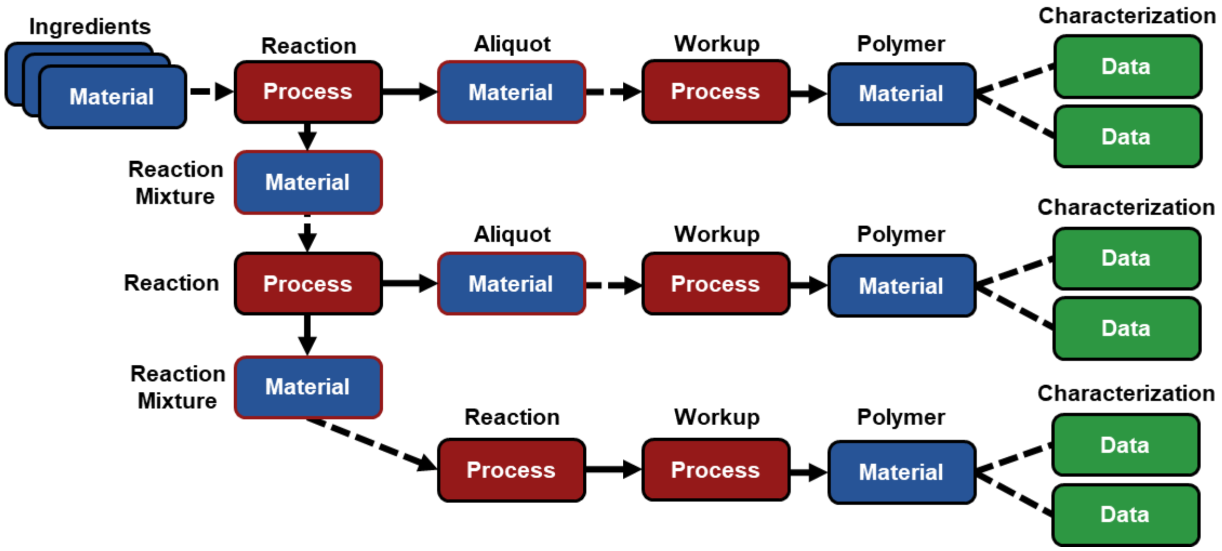

4.6. Multi-Step Chemical Reaction (Library Generation)

The following depicts the graph for a common approach to making a library of materials through two reactions. An example of this graph would be the synthesis of 3 diblock copolymers, in which the first block is the same, and the second block is made with different monomers or have different chain lengths (this is what is depicted below, as the second layer of *process* nodes use the same *material* nodes). The red border indicates an intermediate material node.

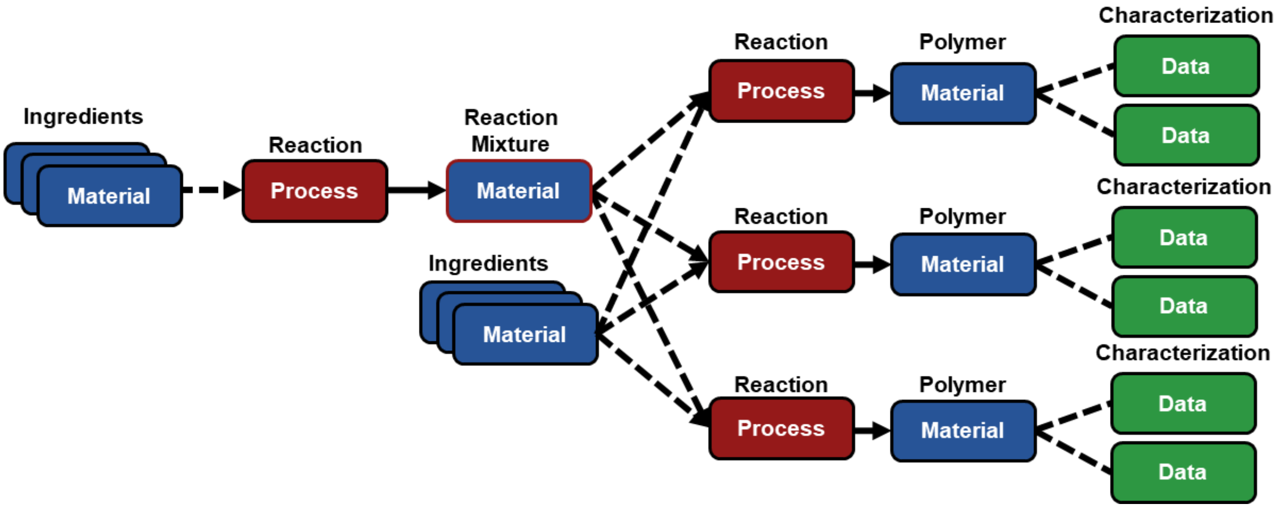

4.7. Material from Literature

The following depicts graphs for materials that are generated from reference literature. Raw data is attached with the use of a *data* node.

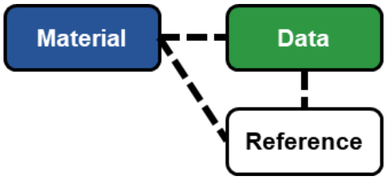

4.8. Characterization of Material

The following depicts a small graph for an experiment that solely involves the characterization of a material.

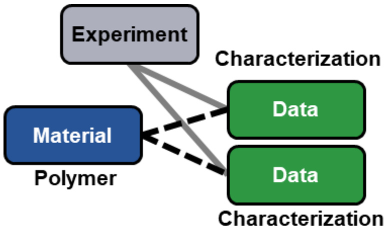

4.9. Involved Material Characterization

The following depicts a detailed graph for the characterization of a polymer with explicit reporting of the computational methods used to transform the raw data into the reported material properties. Abbreviations: small angle x-ray scattering (SAXS), radius of gyration (R_g), glass transition temperature (T_g), melting temperature (T_m), number average molecular mass (M_n), dispersity (Đ), molecular weight (MW), differential scanning calorimetry (DSC).

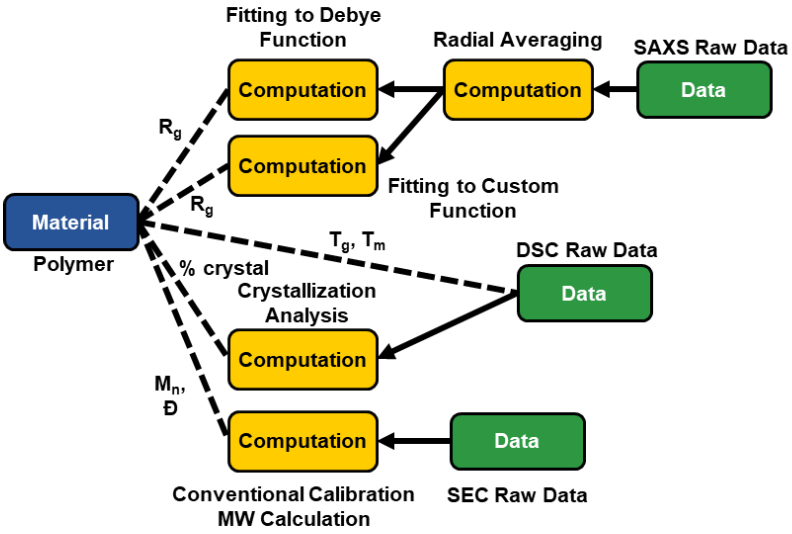

4.10. Block Copolymer Phase Behavior Annealing Study

The following depicts a graph for a study into the self-assembling behavior of a block copolymer in thin films and the effects of annealing. The block copolymer of interest was obtained from a vendor; thus, it was initially characterized prior to use. The various processing approaches were applied to samples of the block copolymer and similar characterization was performed on each of the formed films. Abbreviations: atomic force microscopy (AFM)

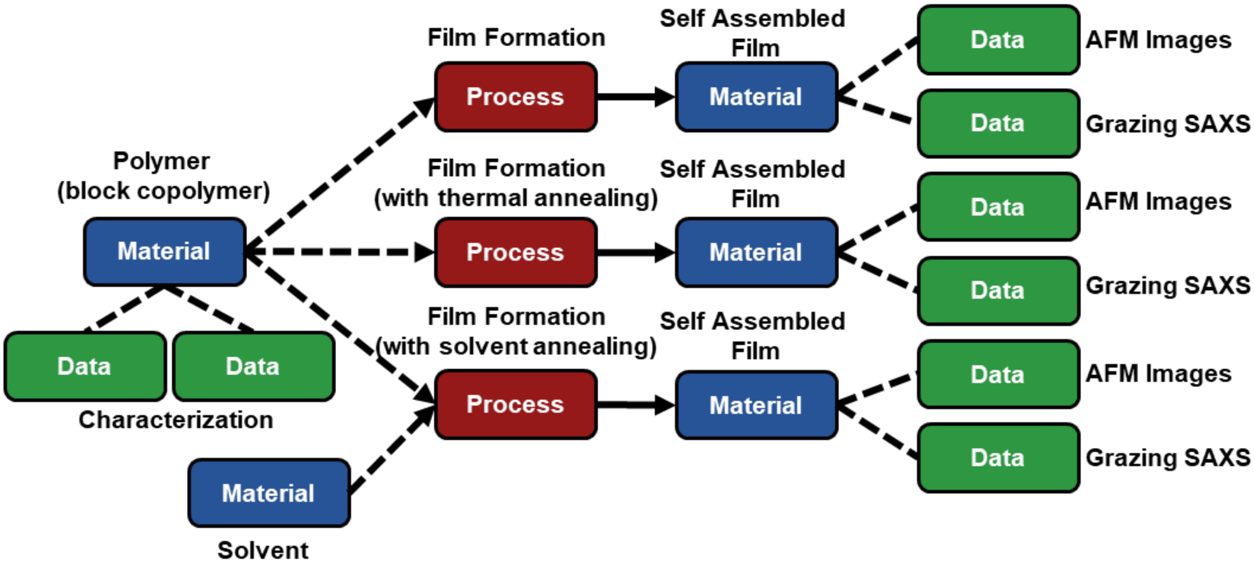

4.11. Extrusion and Characterization

The following depicts a graph for the formation of a composite material via an extrusion process. Following the synthesis, characterization of the produced material is performed.

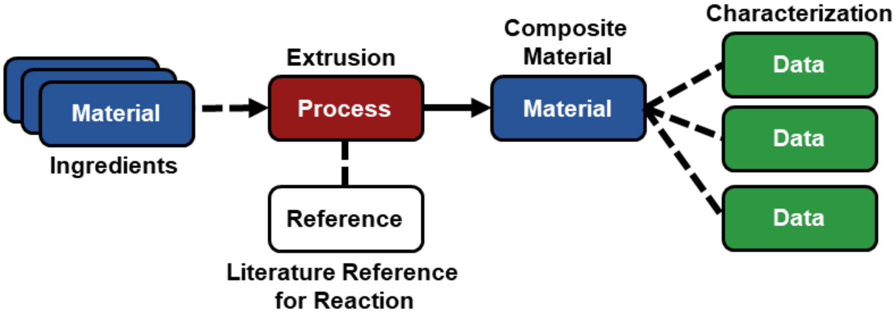

4.12. Computation to Produce Molecular Trajectories

The following depicts graphs of using molecular dynamics simulations to obtain the conformational information of poly(2,2'-dioxybiphenyl-phosphazene) in neat as well as in tetrahydrofuran (THF).[6] The simulations are carried out by DL-POLY (a general purpose parallel molecular dynamics simulation package by Daresbury Lab) with the input file placing a single oligomer in vacuum or THF solvent. A series of steps involving energy minimization, annealing, relaxation, etc. are used to produce the equilibrated molecular trajectory files by monitoring the evolution of system energy as well as density.

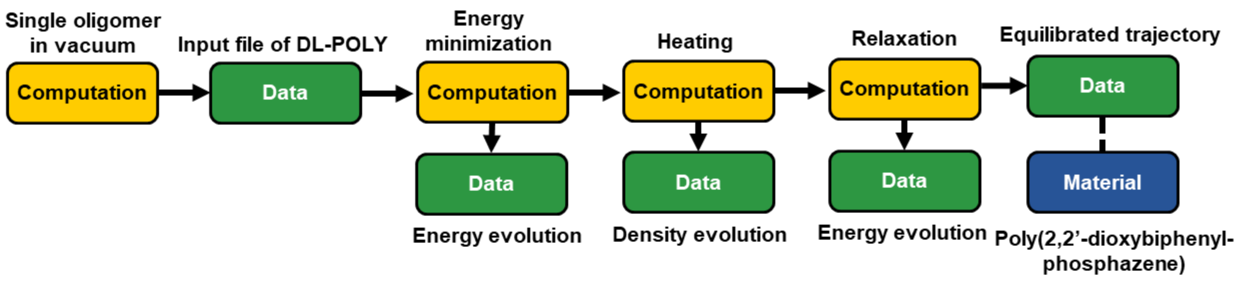

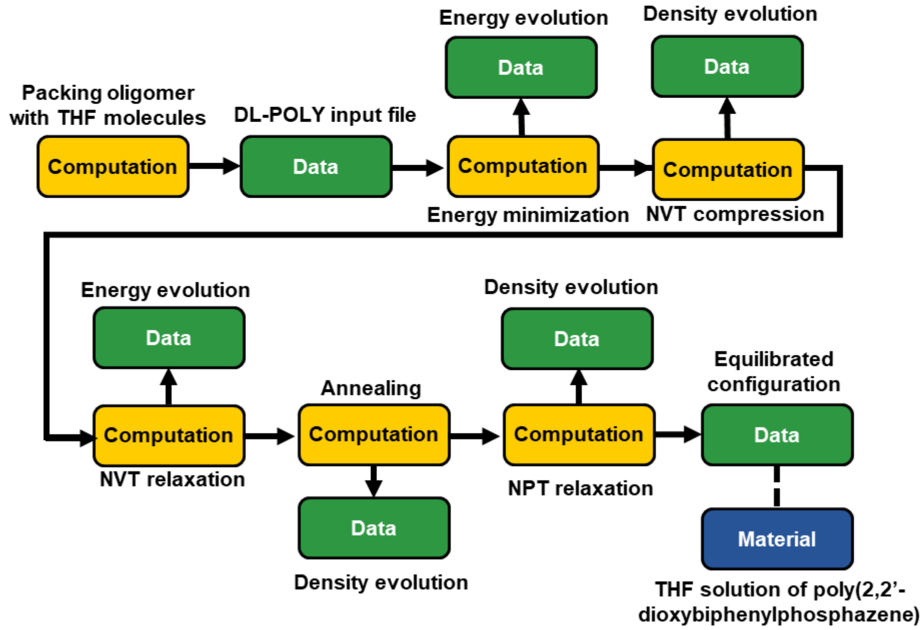

4.13. Computation to Simulate Reaction

The following graph depicts the molecular dynamics simulations which use ReaxFF (reactive force field) to investigate the thermal decomposition products of polydimethylsiloxane.[7] The simulations start with the quantum level calculations (density functional theory (DFT)), where a customized forcefield is generated for the initialization of ReaxFF (hybrid quantum and molecular dynamics simulation tool). After NVT based equilibration, the system and the corresponding material are subjected to simulated degradation. The reaction mechanism and population of degraded species are recorded and associated with a newly created material called "degraded polydimethylsiloxane".

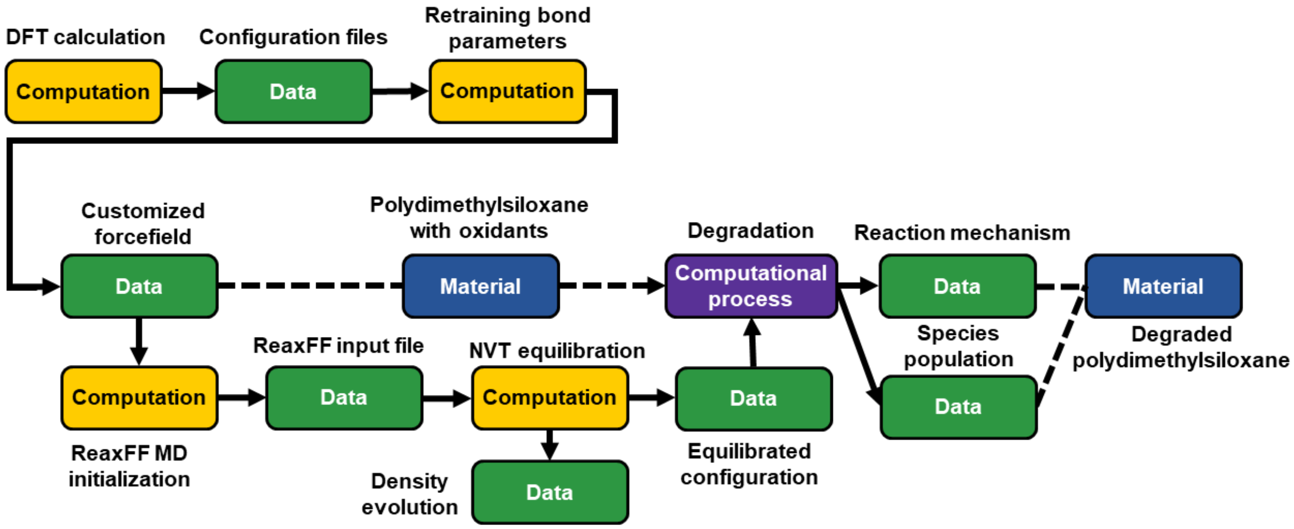

4.14. Computation to Generate Atomistic Forcefield

The following graph depicts the creation of atomistic forcefield for peptoids based on a modified CHARMM22 forcefield, with key parameters tuned to match quantum mechanical calculations.[8] *Ab initio* is first used to scan the energy profile with respect to the dihedral of the peptoid. The resulting data leads to the modification of standard CHARMM22 forcefield parameters and is further used to initialize the simulations of the dipeptoid in water. After equilibration, the system configuration undergoes umbrella sampling with the histogram analyzed by WHAM (Weighted Histogram Analysis Method) to determine the cis-/trans- free energy landscape.

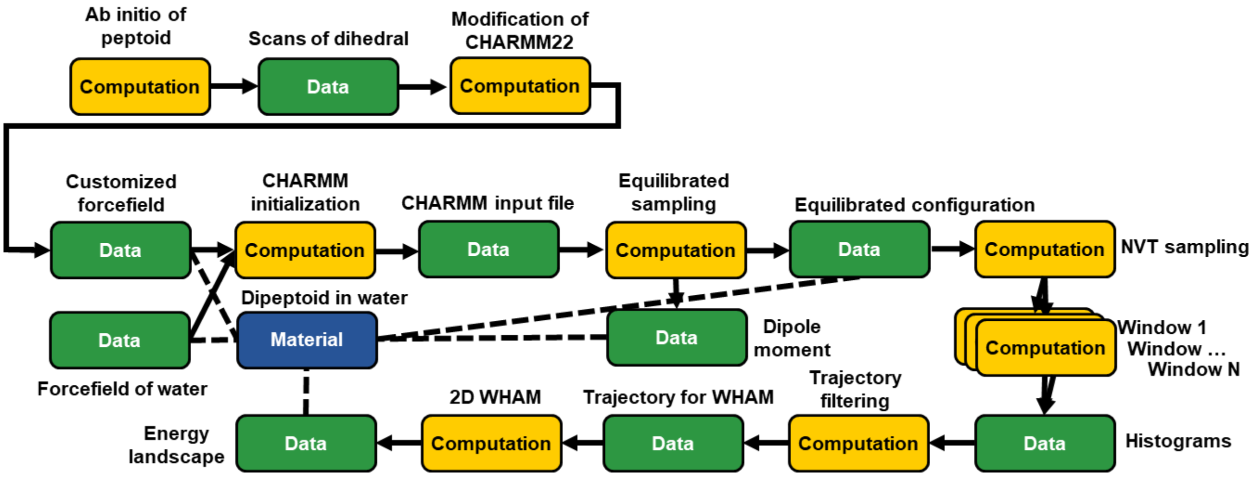

4.15. Computation to Estimate Kinetic Rates

The following graph depicts the use of different levels of quantum theories to estimate the reaction kinetics for free radical initiated grafting to polyolefins.[9] The initial guesses of computations are determined from the optimized molecular geometries of reactants and transition structure under lower level of DFT (B3LYP). Further optimization of the molecular geometries is performed under a fine-tuned DFT functional form by M06-2X. The resulting geometry are used for energy calculations executed by higher level of CCSDT (coupled cluster method with single double and perturbative triple). Finally, the kinetics were determined using Arkane (automated reaction kinetics and network exploration by Green's Lab at MIT) software by combining information from both optimized molecular geometry and energy files.

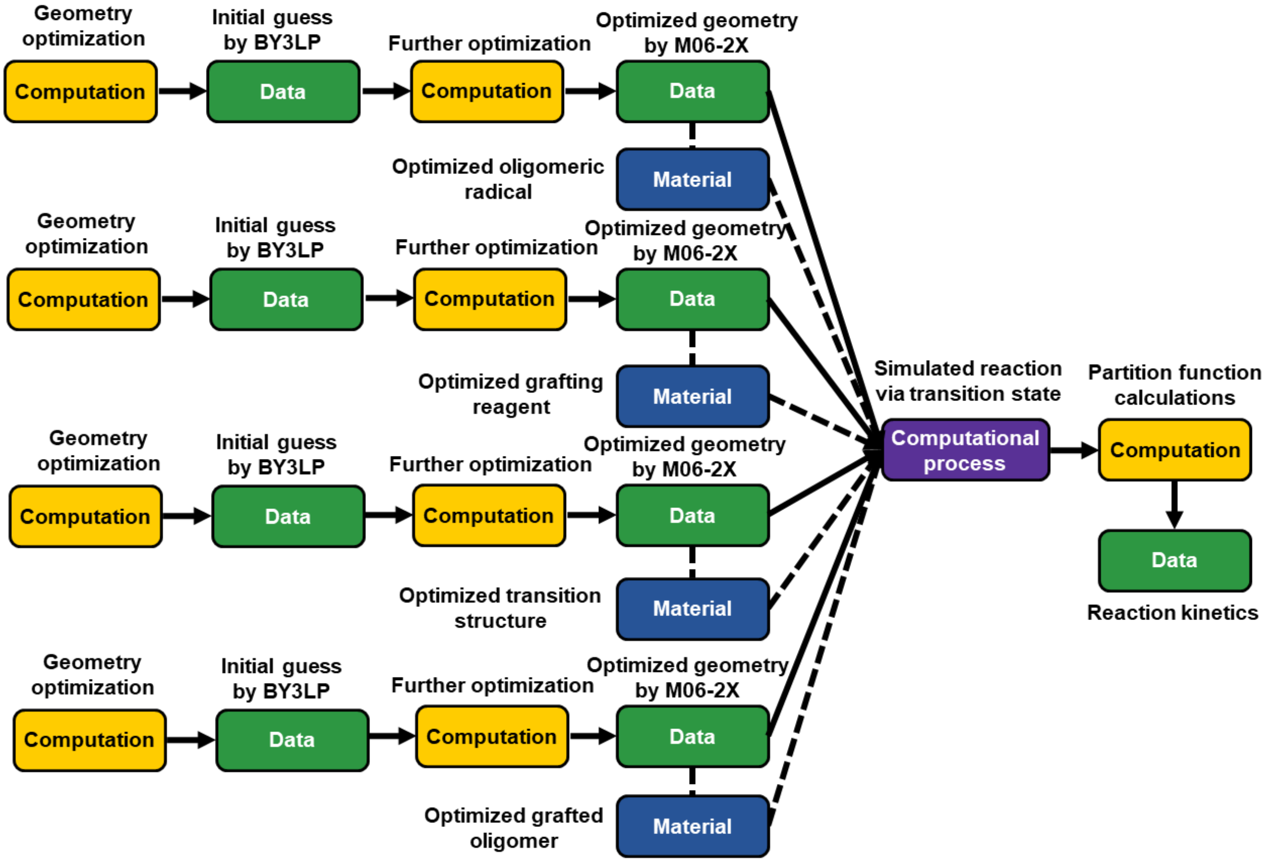

4.16. Collaboration Across Collections

A key design feature of the data model was to enable a single or multiple users to contribute to a project how they like while still maintaining the same representation. The top graph below depicts a collaboration of multiple people where all the users contribute through a single collection and single experiment. Alternatively, the bottom graph shows the same graph where the users contribute through their own research collection and experiment. Note that the core graph remains the same between the two examples.

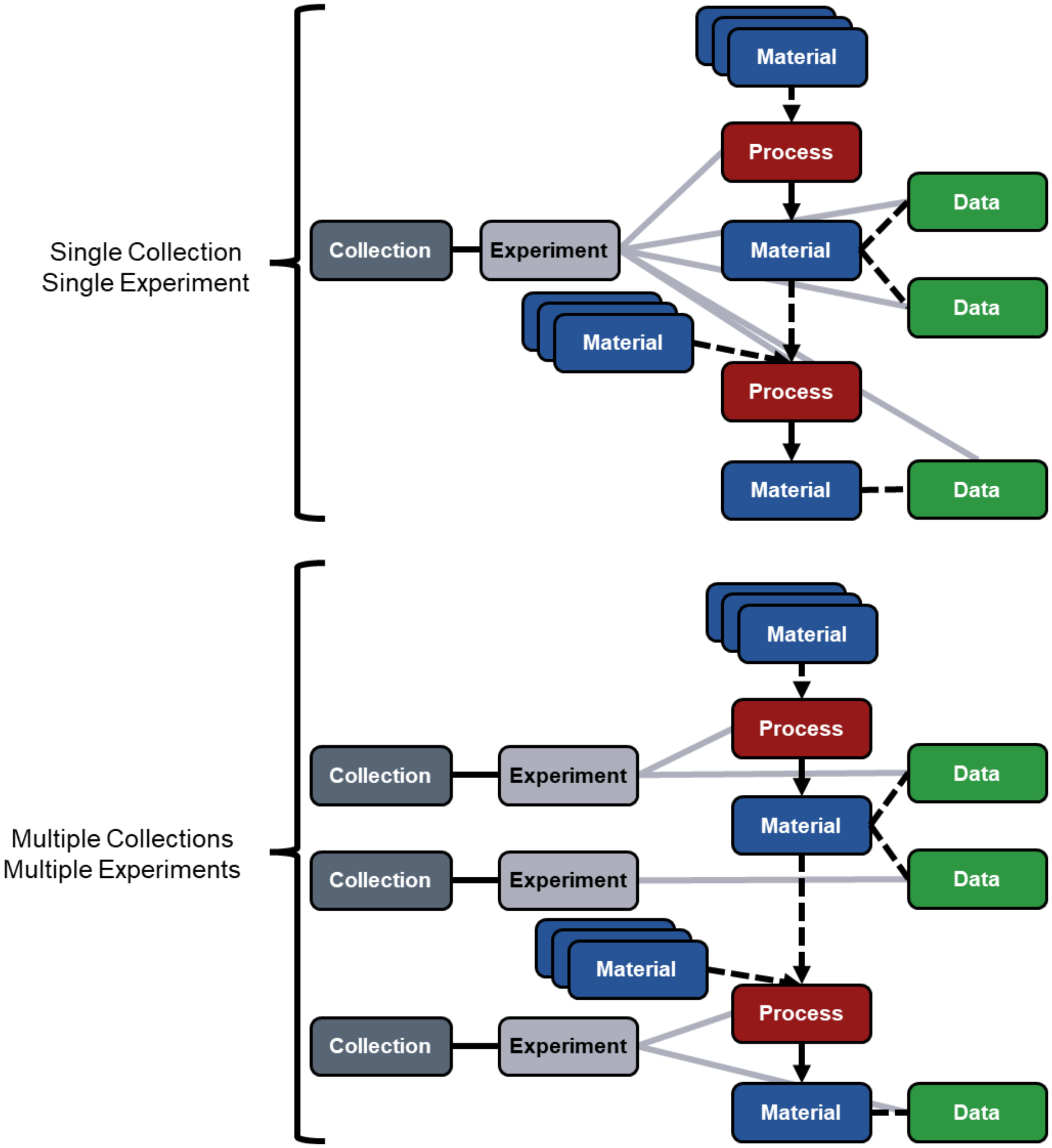

4.17. Collaboration Across Projects (With Common Users)

For *groups* wanting to collaborate, the data owned by one *group* can be copied to another *group* by a *user* that is a member of both. Copying was chosen over referencing nodes across *groups* to avoid issues of data integrity. For example, if a user referenced a *material* node from another *group* and then it was deleted, then there would be a broken *material* node reference leading to the loss of data integrity. (*Material* nodes with red outline represent the same material)

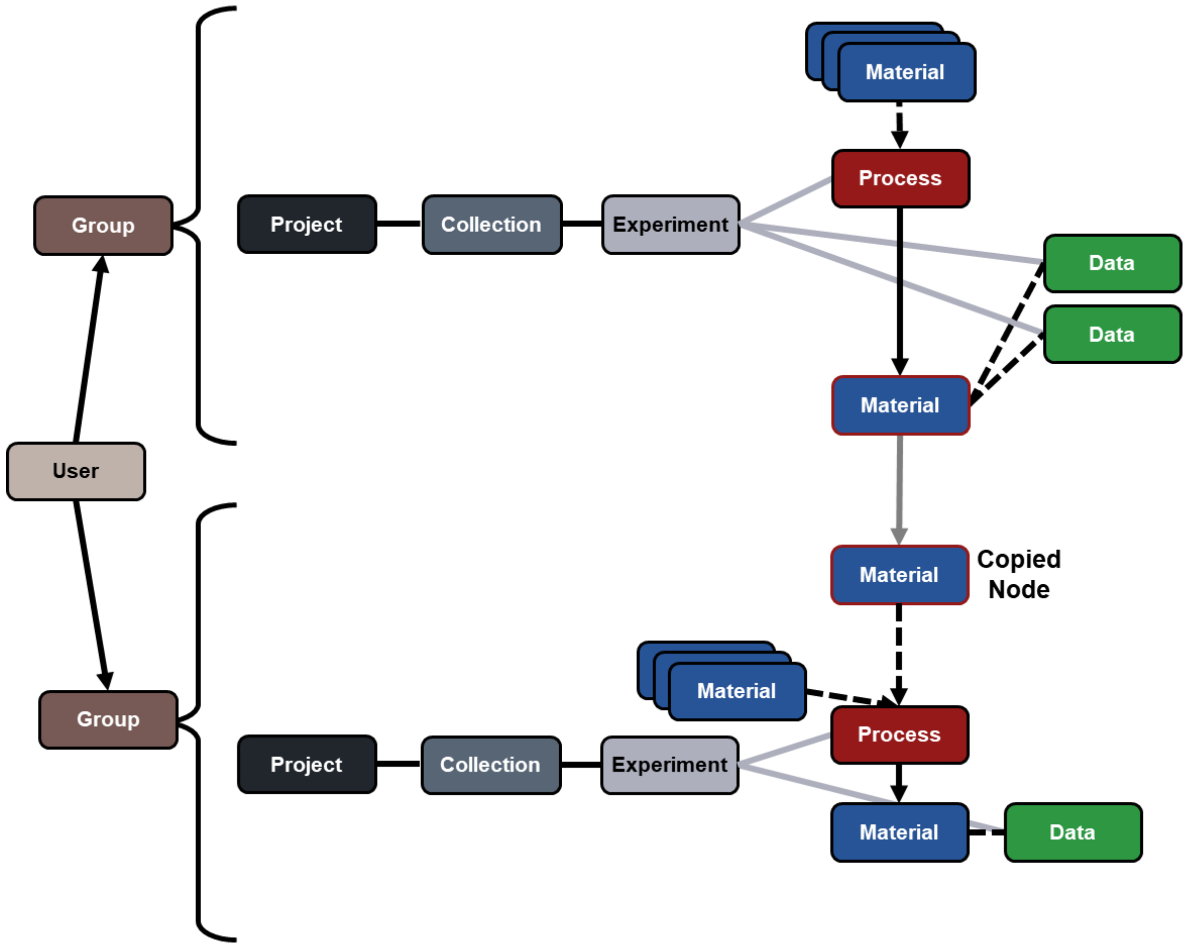

4.18. Collaboration Across Projects (Without Common Users)

For *groups* wanting to collaborate without having a *user* in common, the data owned by one *group/project* will need to be made public so that a second *group* can view it and copy it into their own *group/project*. The copied *material* node can be made public viewable or set to private.

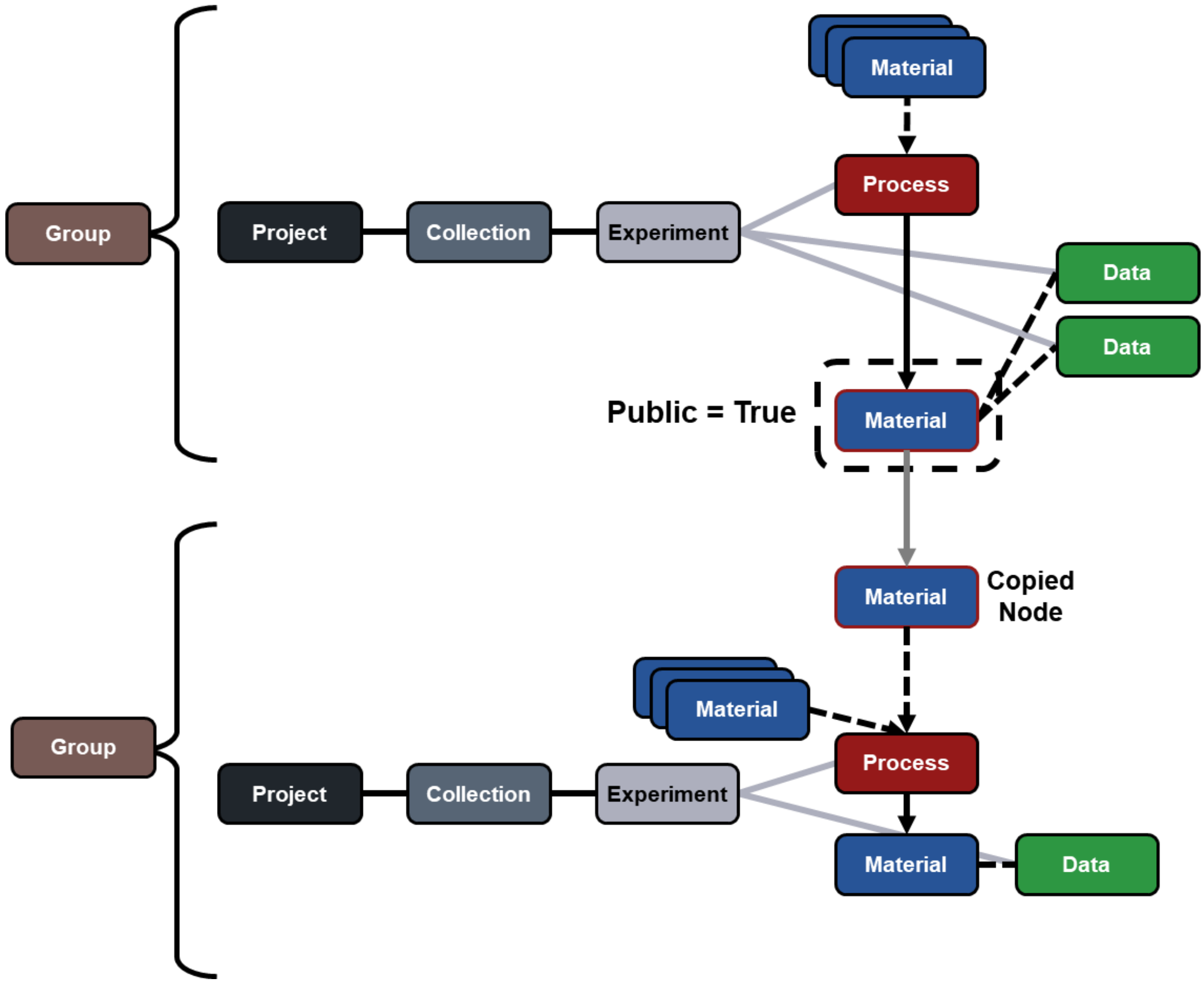

4.19. Access Control Within Projects

For a *project* wanting to have multiple levels of access control, multiple *groups* can be created that only have ownership of part of the *project*. Then *users* can be assigned to the appropriate *groups*. For example, *users* that should have full access to all the data can be assigned to all the groups. While *users* who should have partial access will only be assigned to a single *group*.

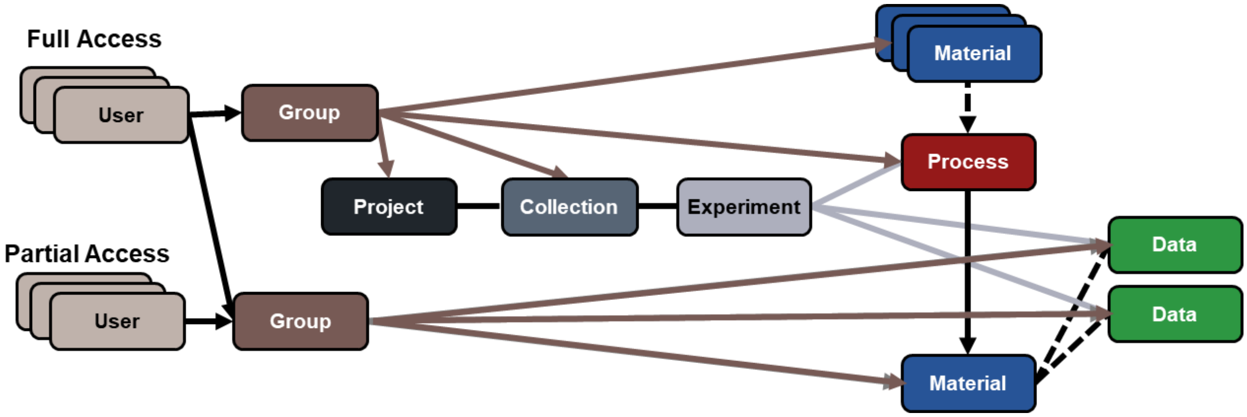

5. SUB-OBJECT EXAMPLES

The following contains visual depictions of individual nodes and the sub-object structure within the node.

5.1. Material Node

The following *material* node is for polystyrene produced by an anionic polymerization with molecular mass data from both NMR and SEC, and characterization of the glass temperature.

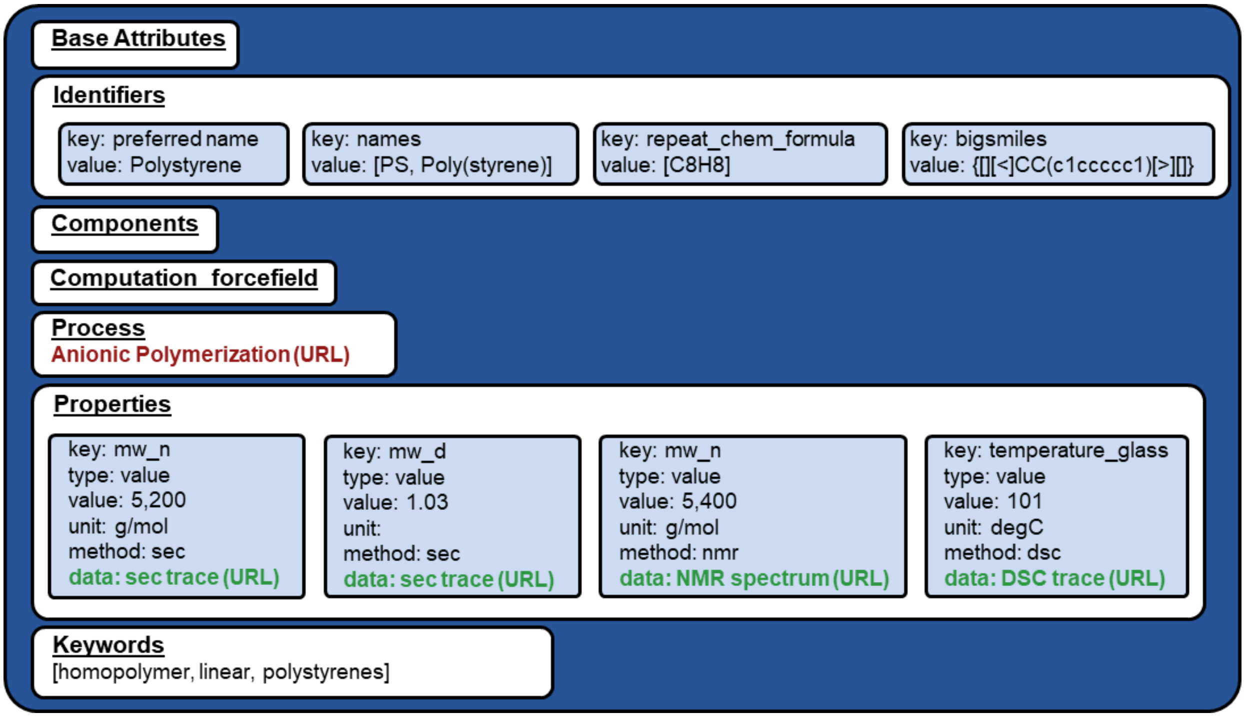

The following *material* node is for a polystyrene-poly(methyl methacrylate) diblock copolymer made via sequential addition anionic polymerization with molecular mass and composition specified.

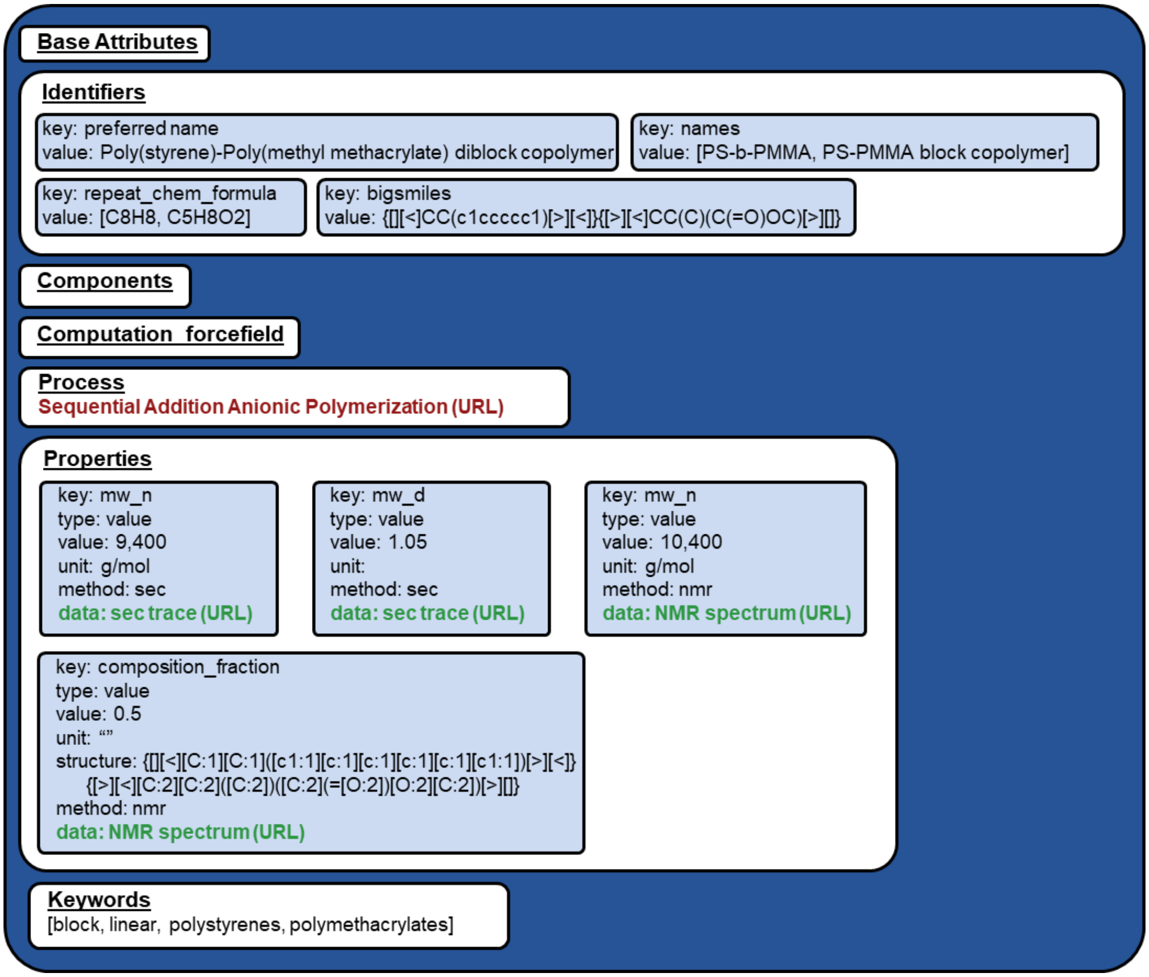

The following *material* node is for poly(3-butylthiophene-2,5-diyl) produced via a Kumada coupling with molecular mass and optical properties. With the optical properties, two different peaks and peak widths (as full-width-half-max: fwhm) were extracted and specified as properties.

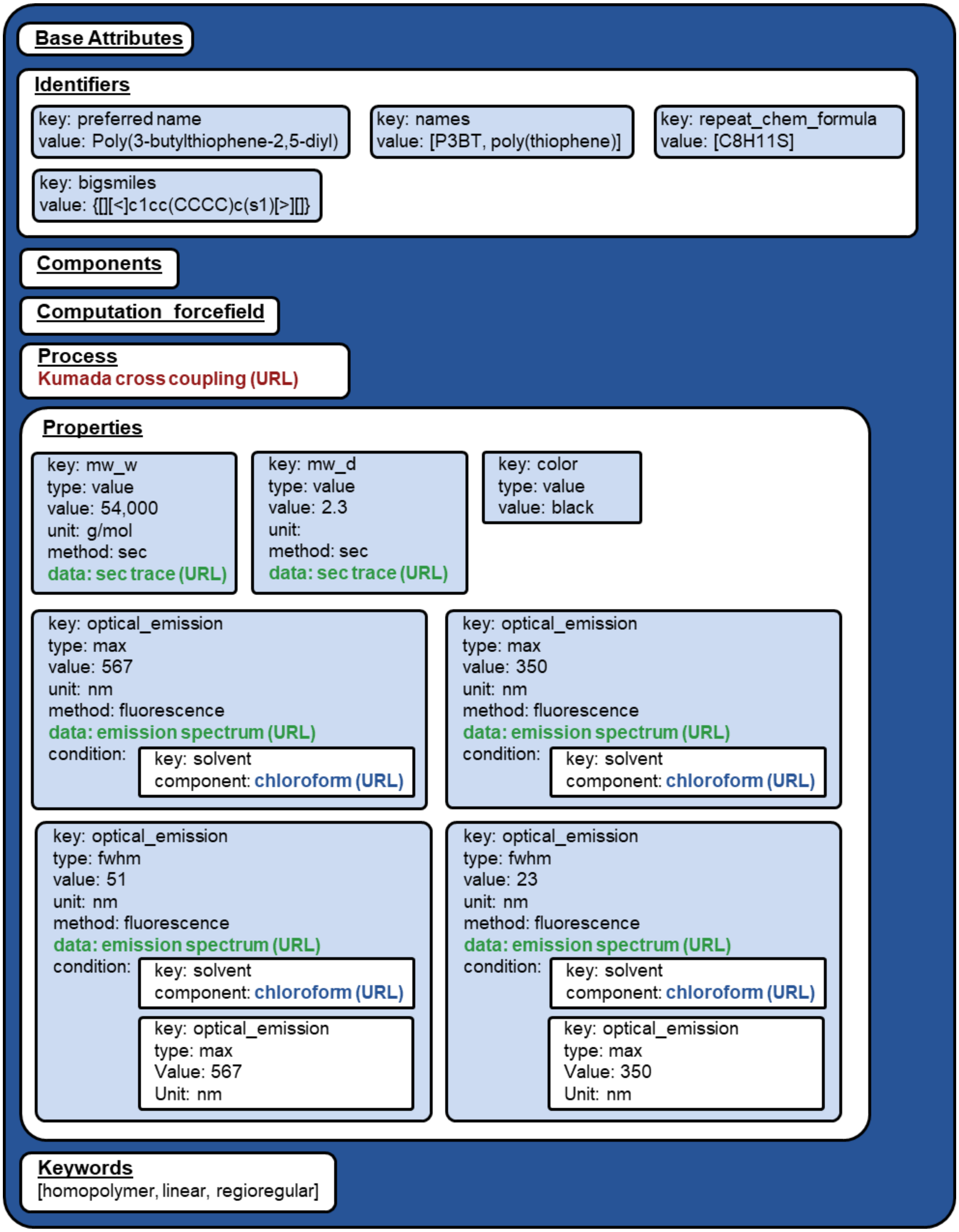

The following *material* node is for a mixture of lactic acid and poly(lactic acid) (PLA).

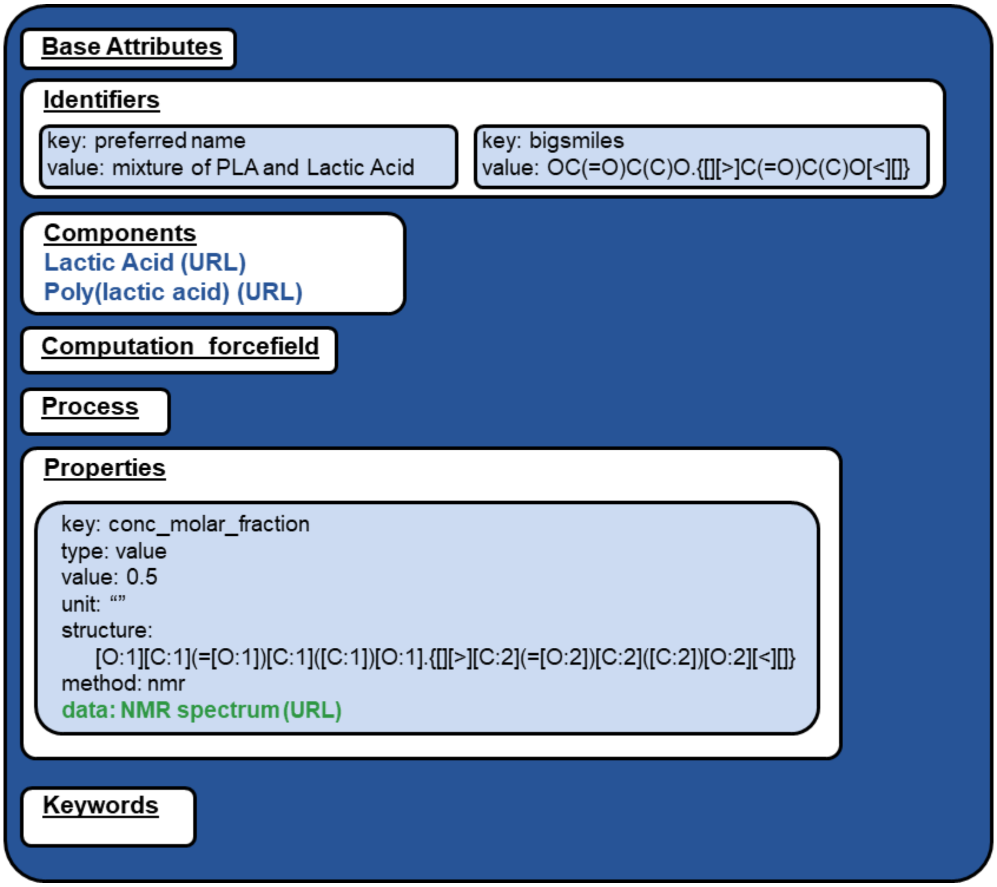

The following *material* node is for poly(ethylene glycol)-poly(lactic acid) (PEG-PLA) micelles in water. Abbreviation: dynamic light scattering (DLS)

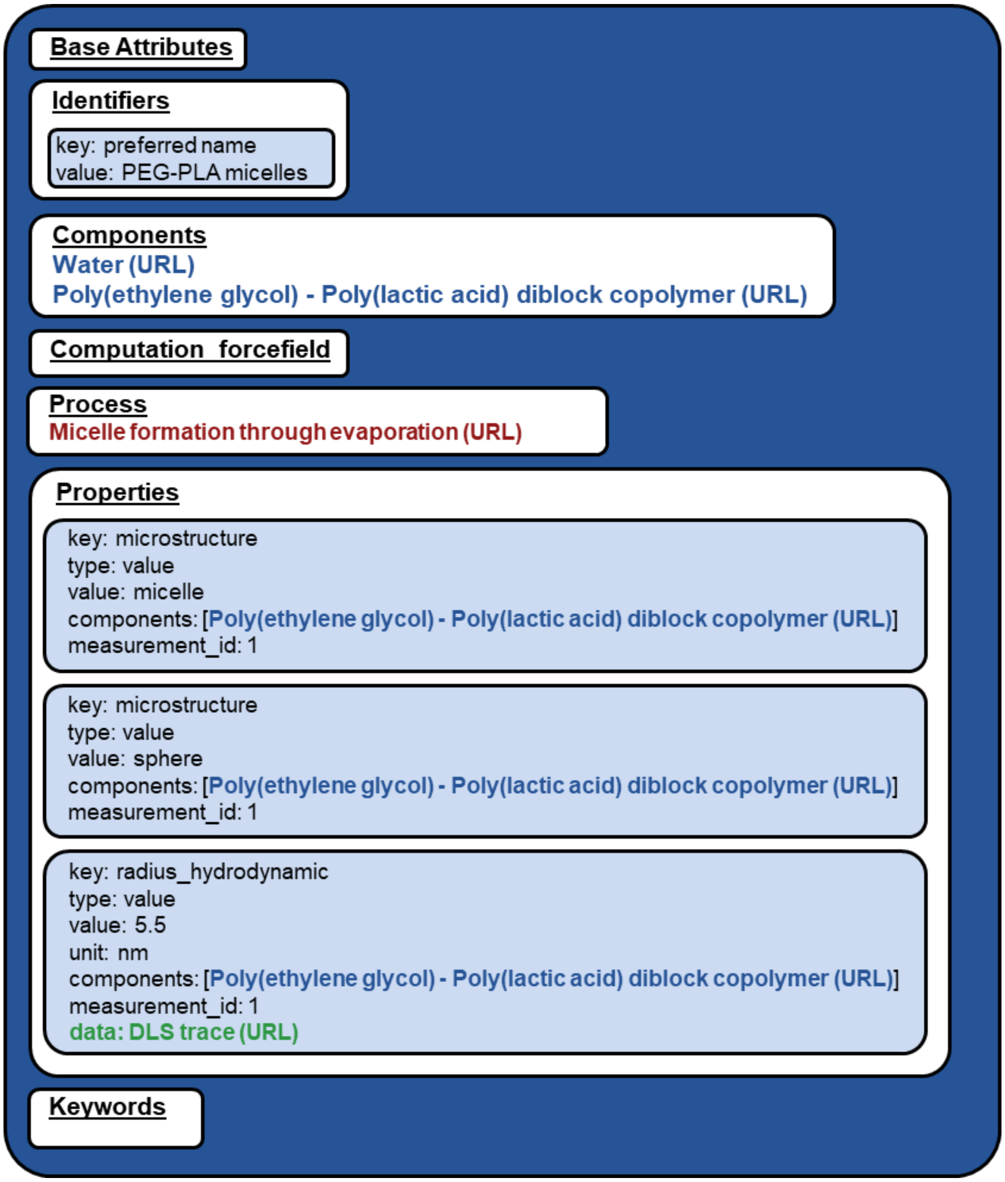

The following *material* node is for a isotactic polypropylene generated from computations.

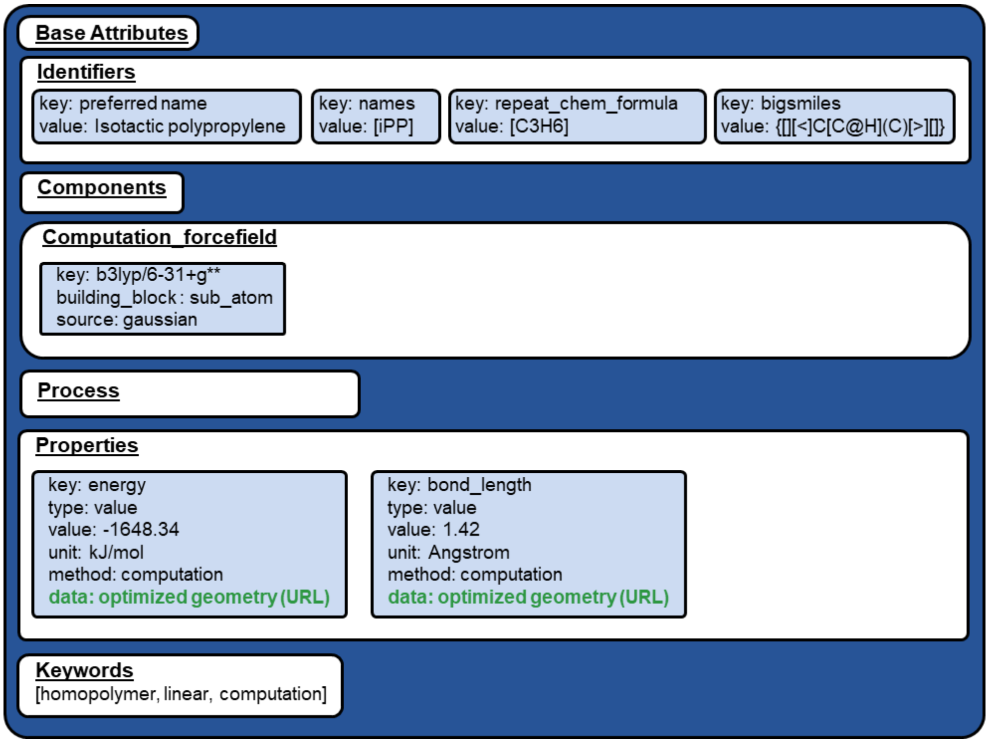

5.2. Process Node

The following *process* node is for the anionic polymerization of styrene. Throughout the process, the temperature was monitored every 15 minutes. Upon completion of the reaction, the yield of polymer was reported, and the conversion of styrene was calculated from NMR data. Abbreviation: secbutyl lithium (secBuLi)

Base Attributes

Type
multistep

Ingredients

material: **styrene (url)**
keyword: monomer
quantities:
- key: mass; value: 0.5; unit: gram
- key: volume; value: 0.55; unit: ml
- key: mole; value: 4.81; unit: mmol

material: **secBuLi solution (URL)**
keyword: initiator
quantities:
- key: volume; value: 0.5; unit: ml
- key: mole; value: 0.12; unit: mmol

material: **toluene (URL)**
keyword: solvent
quantities:
- key: mass; value: 8.67; unit: gram
- key: volume; value: 10; unit: ml
- key: mole; value: 94; unit: mmol

material: **methanol (URL)**
keyword: workup
quantities:
- key: mass; value: 79.2; unit: gram
- key: volume; value: 100; unit: ml
- key: mole; value: 2.4; unit: mol

Description
SecBuLi was added to a vial containing toluene. With vigorous stirring, styrene was added to initiate the polymerization and orange color is observed. The reaction proceeded for 45 mins, at which time the reaction was poured into methanol and a white solid was collected with vacuum filtration.

Conditions

- key: time_point; type: value; value: 0; unit: min; set_id: 1; measument_id: 1
- key: temperature; type: value; value: 25; unit: degC; set_id: 1; measument_id: 1
- key: time_point; type: value; value: 15; unit: min; set_id: 1; measument_id: 2
- key: temperature; type: value; value: 38; unit: degC; set_id: 1; measument_id: 2
- key: time_point; type: value; value: 30; unit: min; set_id: 1; measument_id: 3
- key: temperature; type: value; value: 32; unit: degC; set_id: 1; measument_id: 3
- key: time_point; type: value; value: 45; unit: min; set_id: 1; measument_id: 4
- key: temperature; type: value; value: 27; unit: degC; set_id: 1; measument_id: 4
- key: time_duration; type: value; value: 45; unit: min
- key: atmosphere; component: **Argon (URL)**

Properties

- key: yield_mass; value: 0.46; unit: gram; method: scale
- key: conversion; type: max; value: 0.98; unit: ; components: [**Styrene (URL)**]; method: NMR; **data: ^{1}H NMR (URL)**

Citations:

- type: replicated; reference: **'Living' Polymers, Szwarc 1956 (URL)**

6. CONTROLLED VOCABULARY, KEYS AND KEYWORDS

A full list of key words can be found at: https://criptapp.org/keys/

In general, 'snake case' styling is used. This refers to the name formalism where each space is replaced by an underscore '_' character, and everything is written in lowercase.

Nodes:

- Material → keywords
- Process → type
- Process → keywords
- Data → type
- Computation → type
- Computational_process → type
- Reference → type

Sub-objects:

- Identifier → key
- Property → key
    - Material
    - Process
    - Computational_process
- Property → type
- Property → uncertainty_type
- Property → method
- Condition → key
- Condition → type (same as Property → type)
- Condition → uncertainty_type (same as Property → uncertainty_type)
- Ingredient → keyword
- Quantity → key
- Quantity → uncertainty_type (same as Property → uncertainty_type)
- Equipment → key
- Computational_forcefield → key
- Computational_forcefield → building_block
- Algorithm → key
- Parameter → key
- Citation → type

Supporting Nodes

- File → type

7. REFERENCE

(1) Weininger, D. SMILES, a Chemical Language and Information System. 1. Introduction to Methodology and Encoding Rules. *J Chem Inf Model* **1988**, *28* (1), 31–36. https://doi.org/10.1021/ci00057a005.

(2) Lin, T.-S.; Coley, C. W.; Mochigase, H.; Beech, H. K.; Wang, W.; Wang, Z.; Woods, E.; Craig, S. L.; Johnson, J. A.; Kalow, J. A.; Jensen, K. F.; Olsen, B. D. BigSMILES: A Structurally-Based Line Notation for Describing Macromolecules. *ACS Cent Sci* **2019**, *5* (9), 1523–1531. https://doi.org/10.1021/acscentsci.9b00476.

(3) National Institutes of Health. *PubChem*. https://pubchem.ncbi.nlm.nih.gov/ (accessed 2022-01-01).

(4) Heller, S. InChI – the Worldwide Chemical Structure Standard. *J Cheminform* **2014**, *6* (S1), P4. https://doi.org/10.1186/1758-2946-6-S1-P4.

(5) ORCID. *ORCID*. https://orcid.org/ (accessed 2021-12-31).

(6) Laguna, M. T. R.; Tarazona, M. P.; Carriedo, G. A.; García Alonso, F. J.; Fidalgo, J. I.; Saiz, E. Thermal Degradation and Solution Properties of Poly(2,2‘-Dioxybiphenyl Phosphazene). *Macromolecules* **2002**, *35* (19), 7505–7515. https://doi.org/10.1021/MA020588P.

(7) Chenoweth, K.; Cheung, S.; van Duin, A. C. T.; Goddard, W. A.; Kober, E. M. Simulations on the Thermal Decomposition of a Poly(Dimethylsiloxane) Polymer Using the ReaxFF Reactive Force Field. *J Am Chem Soc* **2005**, *127* (19), 7192–7202. https://doi.org/10.1021/JA050980T/SUPPL_FILE/JA050980TSI20050324_061942.PDF.

(8) Mirijanian, D. T.; Mannige, R. v; Zuckermann, R. N.; Whitelam, S. Development and Use of an Atomistic CHARMM-Based Forcefield for Peptoid Simulation. https://doi.org/10.1002/jcc.23478.

(9) Zou, W.; Tupper, A.; Rebello, N. J.; Ranasinghe, D. S.; Green, W. H.; Couch, C.; Olsen, B. D. Multiscale Modeling and Characterization of Radical-Initiated Modification of Molten Polyolefins. *Macromolecules* **2022**, *55* (14), 5901–5915. https://doi.org/10.1021/ACS.MACROMOL.2C00202.
