# Supplementary material for: Community Resource for Innovation in Polymer Technology (CRIPT): A Scalable Polymer Material Data Structure
Source: ACS Cent Sci. 2023 Feb 20;9(3):330–8. doi: 10.1021/acscentsci.3c00011 (PMC10037456; doi:10.1021/acscentsci.3c00011)
Supplement: Supplementary file 2 — oc3c00011_si_002.pdf [file oc3c00011_si_002.pdf]

Supporting Information for:

Community Resource for Innovation in Polymer Technology (CRIPT): A Scalable Polymer Material Data Structure

Dylan J. Walsh[a], Weizhong Zou[a], Ludwig Schneider[b], Reid Mello[a], Michael E. Deagen[a], Joshua Mysona[b], Tzyy-Shyang Lin[a], Juan J. de Pablo[b], Klavs F. Jensen[a], Debra J. Audus[c], Bradley D. Olsen*[a]

[a]Department of Chemical Engineering Massachusetts Institute of Technology, 77 Massachusetts Avenue, Cambridge, Massachusetts 02139, United States

[b]Pritzker School of Molecular Engineering, University of Chicago, Chicago, IL 60637, USA.

[c]Materials Science and Engineering Division, National Institute of Standards and Technology, Gaithersburg, Maryland 20899, United States

WRITTEN OUT EXAMPLE OF THE DATA MODEL

The following contains the full data model representation for example from Figure 6 of the main manuscript (chemical synthesis of polystyrene made by the secBuLi initiated anionic polymerization of styrene).

User **3**

Group **3**

Project **3**

Collection **4**

Experiment **4**

Inventory **4**

Materials **5**

Nitrogen *5*

Argon *10*

Tetrahydrofuran *14*

Toluene *20*

Water *25*

Methanol *30*

Styrene *34*

1-Butanol *39*

Sec-Butyl Lithium *43*

Sec-Butyl Lithium Solution *45*

Poly(styrene) *48*

Deuterated Chloroform *51*

Process **55**

Data **59**

^{1}H Nuclear Magnetic Resonance Spectrum *59*

Size Exclusion Chromatography Trace *60*

File **60**

^{1}H Nuclear Magnetic Resonance Spectrum *60*

Size Exclusion Chromatography Trace *61*

Reference *61*

USER

{
  "url": "local://cript/user/2aaa30ea-2b13-4804-92cf-a939bac745ac/",
  "uid": "2aaa30ea-2b13-4804-92cf-a939bac745ac",
  "public": false,
  "created_at": "2022-09-08T13:48:09.469494",
  "updated_at": "2022-09-08T13:48:09.469494",
  "username": "Tim",
  "email": "tim@cript.edu",
  "orcid_id": "",
  "groups": [],
  "model_version": "0.4.3"
}

GROUP

{
  "url": "local://cript/group/5a5a9fb2-44ca-41e7-931d-b544a9a02b86/",
  "uid": "5a5a9fb2-44ca-41e7-931d-b544a9a02b86",
  "public": false,
  "created_at": "2022-09-08T13:48:09.470495",
  "updated_at": "2022-09-08T13:48:09.470495",
  "name": "SDK test group",
  "users": [],
  "model_version": "0.4.3"
}

PROJECT

{
  "url": "local://cript/project/ef26f322-8e03-4655-9aca-a731b6e5ae36/",
  "uid": "ef26f322-8e03-4655-9aca-a731b6e5ae36",
  "public": false,
  "created_at": "2022-09-08T13:48:09.470495",
  "updated_at": "2022-09-08T13:48:09.470495",
  "name": "SDK test project",
  "materials": [],
  "files": [],
  "collections": [],
  "group": "local://cript/group/5a5a9fb2-44ca-41e7-931d-b544a9a02b86/",
  "model_version": "0.4.3"
}

COLLECTION

{
  "url": "local://cript/collection/fa73f5c7-75c5-4b5e-9390-955aefa2854f/",
  "uid": "fa73f5c7-75c5-4b5e-9390-955aefa2854f",
  "public": false,
  "created_at": "2022-09-08T13:48:09.471494",
  "updated_at": "2022-09-08T13:48:10.326308",
  "project": "local://cript/project/ef26f322-8e03-4655-9aca-a731b6e5ae36/",
  "name": "Tutorial Examples",
  "experiments": [
    "local://cript/inventory/551c3b02-0185-4d57-80ac-394ca697f800/"
  ],
  "inventories": [],
  "citations": [],
  "notes": null,
  "group": "local://cript/group/5a5a9fb2-44ca-41e7-931d-b544a9a02b86/",
  "model_version": "0.4.3"
}

EXPERIMENT

{
  "url": "local://cript/experiment/37422b5c-fa9e-4377-b3de-18ef3105f0f4/",
  "uid": "37422b5c-fa9e-4377-b3de-18ef3105f0f4",
  "public": false,
  "created_at": "2022-09-08T13:48:09.471494",
  "updated_at": "2022-09-08T13:48:09.471494",
  "collection": "local://cript/collection/fa73f5c7-75c5-4b5e-9390-955aefa2854f/",
  "name": "Anionic Polymerization of Styrene with SecBuLi",
  "funding": [],
  "processes": [],
  "data": [],
  "notes": null,
  "group": "local://cript/group/5a5a9fb2-44ca-41e7-931d-b544a9a02b86/",
  "model_version": "0.4.3"
}

INVENTORY

{
  "url": "local://cript/inventory/551c3b02-0185-4d57-80ac-394ca697f800/",
  "uid": "551c3b02-0185-4d57-80ac-394ca697f800",
  "public": false,
  "created_at": "2022-09-08T13:48:10.323309",
  "updated_at": "2022-09-08T13:48:10.323309",
  "collection": "local://cript/collection/fa73f5c7-75c5-4b5e-9390-955aefa2854f/",

    "name": "Tutorial Materials",
    "description": null,
    "materials": [
        "local://cript/material/cf7d4d83-88d7-4a3a-9eec-5f707c806b3b/",
        "local://cript/material/f8f76b12-12f3-4bf2-880d-75f13535e994/",
        "local://cript/material/8ddb53c1-738e-4c11-81c1-0e1e4fb53e84/",
        "local://cript/material/922c134c-99fc-4aac-ab22-4796dd3c6381/",
        "local://cript/material/c46b68d8-b521-4851-b85e-632ad3d02e96/",
        "local://cript/material/63f071c2-c64a-4b71-a84b-8de5b5812f4c/",
        "local://cript/material/46077c6a-1657-4d14-a8e5-645be25af625/",
        "local://cript/material/b719064a-0ece-4990-a0eb-f0e0fffab1e3/",
        "local://cript/material/df9e5147-a5cc-49e1-b11a-23dcc7263d3c/",
        "local://cript/material/89a49359-bb0a-40ba-8b0e-c4fbc02c7907/",
        "local://cript/material/3bc36780-f200-4954-9d58-2108e30c83a9/"
    ],
    "group": "local://cript/group/5a5a9fb2-44ca-41e7-931d-b544a9a02b86/",
    "model_version": "0.4.3"
}

MATERIALS

Nitrogen

{
    "url": "local://cript/material/8ddb53c1-738e-4c11-81c1-0e1e4fb53e84/",
    "uid": "8ddb53c1-738e-4c11-81c1-0e1e4fb53e84",
    "public": false,
    "created_at": "2022-09-08T13:48:10.313309",
    "updated_at": "2022-09-08T13:48:10.313309",
    "project": "local://cript/project/ef26f322-8e03-4655-9aca-a731b6e5ae36/",
    "name": "nitrogen",
    "identifiers": [
        {
            "key": "preferred_name",
            "value": "nitrogen"
        },
        {
            "key": "names",
            "value": [
                "N2",
                "dinitrogen"
            ]
        },
        {
            "key": "cas",
            "value": "7727-37-9"
        },

{
"key": "smiles",
"value": "N#N"
},
{
"key": "chem_formula",
"value": "N2"
},
{
"key": "pubchem_cid",
"value": 947
},
{
"key": "inchi_key",
"value": "IJGRMHOSHXDMSA-UHFFFAOYSA-N"
}
],
"components": [],
"keywords": [],
"process": null,
"properties": [
{
"key": "phase",
"unit": null,
"value": "gas",
"type": null,
"method": null,
"method_description": null,
"sample_preparation": null,
"uncertainty": null,
"uncertainty_type": null,
"components": [],
"components_relative": [],
"structure": null,
"set_id": null,
"conditions": [],
"data": null,
"citations": [],
"notes": null
},
{
"key": "color",
"unit": null,
"value": "colorless",
"type": null,
"method": null,

"method_description": null,
"sample_preparation": null,
"uncertainty": null,
"uncertainty_type": null,
"components": [],
"components_relative": [],
"structure": null,
"set_id": null,
"conditions": [],
"data": null,
"citations": [],
"notes": null
},
{
"key": "molar_mass",
"unit": "g/mol",
"value": 28.014,
"type": null,
"method": "prescribed",
"method_description": null,
"sample_preparation": null,
"uncertainty": null,
"uncertainty_type": null,
"components": [],
"components_relative": [],
"structure": null,
"set_id": null,
"conditions": [],
"data": null,
"citations": [],
"notes": null
},
{
"key": "density",
"unit": "g/l",
"value": 1.25,
"type": null,
"method": null,
"method_description": null,
"sample_preparation": null,
"uncertainty": null,
"uncertainty_type": null,
"components": [],
"components_relative": [],
"structure": null,
"set_id": null,

"conditions": [
{
"key": "temperature",
"unit": "degC",
"value": 4,
"type": null,
"uncertainty": null,
"uncertainty_type": null,
"material": null,
"descriptor": null,
"set_id": null,
"measurement_id": null,
"data": null
}
],
"data": null,
"citations": [],
"notes": null
},
{
"key": "+temp_boiling",
"unit": "K",
"value": 77.355,
"type": null,
"method": null,
"method_description": null,
"sample_preparation": null,
"uncertainty": null,
"uncertainty_type": null,
"components": [],
"components_relative": [],
"structure": null,
"set_id": null,
"conditions": [
{
"key": "pressure",
"unit": "atm",
"value": 1,
"type": null,
"uncertainty": null,
"uncertainty_type": null,
"material": null,
"descriptor": null,
"set_id": null,
"measurement_id": null,
"data": null

```
            }
          ],
          "data": null,
          "citations": [],
          "notes": null
        },
        {
          "key": "temp_melt",
          "unit": "K",
          "value": 63.23,
          "type": null,
          "method": null,
          "method_description": null,
          "sample_preparation": null,
          "uncertainty": null,
          "uncertainty_type": null,
          "components": [],
          "components_relative": [],
          "structure": null,
          "set_id": null,
          "conditions": [
            {
              "key": "pressure",
              "unit": "bar",
              "value": 1,
              "type": null,
              "uncertainty": null,
              "uncertainty_type": null,
              "material": null,
              "descriptor": null,
              "set_id": null,
              "measurement_id": null,
              "data": null
            }
          ],
          "data": null,
          "citations": [],
          "notes": null
        }
      ],
      "notes": null,
      "group": "local://cript/group/5a5a9fb2-44ca-41e7-931d-b544a9a02b86/",
      "model_version": "0.4.3"
    }
```

Argon


```
{
    "url": "local://cript/material/922c134c-99fc-4aac-ab22-4796dd3c6381/",
    "uid": "922c134c-99fc-4aac-ab22-4796dd3c6381",
    "public": false,
    "created_at": "2022-09-08T13:48:10.314309",
    "updated_at": "2022-09-08T13:48:10.314309",
    "project": "local://cript/project/ef26f322-8e03-4655-9aca-a731b6e5ae36/",
    "name": "Argon",
    "identifiers": [
        {
            "key": "preferred_name",
            "value": "argon"
        },
        {
            "key": "names",
            "value": [
                "Ar"
            ]
        },
        {
            "key": "cas",
            "value": "7440-37-1"
        },
        {
            "key": "smiles",
            "value": "Ar"
        },
        {
            "key": "chem_formula",
            "value": "Ar"
        },
        {
            "key": "pubchem_cid",
            "value": 23968
        },
        {
            "key": "inchi_key",
            "value": "XKRFYHLGVUSROY-UHFFFAOYSA-N"
        }
    ],
    "components": [],
    "keywords": [],
    "process": null,
    "properties": [
        {
```

"key": "phase",
"unit": null,
"value": "gas",
"type": null,
"method": null,
"method_description": null,
"sample_preparation": null,
"uncertainty": null,
"uncertainty_type": null,
"components": [],
"components_relative": [],
"structure": null,
"set_id": null,
"conditions": [],
"data": null,
"citations": [],
"notes": null
},
{
"key": "color",
"unit": null,
"value": "colorless",
"type": null,
"method": null,
"method_description": null,
"sample_preparation": null,
"uncertainty": null,
"uncertainty_type": null,
"components": [],
"components_relative": [],
"structure": null,
"set_id": null,
"conditions": [],
"data": null,
"citations": [],
"notes": null
},
{
"key": "molar_mass",
"unit": "g/mol",
"value": 39.95,
"type": null,
"method": "prescribed",
"method_description": null,
"sample_preparation": null,
"uncertainty": null,

"uncertainty_type": null,
"components": [],
"components_relative": [],
"structure": null,
"set_id": null,
"conditions": [],
"data": null,
"citations": [],
"notes": null
},
{
"key": "density",
"unit": "g/l",
"value": 1.784,
"type": null,
"method": null,
"method_description": null,
"sample_preparation": null,
"uncertainty": null,
"uncertainty_type": null,
"components": [],
"components_relative": [],
"structure": null,
"set_id": null,
"conditions": [
{
"key": "temperature",
"unit": "degC",
"value": 0,
"type": null,
"uncertainty": null,
"uncertainty_type": null,
"material": null,
"descriptor": null,
"set_id": null,
"measurement_id": null,
"data": null
},
{
"key": "pressure",
"unit": "bar",
"value": 1,
"type": null,
"uncertainty": null,
"uncertainty_type": null,
"material": null,

"descriptor": null,
"set_id": null,
"measurement_id": null,
"data": null
}
],
"data": null,
"citations": [],
"notes": null
},
{
"key": "+temp_boiling",
"unit": "K",
"value": 87.3,
"type": null,
"method": null,
"method_description": null,
"sample_preparation": null,
"uncertainty": null,
"uncertainty_type": null,
"components": [],
"components_relative": [],
"structure": null,
"set_id": null,
"conditions": [
{
"key": "pressure",
"unit": "atm",
"value": 1,
"type": null,
"uncertainty": null,
"uncertainty_type": null,
"material": null,
"descriptor": null,
"set_id": null,
"measurement_id": null,
"data": null
}
],
"data": null,
"citations": [],
"notes": null
},
{
"key": "temp_melt",
"unit": "K",

"value": 83.81,
"type": null,
"method": null,
"method_description": null,
"sample_preparation": null,
"uncertainty": null,
"uncertainty_type": null,
"components": [],
"components_relative": [],
"structure": null,
"set_id": null,
"conditions": [
{
"key": "pressure",
"unit": "bar",
"value": 1,
"type": null,
"uncertainty": null,
"uncertainty_type": null,
"material": null,
"descriptor": null,
"set_id": null,
"measurement_id": null,
"data": null
}
],
"data": null,
"citations": [],
"notes": null
}
],
"notes": null,
"group": "local://cript/group/5a5a9fb2-44ca-41e7-931d-b544a9a02b86/",
"model_version": "0.4.3"
}

Tetrahydrofuran

{
"url": "local://cript/material/46077c6a-1657-4d14-a8e5-645be25af625/",
"uid": "46077c6a-1657-4d14-a8e5-645be25af625",
"public": false,
"created_at": "2022-09-08T13:48:10.317328",
"updated_at": "2022-09-08T13:48:10.317328",
"project": "local://cript/project/ef26f322-8e03-4655-9aca-a731b6e5ae36/",
"name": "thf",
"identifiers": [

{
"key": "preferred_name",
"value": "tetrahydrofuran"
},
{
"key": "names",
"value": [
"oxolane",
"1,4-epoxybutane",
"oxacyclopentane",
"THF",
"butylene oxide",
"cyclotetramethylene oxide"
]
},
{
"key": "cas",
"value": "109-99-9"
},
{
"key": "smiles",
"value": "C1CCOC1"
},
{
"key": "chem_formula",
"value": "C4H8O"
},
{
"key": "pubchem_cid",
"value": 8028
},
{
"key": "inchi_key",
"value": "WYURNTSHIVDZCO-UHFFFAOYSA-N"
}
],
"components": [],
"keywords": [],
"process": null,
"properties": [
{
"key": "phase",
"unit": null,
"value": "liquid",
"type": null,
"method": null,

"method_description": null,
"sample_preparation": null,
"uncertainty": null,
"uncertainty_type": null,
"components": [],
"components_relative": [],
"structure": null,
"set_id": null,
"conditions": [],
"data": null,
"citations": [],
"notes": null
},
{
"key": "color",
"unit": null,
"value": "colorless",
"type": null,
"method": null,
"method_description": null,
"sample_preparation": null,
"uncertainty": null,
"uncertainty_type": null,
"components": [],
"components_relative": [],
"structure": null,
"set_id": null,
"conditions": [],
"data": null,
"citations": [],
"notes": null
},
{
"key": "molar_mass",
"unit": "g/mol",
"value": 72.107,
"type": null,
"method": "prescribed",
"method_description": null,
"sample_preparation": null,
"uncertainty": null,
"uncertainty_type": null,
"components": [],
"components_relative": [],
"structure": null,
"set_id": null,

"conditions": [],
"data": null,
"citations": [],
"notes": null
},
{
"key": "density",
"unit": "g/ml",
"value": 0.8876,
"type": null,
"method": null,
"method_description": null,
"sample_preparation": null,
"uncertainty": null,
"uncertainty_type": null,
"components": [],
"components_relative": [],
"structure": null,
"set_id": null,
"conditions": [
{
"key": "temperature",
"unit": "degC",
"value": 20,
"type": null,
"uncertainty": null,
"uncertainty_type": null,
"material": null,
"descriptor": null,
"set_id": null,
"measurement_id": null,
"data": null
}
],
"data": null,
"citations": [],
"notes": null
},
{
"key": "+temp_boiling",
"unit": "degC",
"value": 66,
"type": null,
"method": null,
"method_description": null,
"sample_preparation": null,

"uncertainty": null,
"uncertainty_type": null,
"components": [],
"components_relative": [],
"structure": null,
"set_id": null,
"conditions": [
{
"key": "pressure",
"unit": "atm",
"value": 1,
"type": null,
"uncertainty": null,
"uncertainty_type": null,
"material": null,
"descriptor": null,
"set_id": null,
"measurement_id": null,
"data": null
}
],
"data": null,
"citations": [],
"notes": null
},
{
"key": "temp_melt",
"unit": "degC",
"value": -108,
"type": null,
"method": null,
"method_description": null,
"sample_preparation": null,
"uncertainty": null,
"uncertainty_type": null,
"components": [],
"components_relative": [],
"structure": null,
"set_id": null,
"conditions": [
{
"key": "pressure",
"unit": "bar",
"value": 1,
"type": null,
"uncertainty": null,

```
            "uncertainty_type": null,
            "material": null,
            "descriptor": null,
            "set_id": null,
            "measurement_id": null,
            "data": null
        }
    ],
    "data": null,
    "citations": [],
    "notes": null
},
{
    "key": "+solubility",
    "unit": "g/ml",
    "value": -1,
    "type": null,
    "method": null,
    "method_description": null,
    "sample_preparation": null,
    "uncertainty": null,
    "uncertainty_type": null,
    "components": [],
    "components_relative": [],
    "structure": null,
    "set_id": null,
    "conditions": [
        {
            "key": "temperature",
            "unit": "degC",
            "value": 20,
            "type": null,
            "uncertainty": null,
            "uncertainty_type": null,
            "material": null,
            "descriptor": null,
            "set_id": null,
            "measurement_id": null,
            "data": null
        },
        {
            "key": "+solvent",
            "unit": null,
            "value": null,
            "type": null,
            "uncertainty": null,
```

"uncertainty_type": null,
"material": "local://cript/material/cf7d4d83-88d7-4a3a-9eec-5f707c806b3b/",
"descriptor": null,
"set_id": null,
"measurement_id": null,
"data": null
}
],
"data": null,
"citations": [],
"notes": null
}
],
"notes": null,
"group": "local://cript/group/5a5a9fb2-44ca-41e7-931d-b544a9a02b86/",
"model_version": "0.4.3"
}

Toluene

{
"url": "local://cript/material/63f071c2-c64a-4b71-a84b-8de5b5812f4c/",
"uid": "63f071c2-c64a-4b71-a84b-8de5b5812f4c",
"public": false,
"created_at": "2022-09-08T13:48:10.316309",
"updated_at": "2022-09-08T13:48:10.316309",
"project": "local://cript/project/ef26f322-8e03-4655-9aca-a731b6e5ae36/",
"name": "toluene",
"identifiers": [
{
"key": "preferred_name",
"value": "toluene"
},
{
"key": "names",
"value": [
"methylbenzene"
]
},
{
"key": "cas",
"value": "108-88-3"
},
{
"key": "smiles",
"value": "Cc1ccccc1"
},

{
"key": "chem_formula",
"value": "C7H8"
},
{
"key": "pubchem_cid",
"value": 1140
},
{
"key": "inchi_key",
"value": "YXFVVABEGXRONW-UHFFFAOYSA-N"
}
],
"components": [],
"keywords": [],
"process": null,
"properties": [
{
"key": "phase",
"unit": null,
"value": "liquid",
"type": null,
"method": null,
"method_description": null,
"sample_preparation": null,
"uncertainty": null,
"uncertainty_type": null,
"components": [],
"components_relative": [],
"structure": null,
"set_id": null,
"conditions": [],
"data": null,
"citations": [],
"notes": null
},
{
"key": "color",
"unit": null,
"value": "colorless",
"type": null,
"method": null,
"method_description": null,
"sample_preparation": null,
"uncertainty": null,
"uncertainty_type": null,

"components": [],
"components_relative": [],
"structure": null,
"set_id": null,
"conditions": [],
"data": null,
"citations": [],
"notes": null
},
{
"key": "molar_mass",
"unit": "g/mol",
"value": 92.141,
"type": null,
"method": "prescribed",
"method_description": null,
"sample_preparation": null,
"uncertainty": null,
"uncertainty_type": null,
"components": [],
"components_relative": [],
"structure": null,
"set_id": null,
"conditions": [],
"data": null,
"citations": [],
"notes": null
},
{
"key": "density",
"unit": "g/ml",
"value": 0.87,
"type": null,
"method": null,
"method_description": null,
"sample_preparation": null,
"uncertainty": null,
"uncertainty_type": null,
"components": [],
"components_relative": [],
"structure": null,
"set_id": null,
"conditions": [
{
"key": "temperature",
"unit": "degC",

"value": 25,
"type": null,
"uncertainty": null,
"uncertainty_type": null,
"material": null,
"descriptor": null,
"set_id": null,
"measurement_id": null,
"data": null
}
],
"data": null,
"citations": [],
"notes": null
},
{
"key": "+temp_boiling",
"unit": "degC",
"value": 111,
"type": null,
"method": null,
"method_description": null,
"sample_preparation": null,
"uncertainty": null,
"uncertainty_type": null,
"components": [],
"components_relative": [],
"structure": null,
"set_id": null,
"conditions": [
{
"key": "pressure",
"unit": "atm",
"value": 1,
"type": null,
"uncertainty": null,
"uncertainty_type": null,
"material": null,
"descriptor": null,
"set_id": null,
"measurement_id": null,
"data": null
}
],
"data": null,
"citations": [],

```
    "notes": null
  },
  {
    "key": "temp_melt",
    "unit": "degC",
    "value": -95,
    "type": null,
    "method": null,
    "method_description": null,
    "sample_preparation": null,
    "uncertainty": null,
    "uncertainty_type": null,
    "components": [],
    "components_relative": [],
    "structure": null,
    "set_id": null,
    "conditions": [
      {
        "key": "pressure",
        "unit": "bar",
        "value": 1,
        "type": null,
        "uncertainty": null,
        "uncertainty_type": null,
        "material": null,
        "descriptor": null,
        "set_id": null,
        "measurement_id": null,
        "data": null
      }
    ],
    "data": null,
    "citations": [],
    "notes": null
  },
  {
    "key": "solubility",
    "unit": "g/L",
    "value": 0.52,
    "type": null,
    "method": null,
    "method_description": null,
    "sample_preparation": null,
    "uncertainty": null,
    "uncertainty_type": null,
    "components": [],
```

"components_relative": [],
"structure": null,
"set_id": null,
"conditions": [
{
"key": "temperature",
"unit": "degC",
"value": 20,
"type": null,
"uncertainty": null,
"uncertainty_type": null,
"material": null,
"descriptor": null,
"set_id": null,
"measurement_id": null,
"data": null
}
],
"data": null,
"citations": [],
"notes": null
}
],
"notes": null,
"group": "local://cript/group/5a5a9fb2-44ca-41e7-931d-b544a9a02b86/",
"model_version": "0.4.3"
}

Water

{
"url": "local://cript/material/cf7d4d83-88d7-4a3a-9eec-5f707c806b3b/",
"uid": "cf7d4d83-88d7-4a3a-9eec-5f707c806b3b",
"public": false,
"created_at": "2022-09-08T13:48:10.311309",
"updated_at": "2022-09-08T13:48:10.311309",
"project": "local://cript/project/ef26f322-8e03-4655-9aca-a731b6e5ae36/",
"name": "water",
"identifiers": [
{
"key": "preferred_name",
"value": "water"
},
{
"key": "names",
"value": [
"h2o",

"dihydrogen oxide"
]
},
{
"key": "cas",
"value": "7732-18-5"
},
{
"key": "smiles",
"value": "O"
},
{
"key": "chem_formula",
"value": "H2O"
},
{
"key": "pubchem_cid",
"value": 962
},
{
"key": "inchi_key",
"value": "XLYOFNOQVPJJNP-UHFFFAOYSA-N"
}
],
"components": [],
"keywords": [],
"process": null,
"properties": [
{
"key": "phase",
"unit": null,
"value": "liquid",
"type": null,
"method": null,
"method_description": null,
"sample_preparation": null,
"uncertainty": null,
"uncertainty_type": null,
"components": [],
"components_relative": [],
"structure": null,
"set_id": null,
"conditions": [],
"data": null,
"citations": [],
"notes": null

},
{
"key": "color",
"unit": null,
"value": "colorless",
"type": null,
"method": null,
"method_description": null,
"sample_preparation": null,
"uncertainty": null,
"uncertainty_type": null,
"components": [],
"components_relative": [],
"structure": null,
"set_id": null,
"conditions": [],
"data": null,
"citations": [],
"notes": null
},
{
"key": "molar_mass",
"unit": "g/mol",
"value": 18.015,
"type": null,
"method": "prescribed",
"method_description": null,
"sample_preparation": null,
"uncertainty": null,
"uncertainty_type": null,
"components": [],
"components_relative": [],
"structure": null,
"set_id": null,
"conditions": [],
"data": null,
"citations": [],
"notes": null
},
{
"key": "density",
"unit": "g/ml",
"value": 1.0,
"type": null,
"method": null,
"method_description": null,

"sample_preparation": null,
"uncertainty": null,
"uncertainty_type": null,
"components": [],
"components_relative": [],
"structure": null,
"set_id": null,
"conditions": [
{
"key": "temperature",
"unit": "degC",
"value": 4,
"type": null,
"uncertainty": null,
"uncertainty_type": null,
"material": null,
"descriptor": null,
"set_id": null,
"measurement_id": null,
"data": null
}
],
"data": null,
"citations": [],
"notes": null
},
{
"key": "+temp_boiling",
"unit": "degC",
"value": 100,
"type": null,
"method": null,
"method_description": null,
"sample_preparation": null,
"uncertainty": null,
"uncertainty_type": null,
"components": [],
"components_relative": [],
"structure": null,
"set_id": null,
"conditions": [
{
"key": "pressure",
"unit": "atm",
"value": 1,
"type": null,

"uncertainty": null,
"uncertainty_type": null,
"material": null,
"descriptor": null,
"set_id": null,
"measurement_id": null,
"data": null
}
],
"data": null,
"citations": [],
"notes": null
},
{
"key": "temp_melt",
"unit": "degC",
"value": 0,
"type": null,
"method": null,
"method_description": null,
"sample_preparation": null,
"uncertainty": null,
"uncertainty_type": null,
"components": [],
"components_relative": [],
"structure": null,
"set_id": null,
"conditions": [
{
"key": "pressure",
"unit": "bar",
"value": 1,
"type": null,
"uncertainty": null,
"uncertainty_type": null,
"material": null,
"descriptor": null,
"set_id": null,
"measurement_id": null,
"data": null
}
],
"data": null,
"citations": [],
"notes": null
}

],
"notes": null,
"group": "local://cript/group/5a5a9fb2-44ca-41e7-931d-b544a9a02b86/",
"model_version": "0.4.3"
}

Methanol

{
"url": "local://cript/material/df9e5147-a5cc-49e1-b11a-23dcc7263d3c/",
"uid": "df9e5147-a5cc-49e1-b11a-23dcc7263d3c",
"public": false,
"created_at": "2022-09-08T13:48:10.320309",
"updated_at": "2022-09-08T13:48:10.320309",
"project": "local://cript/project/ef26f322-8e03-4655-9aca-a731b6e5ae36/",
"name": "methanol",
"identifiers": [
{
"key": "preferred_name",
"value": "methanol"
},
{
"key": "names",
"value": [
"Methyl alcohol",
"CH3OH",
"MeOH"
]
},
{
"key": "cas",
"value": "67-56-1"
},
{
"key": "smiles",
"value": "CO"
},
{
"key": "chem_formula",
"value": "CH4O"
},
{
"key": "pubchem_cid",
"value": 887
},
{
"key": "inchi_key",

"value": "OKKJLVBELUTLKV-UHFFFAOYSA-N"
}
],
"components": [],
"keywords": [],
"process": null,
"properties": [
{
"key": "phase",
"unit": null,
"value": "liquid",
"type": null,
"method": null,
"method_description": null,
"sample_preparation": null,
"uncertainty": null,
"uncertainty_type": null,
"components": [],
"components_relative": [],
"structure": null,
"set_id": null,
"conditions": [],
"data": null,
"citations": [],
"notes": null
},
{
"key": "color",
"unit": null,
"value": "colorless",
"type": null,
"method": null,
"method_description": null,
"sample_preparation": null,
"uncertainty": null,
"uncertainty_type": null,
"components": [],
"components_relative": [],
"structure": null,
"set_id": null,
"conditions": [],
"data": null,
"citations": [],
"notes": null
},
{

"key": "molar_mass",
"unit": "g/mol",
"value": 32.0,
"type": null,
"method": "prescribed",
"method_description": null,
"sample_preparation": null,
"uncertainty": null,
"uncertainty_type": null,
"components": [],
"components_relative": [],
"structure": null,
"set_id": null,
"conditions": [],
"data": null,
"citations": [],
"notes": null
},
{
"key": "density",
"unit": "g/ml",
"value": 0.792,
"type": null,
"method": null,
"method_description": null,
"sample_preparation": null,
"uncertainty": null,
"uncertainty_type": null,
"components": [],
"components_relative": [],
"structure": null,
"set_id": null,
"conditions": [
{
"key": "temperature",
"unit": "degC",
"value": 20,
"type": null,
"uncertainty": null,
"uncertainty_type": null,
"material": null,
"descriptor": null,
"set_id": null,
"measurement_id": null,
"data": null
}

],
"data": null,
"citations": [],
"notes": null
},
{
"key": "+temp_boiling",
"unit": "degC",
"value": 64.7,
"type": null,
"method": null,
"method_description": null,
"sample_preparation": null,
"uncertainty": null,
"uncertainty_type": null,
"components": [],
"components_relative": [],
"structure": null,
"set_id": null,
"conditions": [
{
"key": "pressure",
"unit": "atm",
"value": 1,
"type": null,
"uncertainty": null,
"uncertainty_type": null,
"material": null,
"descriptor": null,
"set_id": null,
"measurement_id": null,
"data": null
}
],
"data": null,
"citations": [],
"notes": null
},
{
"key": "temp_melt",
"unit": "degC",
"value": -97.6,
"type": null,
"method": null,
"method_description": null,
"sample_preparation": null,

"uncertainty": null,
"uncertainty_type": null,
"components": [],
"components_relative": [],
"structure": null,
"set_id": null,
"conditions": [
{
"key": "pressure",
"unit": "bar",
"value": 1,
"type": null,
"uncertainty": null,
"uncertainty_type": null,
"material": null,
"descriptor": null,
"set_id": null,
"measurement_id": null,
"data": null
}
],
"data": null,
"citations": [],
"notes": null
}
],
"notes": null,
"group": "local://cript/group/5a5a9fb2-44ca-41e7-931d-b544a9a02b86/",
"model_version": "0.4.3"
}

Styrene

{
"url": "local://cript/material/c46b68d8-b521-4851-b85e-632ad3d02e96/",
"uid": "c46b68d8-b521-4851-b85e-632ad3d02e96",
"public": false,
"created_at": "2022-09-08T13:48:10.315309",
"updated_at": "2022-09-08T13:48:10.315309",
"project": "local://cript/project/ef26f322-8e03-4655-9aca-a731b6e5ae36/",
"name": "styrene",
"identifiers": [
{
"key": "preferred_name",
"value": "styrene"
},
{

"key": "names",
"value": [
"vinylbenzene",
"phenylethylene",
"ethenylbenzene"
]
},
{
"key": "cas",
"value": "100-42-5"
},
{
"key": "smiles",
"value": "C=Cc1ccccc1"
},
{
"key": "chem_formula",
"value": "C8H8"
},
{
"key": "pubchem_cid",
"value": 7501
},
{
"key": "inchi_key",
"value": "PPBRXRYQALVLMV-UHFFFAOYSA-N"
}
],
"components": [],
"keywords": [
"styrene"
],
"process": null,
"properties": [
{
"key": "phase",
"unit": null,
"value": "liquid",
"type": null,
"method": null,
"method_description": null,
"sample_preparation": null,
"uncertainty": null,
"uncertainty_type": null,
"components": [],
"components_relative": [],

"structure": null,
"set_id": null,
"conditions": [],
"data": null,
"citations": [],
"notes": null
},
{
"key": "color",
"unit": null,
"value": "colorless",
"type": null,
"method": null,
"method_description": null,
"sample_preparation": null,
"uncertainty": null,
"uncertainty_type": null,
"components": [],
"components_relative": [],
"structure": null,
"set_id": null,
"conditions": [],
"data": null,
"citations": [],
"notes": null
},
{
"key": "molar_mass",
"unit": "g/mol",
"value": 104.15,
"type": null,
"method": "prescribed",
"method_description": null,
"sample_preparation": null,
"uncertainty": null,
"uncertainty_type": null,
"components": [],
"components_relative": [],
"structure": null,
"set_id": null,
"conditions": [],
"data": null,
"citations": [],
"notes": null
},
{

"key": "density",
"unit": "g/ml",
"value": 0.906,
"type": null,
"method": null,
"method_description": null,
"sample_preparation": null,
"uncertainty": null,
"uncertainty_type": null,
"components": [],
"components_relative": [],
"structure": null,
"set_id": null,
"conditions": [
{
"key": "temperature",
"unit": "degC",
"value": 25,
"type": null,
"uncertainty": null,
"uncertainty_type": null,
"material": null,
"descriptor": null,
"set_id": null,
"measurement_id": null,
"data": null
}
],
"data": null,
"citations": [],
"notes": null
},
{
"key": "+temp_boiling",
"unit": "degC",
"value": 145,
"type": null,
"method": null,
"method_description": null,
"sample_preparation": null,
"uncertainty": null,
"uncertainty_type": null,
"components": [],
"components_relative": [],
"structure": null,
"set_id": null,

"conditions": [
{
"key": "pressure",
"unit": "atm",
"value": 1,
"type": null,
"uncertainty": null,
"uncertainty_type": null,
"material": null,
"descriptor": null,
"set_id": null,
"measurement_id": null,
"data": null
}
],
"data": null,
"citations": [],
"notes": null
},
{
"key": "temp_melt",
"unit": "degC",
"value": -30,
"type": null,
"method": null,
"method_description": null,
"sample_preparation": null,
"uncertainty": null,
"uncertainty_type": null,
"components": [],
"components_relative": [],
"structure": null,
"set_id": null,
"conditions": [
{
"key": "pressure",
"unit": "bar",
"value": 1,
"type": null,
"uncertainty": null,
"uncertainty_type": null,
"material": null,
"descriptor": null,
"set_id": null,
"measurement_id": null,
"data": null

```
            }
        ],
        "data": null,
        "citations": [],
        "notes": null
    }
  ],
  "notes": null,
  "model_version": "0.4.3"
}
```


1-Butanol


```
{
  "url": "local://cript/material/b719064a-0ece-4990-a0eb-f0e0fffab1e3/",
  "uid": "b719064a-0ece-4990-a0eb-f0e0fffab1e3",
  "public": false,
  "created_at": "2022-09-08T13:48:10.319308",
  "updated_at": "2022-09-08T13:48:10.319308",
  "project": "local://cript/project/ef26f322-8e03-4655-9aca-a731b6e5ae36/",
  "name": "nbutanol",
  "identifiers": [
    {
      "key": "preferred_name",
      "value": "1-butanol"
    },
    {
      "key": "names",
      "value": [
        "n-butanol",
        "n-butyl alcohol",
        "1-butyl alcohol",
        "nBuOH"
      ]
    },
    {
      "key": "cas",
      "value": "71-36-3"
    },
    {
      "key": "smiles",
      "value": "OCCCC"
    },
    {
      "key": "chem_formula",
      "value": "C4H10O"
    },
```

{
"key": "pubchem_cid",
"value": 263
},
{
"key": "inchi_key",
"value": "LRHPLDYGYMQRHN-UHFFFAOYSA-N"
}
],
"components": [],
"keywords": [],
"process": null,
"properties": [
{
"key": "phase",
"unit": null,
"value": "liquid",
"type": null,
"method": null,
"method_description": null,
"sample_preparation": null,
"uncertainty": null,
"uncertainty_type": null,
"components": [],
"components_relative": [],
"structure": null,
"set_id": null,
"conditions": [],
"data": null,
"citations": [],
"notes": null
},
{
"key": "color",
"unit": null,
"value": "colorless",
"type": null,
"method": null,
"method_description": null,
"sample_preparation": null,
"uncertainty": null,
"uncertainty_type": null,
"components": [],
"components_relative": [],
"structure": null,
"set_id": null,

"conditions": [],
"data": null,
"citations": [],
"notes": null
},
{
"key": "molar_mass",
"unit": "g/mol",
"value": 74.123,
"type": null,
"method": "prescribed",
"method_description": null,
"sample_preparation": null,
"uncertainty": null,
"uncertainty_type": null,
"components": [],
"components_relative": [],
"structure": null,
"set_id": null,
"conditions": [],
"data": null,
"citations": [],
"notes": null
},
{
"key": "density",
"unit": "g/ml",
"value": 0.81,
"type": null,
"method": null,
"method_description": null,
"sample_preparation": null,
"uncertainty": null,
"uncertainty_type": null,
"components": [],
"components_relative": [],
"structure": null,
"set_id": null,
"conditions": [
{
"key": "temperature",
"unit": "degC",
"value": 20,
"type": null,
"uncertainty": null,
"uncertainty_type": null,

"material": null,
"descriptor": null,
"set_id": null,
"measurement_id": null,
"data": null
}
],
"data": null,
"citations": [],
"notes": null
},
{
"key": "+temp_boiling",
"unit": "degC",
"value": 117.7,
"type": null,
"method": null,
"method_description": null,
"sample_preparation": null,
"uncertainty": null,
"uncertainty_type": null,
"components": [],
"components_relative": [],
"structure": null,
"set_id": null,
"conditions": [
{
"key": "pressure",
"unit": "atm",
"value": 1,
"type": null,
"uncertainty": null,
"uncertainty_type": null,
"material": null,
"descriptor": null,
"set_id": null,
"measurement_id": null,
"data": null
}
],
"data": null,
"citations": [],
"notes": null
},
{
"key": "temp_melt",

"unit": "degC",
"value": -89.9,
"type": null,
"method": null,
"method_description": null,
"sample_preparation": null,
"uncertainty": null,
"uncertainty_type": null,
"components": [],
"components_relative": [],
"structure": null,
"set_id": null,
"conditions": [
{
"key": "pressure",
"unit": "bar",
"value": 1,
"type": null,
"uncertainty": null,
"uncertainty_type": null,
"material": null,
"descriptor": null,
"set_id": null,
"measurement_id": null,
"data": null
}
],
"data": null,
"citations": [],
"notes": null
}
],
"notes": null,
"group": "local://cript/group/5a5a9fb2-44ca-41e7-931d-b544a9a02b86/",
"model_version": "0.4.3"
}

Sec-Butyl Lithium

{
"url": "local://cript/material/89a49359-bb0a-40ba-8b0e-c4fbc02c7907/",
"uid": "89a49359-bb0a-40ba-8b0e-c4fbc02c7907",
"public": false,
"created_at": "2022-09-08T13:48:10.321309",
"updated_at": "2022-09-08T13:48:10.321309",
"project": "local://cript/project/ef26f322-8e03-4655-9aca-a731b6e5ae36/",
"name": "secBuLi",

"identifiers": [
{
"key": "preferred_name",
"value": "sec-butyllithium"
},
{
"key": "names",
"value": [
"butan-2-yllithium",
"sBuLi",
"secBuLi",
"s-butyllithium"
]
},
{
"key": "cas",
"value": "598-30-1"
},
{
"key": "smiles",
"value": "[Li]C(C)CC"
},
{
"key": "chem_formula",
"value": "C4H9Li"
},
{
"key": "pubchem_cid",
"value": 887
},
{
"key": "inchi_key",
"value": "VATDYQWILMGLEW-UHFFFAOYSA-N"
}
],
"components": [],
"keywords": [],
"process": null,
"properties": [
{
"key": "molar_mass",
"unit": "g/mol",
"value": 64.06,
"type": null,
"method": "prescribed",
"method_description": null,

"sample_preparation": null,
"uncertainty": null,
"uncertainty_type": null,
"components": [],
"components_relative": [],
"structure": null,
"set_id": null,
"conditions": [],
"data": null,
"citations": [],
"notes": null
}
],
"notes": null,
"group": "local://cript/group/5a5a9fb2-44ca-41e7-931d-b544a9a02b86/",
"model_version": "0.4.3"
}

Sec-Butyl Lithium Solution

{
"url": "local://cript/material/3bc36780-f200-4954-9d58-2108e30c83a9/",
"uid": "3bc36780-f200-4954-9d58-2108e30c83a9",
"public": false,
"created_at": "2022-09-08T13:48:10.322309",
"updated_at": "2022-09-08T13:48:10.322309",
"project": "local://cript/project/ef26f322-8e03-4655-9aca-a731b6e5ae36/",
"name": "SecBuLi solution 1.4M cHex",
"identifiers": [
{
"key": "preferred_name",
"value": "sec-butyllithium"
},
{
"key": "names",
"value": [
"butan-2-yllithium",
"sBuLi",
"secBuLi",
"s-butyllithium"
]
},
{
"key": "cas",
"value": "598-30-1"
},
{

"key": "smiles",
"value": "[Li]C(C)CC"
},
{
"key": "chem_formula",
"value": "C4H9Li"
},
{
"key": "pubchem_cid",
"value": 887
},
{
"key": "inchi_key",
"value": "VATDYQWILMGLEW-UHFFFAOYSA-N"
}
],
"components": [
"local://cript/material/89a49359-bb0a-40ba-8b0e-c4fbc02c7907/",
"local://cript/material/63f071c2-c64a-4b71-a84b-8de5b5812f4c/"
],
"keywords": [],
"process": null,
"properties": [
{
"key": "phase",
"unit": null,
"value": "solution",
"type": null,
"method": null,
"method_description": null,
"sample_preparation": null,
"uncertainty": null,
"uncertainty_type": null,
"components": [],
"components_relative": [],
"structure": null,
"set_id": null,
"conditions": [],
"data": null,
"citations": [],
"notes": null
},
{
"key": "density",
"unit": "g/ml",
"value": 0.769,

"type": null,
"method": null,
"method_description": null,
"sample_preparation": null,
"uncertainty": null,
"uncertainty_type": null,
"components": [],
"components_relative": [],
"structure": null,
"set_id": null,
"conditions": [
{
"key": "pressure",
"unit": "bar",
"value": 1,
"type": null,
"uncertainty": null,
"uncertainty_type": null,
"material": null,
"descriptor": null,
"set_id": null,
"measurement_id": null,
"data": null
}
],
"data": null,
"citations": [],
"notes": null
},
{
"key": "conc_molar",
"unit": "M",
"value": 1.4,
"type": null,
"method": null,
"method_description": null,
"sample_preparation": null,
"uncertainty": null,
"uncertainty_type": null,
"components": [
"local://cript/material/89a49359-bb0a-40ba-8b0e-c4fbc02c7907/"
],
"components_relative": [
"local://cript/material/63f071c2-c64a-4b71-a84b-8de5b5812f4c/"
],
"structure": null,

      "set_id": null,
      "conditions": [],
      "data": null,
      "citations": [],
      "notes": null
    }
  ],
  "notes": null,
  "group": "local://cript/group/5a5a9fb2-44ca-41e7-931d-b544a9a02b86/",
  "model_version": "0.4.3"
}

Poly(styrene)

{
  "url": "local://cript/material/eaa94529-623a-4e43-a094-4205fc7bb909/",
  "uid": "eaa94529-623a-4e43-a094-4205fc7bb909",
  "public": false,
  "created_at": "2022-09-08T13:48:10.489312",
  "updated_at": "2022-09-08T13:48:10.489312",
  "project": "local://cript/project/ef26f322-8e03-4655-9aca-a731b6e5ae36/",
  "name": "polystyrene",
  "identifiers": [
    {
      "key": "preferred_name",
      "value": "polystyrene"
    },
    {
      "key": "names",
      "value": [
        "poly(styrene)",
        "poly(vinylbenzene)"
      ]
    },
    {
      "key": "chem_repeat",
      "value": "C8H8"
    },
    {
      "key": "bigsmiles",
      "value": "[H]{[>][<]C(C[>])c1ccccc1[<]}C(C)CC"
    },
    {
      "key": "cas",
      "value": "100-42-5"
    }
  ],

"components": [],
"keywords": [],
"process": "local://cript/process/ea11ef0e-cb4c-4ecb-91f1-ca7674ab103a/",
"properties": [
{
"key": "phase",
"unit": null,
"value": "solid",
"type": null,
"method": null,
"method_description": null,
"sample_preparation": null,
"uncertainty": null,
"uncertainty_type": null,
"components": [],
"components_relative": [],
"structure": null,
"set_id": null,
"conditions": [],
"data": null,
"citations": [],
"notes": null
},
{
"key": "color",
"unit": null,
"value": "white",
"type": null,
"method": null,
"method_description": null,
"sample_preparation": null,
"uncertainty": null,
"uncertainty_type": null,
"components": [],
"components_relative": [],
"structure": null,
"set_id": null,
"conditions": [],
"data": null,
"citations": [],
"notes": null
},
{
"key": "mw_n",
"unit": "g/mol",
"value": 4800,

"type": null,
"method": "nmr",
"method_description": null,
"sample_preparation": null,
"uncertainty": 400,
"uncertainty_type": null,
"components": [],
"components_relative": [],
"structure": null,
"set_id": null,
"conditions": [],
"data": "local://cript/data/b9d05e72-b095-467f-9853-42bb9284ae46/",
"citations": [],
"notes": null
},
{
"key": "mw_n",
"unit": "g/mol",
"value": 5200,
"type": null,
"method": "sec",
"method_description": null,
"sample_preparation": null,
"uncertainty": 100,
"uncertainty_type": null,
"components": [],
"components_relative": [],
"structure": null,
"set_id": null,
"conditions": [],
"data": "local://cript/data/b767d542-d319-4040-a028-934f099441e9/",
"citations": [],
"notes": null
},
{
"key": "mw_d",
"unit": null,
"value": 1.03,
"type": null,
"method": "sec",
"method_description": null,
"sample_preparation": null,
"uncertainty": 0.02,
"uncertainty_type": null,
"components": [],
"components_relative": [],

"structure": null,
"set_id": null,
"conditions": [],
"data": "local://cript/data/b767d542-d319-4040-a028-934f099441e9/",
"citations": [],
"notes": null
}
],
"notes": null,
"group": "local://cript/group/5a5a9fb2-44ca-41e7-931d-b544a9a02b86/",
"model_version": "0.4.3"
}

Deuterated Chloroform

{
"url": "local://cript/material/f8f76b12-12f3-4bf2-880d-75f13535e994/",
"uid": "f8f76b12-12f3-4bf2-880d-75f13535e994",
"public": false,
"created_at": "2022-09-08T13:48:10.312309",
"updated_at": "2022-09-08T13:48:10.312309",
"project": "local://cript/project/ef26f322-8e03-4655-9aca-a731b6e5ae36/",
"name": "deuterated chloroform",
"identifiers": [
{
"key": "preferred_name",
"value": "deuterated chloroform"
},
{
"key": "names",
"value": [
"chloroform-d",
"deuterochloroform",
"trichloro(deuterio)methane"
]
},
{
"key": "cas",
"value": "865-49-6"
},
{
"key": "smiles",
"value": "[2H]C(Cl)(Cl)Cl"
},
{
"key": "chem_formula",
"value": "CDCl3"

},
{
"key": "pubchem_cid",
"value": 71583
},
{
"key": "inchi_key",
"value": "HEDRZPFGACZZDS-MICDWDOJSA-N"
}
],
"components": [],
"keywords": [],
"process": null,
"properties": [
{
"key": "phase",
"unit": null,
"value": "liquid",
"type": null,
"method": null,
"method_description": null,
"sample_preparation": null,
"uncertainty": null,
"uncertainty_type": null,
"components": [],
"components_relative": [],
"structure": null,
"set_id": null,
"conditions": [],
"data": null,
"citations": [],
"notes": null
},
{
"key": "color",
"unit": null,
"value": "colorless",
"type": null,
"method": null,
"method_description": null,
"sample_preparation": null,
"uncertainty": null,
"uncertainty_type": null,
"components": [],
"components_relative": [],
"structure": null,

"set_id": null,
"conditions": [],
"data": null,
"citations": [],
"notes": null
},
{
"key": "molar_mass",
"unit": "g/mol",
"value": 120.384,
"type": null,
"method": "prescribed",
"method_description": null,
"sample_preparation": null,
"uncertainty": null,
"uncertainty_type": null,
"components": [],
"components_relative": [],
"structure": null,
"set_id": null,
"conditions": [],
"data": null,
"citations": [],
"notes": null
},
{
"key": "density",
"unit": "g/ml",
"value": 1.5,
"type": null,
"method": null,
"method_description": null,
"sample_preparation": null,
"uncertainty": null,
"uncertainty_type": null,
"components": [],
"components_relative": [],
"structure": null,
"set_id": null,
"conditions": [
{
"key": "temperature",
"unit": "degC",
"value": 25,
"type": null,
"uncertainty": null,

"uncertainty_type": null,
"material": null,
"descriptor": null,
"set_id": null,
"measurement_id": null,
"data": null
}
],
"data": null,
"citations": [],
"notes": null
},
{
"key": "+temp_boiling",
"unit": "degC",
"value": 61,
"type": null,
"method": null,
"method_description": null,
"sample_preparation": null,
"uncertainty": null,
"uncertainty_type": null,
"components": [],
"components_relative": [],
"structure": null,
"set_id": null,
"conditions": [
{
"key": "pressure",
"unit": "atm",
"value": 1,
"type": null,
"uncertainty": null,
"uncertainty_type": null,
"material": null,
"descriptor": null,
"set_id": null,
"measurement_id": null,
"data": null
}
],
"data": null,
"citations": [],
"notes": null
},
{

"key": "temp_melt",
"unit": "degC",
"value": -64,
"type": null,
"method": null,
"method_description": null,
"sample_preparation": null,
"uncertainty": null,
"uncertainty_type": null,
"components": [],
"components_relative": [],
"structure": null,
"set_id": null,
"conditions": [
{
"key": "pressure",
"unit": "bar",
"value": 1,
"type": null,
"uncertainty": null,
"uncertainty_type": null,
"material": null,
"descriptor": null,
"set_id": null,
"measurement_id": null,
"data": null
}
],
"data": null,
"citations": [],
"notes": null
}
],
"notes": null,
"group": "local://cript/group/5a5a9fb2-44ca-41e7-931d-b544a9a02b86/",
"model_version": "0.4.3"
}

PROCESS

{
"url": "local://cript/process/ea11ef0e-cb4c-4ecb-91f1-ca7674ab103a/",
"uid": "ea11ef0e-cb4c-4ecb-91f1-ca7674ab103a",
"public": false,
"created_at": "2022-09-08T13:48:10.407365",
"updated_at": "2022-09-08T13:48:10.407365",
"experiment": "local://cript/experiment/37422b5c-fa9e-4377-b3de-18ef3105f0f4/",

"name": "Anionic of Styrene",
"type": "multistep",
"keywords": [
"polymerization",
"living_poly",
"anionic",
"solution"
],
"description": "In an argon filled glovebox, a round bottom flask was filled with 216 ml of dried toluene. The solution of secBuLi (3 ml, 3.9 mmol) was added next, followed by styrene (22.3 g, 176 mmol) to initiate the polymerization. The reaction mixture immediately turned orange. After 30 min, the reaction was quenched with the addition of 3 ml of methanol. The polymer was isolated by precipitation in methanol 3 times and dried under vacuum.",
"prerequisite_processes": [],
"ingredients": [
{
"material": "local://cript/material/3bc36780-f200-4954-9d58-2108e30c83a9/",
"keyword": "initiator",
"quantities": [
{
"key": "volume",
"unit": "ml",
"value": 1,
"uncertainty": null,
"uncertainty_type": null
}
]
},
{
"material": "local://cript/material/63f071c2-c64a-4b71-a84b-8de5b5812f4c/",
"keyword": "solvent",
"quantities": [
{
"key": "volume",
"unit": "ml",
"value": 10,
"uncertainty": null,
"uncertainty_type": null
}
]
},
{
"material": "local://cript/material/c46b68d8-b521-4851-b85e-632ad3d02e96/",
"keyword": "monomer",
"quantities": [
{

"key": "mass",
"unit": "g",
"value": 0.455,
"uncertainty": null,
"uncertainty_type": null
}
]
},
{
"material": "local://cript/material/df9e5147-a5cc-49e1-b11a-23dcc7263d3c/",
"keyword": "workup",
"quantities": [
{
"key": "volume",
"unit": "ml",
"value": 0.1,
"uncertainty": null,
"uncertainty_type": null
}
]
},
{
"material": "local://cript/material/df9e5147-a5cc-49e1-b11a-23dcc7263d3c/",
"keyword": "workup",
"quantities": [
{
"key": "volume",
"unit": "ml",
"value": 0.1,
"uncertainty": null,
"uncertainty_type": null
}
]
},
{
"material": "local://cript/material/df9e5147-a5cc-49e1-b11a-23dcc7263d3c/",
"keyword": "quench",
"quantities": [
{
"key": "volume",
"unit": "ml",
"value": 100,
"uncertainty": null,
"uncertainty_type": null
}
]

    }
],
"equipment": [],
"properties": [
    {
        "key": "yield_mass",
        "unit": "g",
        "value": 0.47,
        "type": null,
        "method": "scale",
        "method_description": null,
        "sample_preparation": null,
        "uncertainty": 0.02,
        "uncertainty_type": "stdev",
        "components": [],
        "components_relative": [],
        "structure": null,
        "set_id": null,
        "conditions": [],
        "data": null,
        "citations": [],
        "notes": null
    }
],
"conditions": [
    {
        "key": "temperature",
        "unit": "degC",
        "value": 25,
        "type": null,
        "uncertainty": null,
        "uncertainty_type": null,
        "material": null,
        "descriptor": null,
        "set_id": null,
        "measurement_id": null,
        "data": null
    },
    {
        "key": "time_duration",
        "unit": "min",
        "value": 60,
        "type": null,
        "uncertainty": null,
        "uncertainty_type": null,
        "material": null,

"descriptor": null,
"set_id": null,
"measurement_id": null,
"data": null
},
{
"key": "atm",
"unit": null,
"value": "none",
"type": null,
"uncertainty": null,
"uncertainty_type": null,
"material": "local://cript/material/922c134c-99fc-4aac-ab22-4796dd3c6381/",
"descriptor": null,
"set_id": null,
"measurement_id": null,
"data": null
}
],
"set_id": null,
"products": [],
"waste": [],
"citations": [
{
"reference": "local://cript/reference/c8f4cd85-b717-4945-b862-319d37930705/",
"type": "reference",
"notes": null
}
],
"citations": [],
"notes": null,
"group": "local://cript/group/5a5a9fb2-44ca-41e7-931d-b544a9a02b86/",
"model_version": "0.4.3"
}

DATA

^{1}H Nuclear Magnetic Resonance Spectrum

{
"url": "local://cript/data/b9d05e72-b095-467f-9853-42bb9284ae46/",
"uid": "b9d05e72-b095-467f-9853-42bb9284ae46",
"public": false,
"created_at": "2022-09-08T13:48:10.426312",
"updated_at": "2022-09-08T13:48:10.426312",
"experiment": "local://cript/experiment/37422b5c-fa9e-4377-b3de-18ef3105f0f4/",
"name": "Crude 1H NMR of polystyrene",

"files": null,
"type": "nmr_h1",
"sample_preparation": null,
"calibration": null,
"configuration": null,
"materials": [],
"processes": [],
"citations": [],
"notes": null,
"group": "local://cript/group/5a5a9fb2-44ca-41e7-931d-b544a9a02b86/",
"model_version": "0.4.3"
}

Size Exclusion Chromatography Trace

{
"url": "local://cript/data/b767d542-d319-4040-a028-934f099441e9/",
"uid": "b767d542-d319-4040-a028-934f099441e9",
"public": false,
"created_at": "2022-09-08T13:48:10.414384",
"updated_at": "2022-09-08T13:48:10.434304",
"experiment": "local://cript/experiment/37422b5c-fa9e-4377-b3de-18ef3105f0f4/",
"name": "Crude SEC of polystyrene",
"files": null,
"type": "sec_trace",
"sample_preparation": null,
"calibration": null,
"configuration": null,
"materials": [],
"processes": [],
"citations": [],
"notes": null,
"group": "local://cript/group/5a5a9fb2-44ca-41e7-931d-b544a9a02b86/",
"model_version": "0.4.3",
"file": [
"local://cript/file/235bead1-2729-46a8-a5ea-d9b852b72c76/"
]
}

FILE

^{1}H Nuclear Magnetic Resonance Spectrum

{
"url": "local://cript/file/235bead1-2729-46a8-a5ea-d9b852b72c76/",
"uid": "235bead1-2729-46a8-a5ea-d9b852b72c76",
"public": false,
"created_at": "2022-09-08T13:48:10.431312",

"updated_at": "2022-09-08T13:48:10.431312",
"project": "local://cript/project/ef26f322-8e03-4655-9aca-a731b6e5ae36/",
"data": [
"local://cript/data/b9d05e72-b095-467f-9853-42bb9284ae46/"
],
"type": "data",
"name": "nmr.txt",
"checksum": "f30de031a83357db695b3723d91c8ecfbaf9d9132b53b91811e35867365a35ce",
"unique_name": null,
"extension": ".txt",
"source": "C:/Users/nicep/Desktop/pyth_proj/cript/examples/test_data/nmr.txt",
"group": "local://cript/group/5a5a9fb2-44ca-41e7-931d-b544a9a02b86/",
"model_version": "0.4.3"
}

Size Exclusion Chromatography Trace

{
"url": "local://cript/file/ef2993c4-b687-4352-99cd-ac1280c5c0f6/",
"uid": "ef2993c4-b687-4352-99cd-ac1280c5c0f6",
"public": false,
"created_at": "2022-09-08T13:48:10.418368",
"updated_at": "2022-09-08T13:48:10.418368",
"project": "local://cript/project/ef26f322-8e03-4655-9aca-a731b6e5ae36/",
"data": [
"local://cript/data/b767d542-d319-4040-a028-934f099441e9/"
],
"type": "data",
"name": "sec.txt",
"checksum": "ec7a6e7598dc3c34cc954f461b132b71a8d43fd4b86752852c3ac187ac62cac7",
"unique_name": null,
"extension": ".txt",
"source": "C:/Users/nicep/Desktop/pyth_proj/cript/examples/test_data/sec.txt",
"group": "local://cript/group/5a5a9fb2-44ca-41e7-931d-b544a9a02b86/",
"model_version": "0.4.3"
}

REFERENCE

{
"url": "local://cript/reference/c8f4cd85-b717-4945-b862-319d37930705/",
"uid": "c8f4cd85-b717-4945-b862-319d37930705",
"public": false,
"created_at": "2022-09-12T17:47:11.083907",
"updated_at": "2022-09-12T17:47:11.083907",
"title": "Kinetics of Anionic Polymerization of Styrene in Tetrahydrofuran",
"doi": "10.1021/ja00872a012",

"authors": [
"Geacintov, C.",
"Smid, J.",
"Szwarc, M."
],
"journal": "Journal of the American Chemical Society",
"publisher": null,
"year": 1962,
"volume": 84,
"issue": 13,
"pages": [
2508
],
"issn": null,
"arxiv_id": null,
"pmid": null,
"website": null,
"notes": null,
"group": "local://cript/group/875316df-1f7d-47ac-aa18-1030cf07d5c7/",
"model_version": "0.4.3"
}
